# Dual-State Emission of 2-(Butylamino)Cinchomeronic Dinitrile Derivatives

**DOI:** 10.3390/molecules27217144

**Published:** 2022-10-22

**Authors:** Konstantin V. Lipin, Mikhail Yu. Ievlev, Anastasiya I. Ershova, Oleg V. Ershov

**Affiliations:** Department of Organic and Pharmaceutical Chemistry, Chuvash State University, Moskovsky pr. 15, Cheboksary 428015, Russia

**Keywords:** cyano compounds, aminopyridines, dual-state emission, fluorescent dyes, cinchomeronic dinitriles

## Abstract

New representatives of 2-(butylamino)cinchomeronic dinitrile derivatives were synthesized as promising fluorophores showing dual-state emission. To characterize the influence of the length (from methyl to butyl) and the structure (both linear and branched) of the alkyl substituent at the amino nitrogen atom, the spectral fluorescence properties of all synthesized compounds were carefully studied both in solution and in solid state. The highest photoluminescence quantum yield values of 63% were noted for solutions of 2-(butylamino)-6-phenylpyridine-3,4-dicarbonitrile in DCM and 2-(butylamino)-5-methyl-6-phenylpyridine-3,4-dicarbonitrile in toluene.

## 1. Introduction

The development of new luminescent organic compounds has received continued attention due to their possible applications in many fields. The most promising fluorophores are compounds efficiently emitting visible light upon photoexcitation both in solution and in the solid state. Such a phenomenon is called dual-state emission (DSE) [1,2,3,4]. Usually, most fluorescent materials are emissive in one state only (in solution or in crystals). In many cases, luminogens exhibit an intense photoluminescence in dilute solutions and show no or weak emission in the solid state [5,6]. This is due to the fact that aggregated molecules are often affected by strong π-π stacking leading to energy exchange and aggregation-caused quenching (ACQ). In contrast to such fluorophores, some organic molecules are non- or weakly emissive in solution but demonstrate a so-called aggregation-induced emission (AIE)—strong fluorescence in the solid state [7,8,9]. The mentioned aggregation state restrictions significantly reduce the areas of possible use for most fluorescent molecules. Thus, the possibility to achieve strong emissions both in solution and in solid state simultaneously for the single molecule is a challenging task since the absorbed excitation energy can be released through various competing channels.

Over the past decade, there has been an exponential increase in the number of publications about the synthetic approaches to compounds showing dual-state emission (DSE) [1,2,3,4,10,11,12,13,14,15,16,17,18,19,20,21,22,23,24,25,26,27,28,29,30,31,32,33,34,35,36,37,38,39,40,41,42,43,44]. This is due to the fact that DSE molecules are more versatile and are therefore intended for a wider range of applications. For example, they are successfully used in sensing (as fluorescence sensors and pH or ion indicators) [20,21,22,23,24,25], bioimaging [26,27,28], ultrahigh-density data recording and storage [29], super-resolution fluorescence microscopy [30], lasing [31], organic OLED devices [32,33,34,35], organic semiconductors [36], and NLO materials [37,38].

Previously, we reported that compounds with cinchomeronic dinitrile fragments exhibited fluorescence both in solutions and in solid state [39,40,41,42,43]. For example, sulfur-containing cinchomeronic dinitrile derivatives (ethyl 2-((3,4-dicyanopyridine-2-yl)thio)acetates) showed blue fluorescence in organic solvents and a green emission in powders [39]. Compounds such as 2-diethylaminocinchomeronic dinitriles were strongly emissive in the solid state and in nonpolar solvents, with fluorescence quantum yields up to 59% [40]. Compounds such as 2-(pyrrolidin-1-yl)-, 2-(piperidin-1-yl)-, and 2-(azepan-1-yl)-pyridine-3,4-dicarbonitrile derivatives showed even higher emission efficiencies both in solvents and in crystals up to 80% [41]. In addition, 2-(dicyanomethylene)-1,2-dihydropyridine-3,4-dicarbonitrile derivatives (4-CN-TCPy) possessed solid-state emissions in the red and NIR regions, while their solutions were characterized with strong solvatochromic effects and blue fluorescence [42]. It was also shown that the fluorescence of 4-CN-TCPy can be used for determination of the degree of substitution of the amino nitrogen atom with ethyl groups using gaseous ethylamine, diethylamine, and triethylamine [43]. Such above-mentioned promising fluorescence properties prompted us to continue our studies. Herein, we report our novel findings in the field of cinchomeronic dinitrile derivatives showing dual-state emissions (DSE): synthesis and characterization of new compounds along with investigation of their photophysical properties.

It is known that alkyl chains of a certain length and other terminal bulky groups are usually introduced not only to improve the solubility of conjugated fluorescent molecules, but also to prevent their intermolecular interactions and to provide “self-isolation” of fluorophores, increasing the emission intensity in both states [1,12,44]. According to this, we decided to synthesize previously undescribed *N*-butylamino-substituted derivatives of cinchomeronic dinitrile bearing both linear and branched alkyl chains and to study their photophysical properties.

## 2. Results

### 2.1. Synthesis and Photophysical Properties of 2-(Butylamino)Pyridine-3,4-Dicarbonitriles 2

**Scheme 1:**
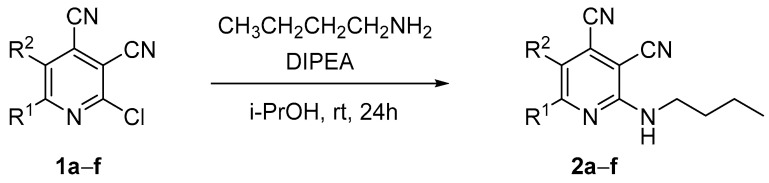
Synthesis of 2-(butylamino)pyridine-3,4-dicarbonitriles **2**.

At first, we developed the method for the preparation of 2-butylaminopyridine-3,4-dicarbonitriles **2a–f** (Scheme 1, Table 1) based on the reaction of available 2-chloropyridine-3,4-dicarbonitriles **1** [45] with *N*-butylamine in propan-2-ol in the presence of *N,N*-diisopropylethylamine (DIPEA). The reaction yield was 53–81%.

**Figure 1:**
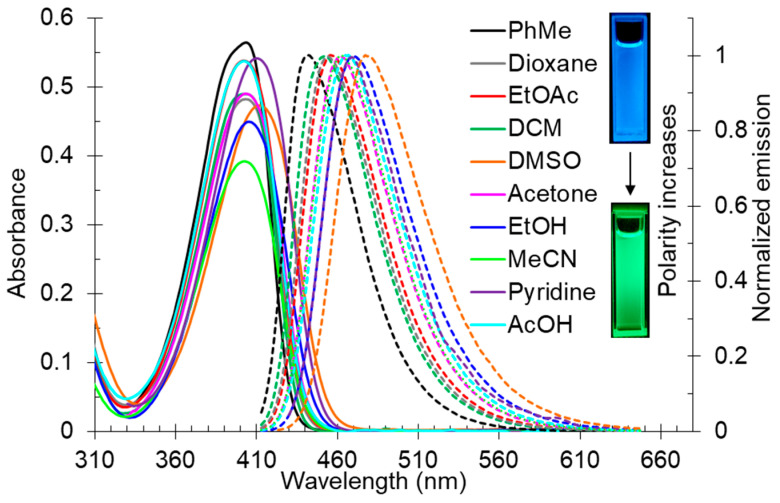
UV-vis absorption (5 × 10^−5^ M, solid lines) and emission (5 × 10^−5^ M, dashed lines) spectra of compound **2d** in various solvents. Photos of solution **2d** in toluene and DMSO were taken under 365 nm of irradiation.

The solvatochromic behavior of 2-butylamino-substituted compounds **2** was studied using compound **2d** (Table 2, Figure 1). It was found that the long-wavelength absorption band was slightly red-shifted upon increasing of the solvent polarity, and its maximum lay in the range of 402–410 nm. The fluorescence band was more affected by the polarity changes, and its maximum was in the range of 442–477 nm corresponding to the blue and blue-green region of the spectrum.

**Figure 2:**
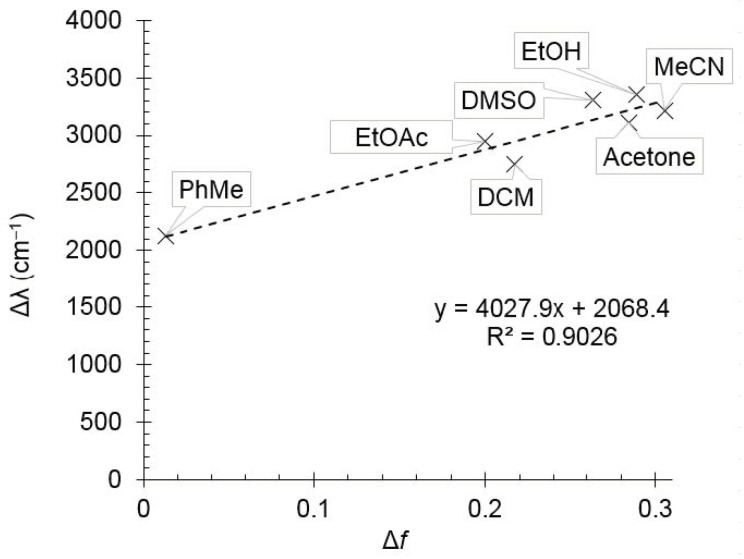
The Lippert–Mataga plot for compound **2d.**

It was found that increasing of the solvent polarity caused a bathochromic shift in the emission maximum. It means that the excited state of compound **2d** is more polar than the ground one, and therefore it should be better stabilized by polar solvents. This observation was supported by the Lippert–Mataga plot showing good linearity (Figure 2). The compound 1,4-dioxane was excluded from the plot due to its “effective” dielectric constant which should be considered much higher as the molecule is able to adopt the boat conformation around dipolar species. Pyridine and acetic acid were also excluded because of the pronounced acid–base properties.

Table 1 describes the relationship between the nonradiative excitation energy loss (Δλ) and the polarity function of a solvent (Δ*f*).

Δλ = (2 Δ*μ*^2^/*hca*^3^) Δ*f*, (1)

where Δλ is Stokes shift value in cm^−1^, Δ*μ* is the change in dipole moment upon photoexcitation, *h* is Planck’s constant, *c* is the speed of light in a vacuum, *a* is the cavity radius of fluorophore, and Δ*f* is the orientation polarizability of the solvent which can be found from:

Δ*f* = [(*ε* − 1)/(2*ε* + 1)] − [(*n*^2^ − 1)/(2*n*^2^ + 1)], (2)

where *ε* is the dielectric constant and *n* is the refractive index of the solvent.

The slope of the Lippert–Mataga plot was used to estimate the change in dipole moment of a molecule upon photoexcitation (Δ*μ*), and for compound **2d** it was found to be about 5.1 D, which can be attributed to the prevailing locally excited (LE) state.

Due to the higher dipole moment in the excited state, the photoluminescence quantum yield of compound **2d** was also expected to be decreased in polar media. Thus, in nonpolar toluene and dichloromethane, it reached 60% and 63%, respectively, while in DMSO, it decreased to 26%. Basic pyridine led to almost complete fluorescence quenching, apparently due to deprotonation of the N–H fragment.

**Figure 3:**
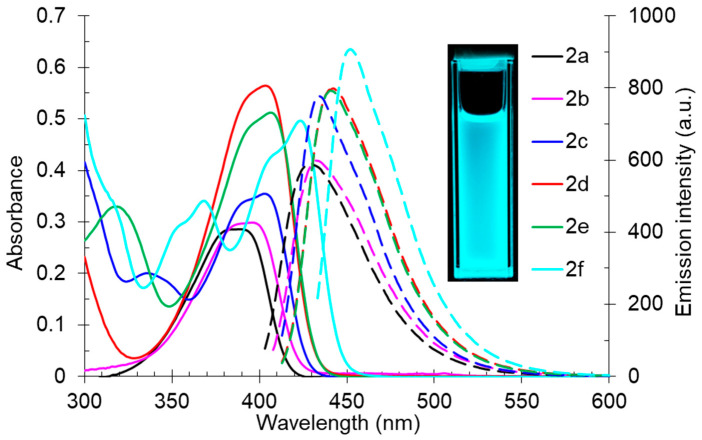
UV-vis absorption (5 × 10^−5^ M, solid lines) and emission (5 × 10^−5^ M, dashed lines) spectra of compound **2** in toluene. A photo of solution **2f** in toluene was taken under 365 nm of irradiation.

The study of the influence of compound **2’s** structure on the photophysical properties showed that derivatives **2a** and **2b,** bearing aliphatic fragments at the pyridine ring, had shorter-wavelength absorption maxima at 389 nm and 396 nm, respectively, while for aryl-substituted structures **2c**–**f**, these band maxima were in the range of 403–423 nm. All the studied compound **2s** demonstrated good photoluminescence properties. Their emission maxima lay in the blue region of the spectrum between 429–452 nm. It should also be noted that the fluorescence quantum yield (Φ_s_) was not decreased, even for aliphatic derivatives **2a,b**. It indicates that the 2-butylamino-substituted cinchomeronic dinitrile moiety is essential for the radiative relaxation from the excited state. The maximum photoluminescence efficiency (Φ_s_) was noted for compound **2c** of about 63% (Table 3, Figure 3).

**Figure 4:**
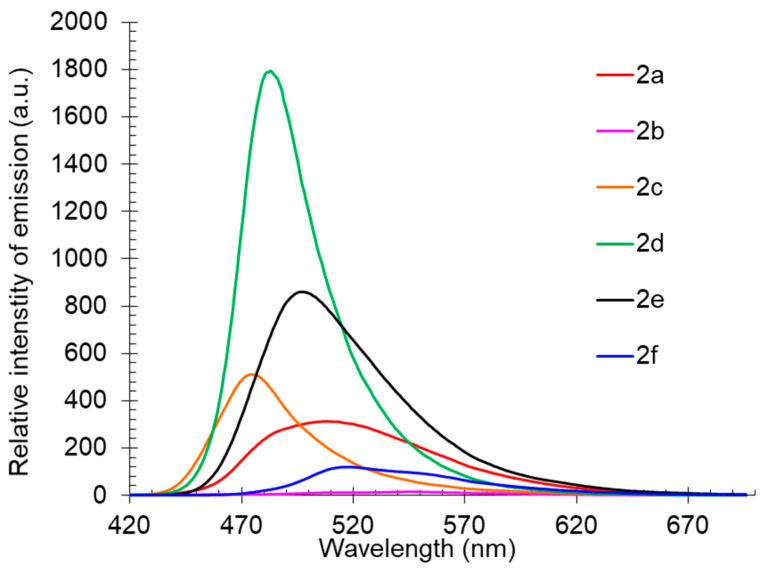
Solid-state photoluminescence spectra of compound **2** in a powder.

Compound **2** also showed good photoluminescence in the individual form in the crystalline state (Table 3, Figure 4). It was found that the emission maxima of the compounds lay in the range of 474–547 nm, corresponding to a wide range from the blue-green to yellow-orange regions of the visible spectrum, and in most cases, short-wavelength emissions were much more intense. These observations indicated that derivatives **2b** and **2f,** showing red-shifted emission maxima with pronounced shoulders, were prone to aggregation with subsequent fluorescence quenching, while compound **2d**, on the contrary, demonstrated the highest photoluminescence intensity and a narrow emission band.

### 2.2. Synthesis and Photophysical Properties of 2-(Butyl(methyl)amino)Pyridine-3,4-Dicarbonitriles 3

The next part of the presented study was the synthesis of compounds bearing a butyl substituent without the mobile N–H moiety. This proton can participate in amine–imine tautomerism and affects the optical properties of compound **2**. The synthesis of target compound **3** bearing *N*-butyl-*N*-methylamine fragments was carried out using two approaches.

**Scheme 2:**
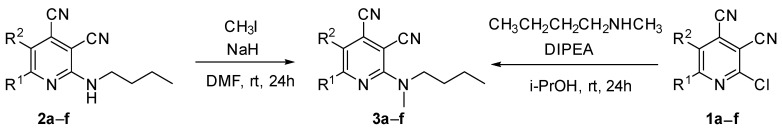
Synthesis of 2-(butyl(methyl)amino)pyridine-3,4-dicarbonitriles **3.**

The first method was developed based on the NH alkylation of compound **2** with methyl iodide in the presence of sodium hydride (Scheme 2) in absolute DMF. The isolated yield of 2-(butyl(methyl)amino)pyridine-3,4-dicarbonitriles 3 was about 12–37%. The second preparation method was based on the halogen substitution reaction in pyridines. As a result of the reaction of 2-chloropyridine-3,4-dicarbonitriles 1 with *N*-butyl-*N*-methylamine in propan-2-ol in the presence of *N*,*N*-diisopropylethylamine (DIPEA), compounds 3a–f were synthesized with 71–96% yields (Scheme 2, Table 1).

**Figure 5:**
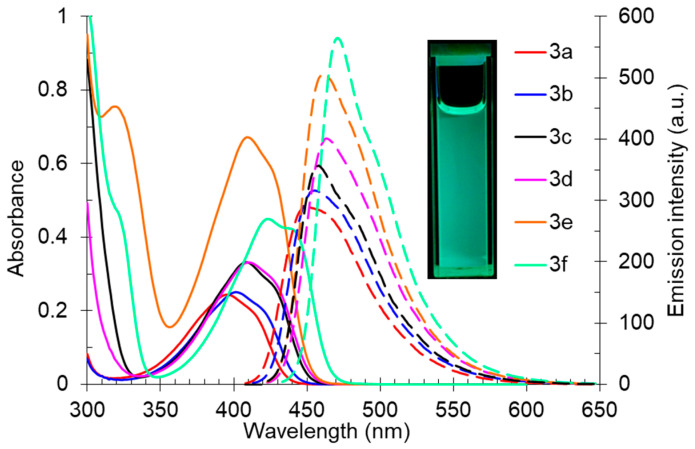
UV-vis absorption (5 × 10^−5^ M, solid lines) and emission (5 × 10^−5^ M, dashed lines) spectra of compound **3** in toluene. A photo of solution **3f** in toluene was taken under 365 nm of irradiation.

The synthesized 2-(butyl(methyl)amino)pyridine-3,4-dicarbonitriles **3** also exhibited a pronounced fluorescence in solution. The registered absorption spectra of compound **3** in toluene were characterized by maxima in the range of 395–439 nm, and the emission spectra showed an intense fluorescence band in the range of 449–471 nm, corresponding to the blue region of the spectra (Table 3, Figure 5). The shape of the photoluminescence spectra was characterized by a pronounced long-wavelength shoulder, indicating the probability of the existence of several radiating excited states. The Stokes shift values (Δλ) correlated with the observed photoluminescence quantum yield values (Φ_s_). Thus, for compound **3a**, significant nonradiative energy losses (3045 cm^–1^) were observed leading to a decrease in Φ_s_ to 36%. In turn, for the derivative **3f**, which was characterized by a bathochromic shift in both the absorption and emission bands, the quantum yield reached 47%, and the Stokes shift was the smallest one of 32 nm (1548 cm^−1^).

**Figure 6:**
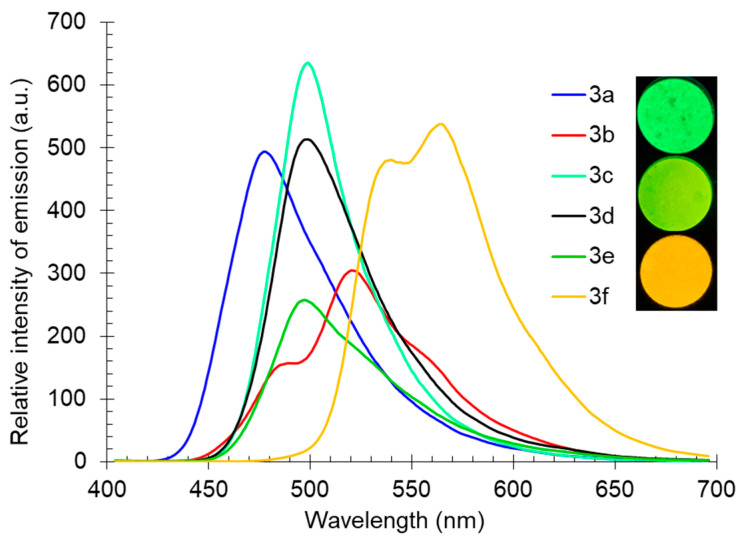
Solid-state photoluminescence spectra of compound **3** in a powder. Photos of compounds **3c**, **3e,** and **3f** were taken under 365 nm of irradiation.

The fluorescence spectra of compound **3**, registered at room temperature for powders of the studied substances, covered a wide range from the blue-green to yellow-orange spectral regions, with maxima in the range of 478–564 nm (Table 3, Figure 6). Compounds **3b** and **3f,** bearing a six-membered ring fused with pyridine, were characterized by the presence of several emission maxima, which were associated with different types of crystal packings in solid samples. The highest intensity of the solid-state photoluminescence upon excitation at 365 nm was noted for compounds **3a**, **3c**, **3d**, and **3f**.

### 2.3. Synthesis and Photophysical Properties of 2-(Dibutylamino)Pyridine-3,4-Dicarbonitriles 4

**Scheme 3:**
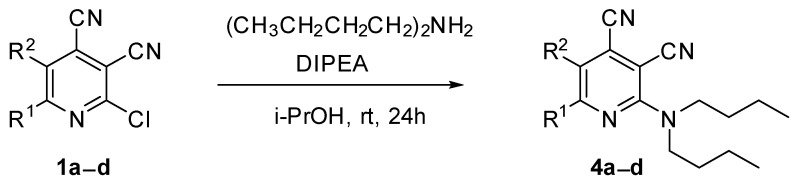
Synthesis of 2-(dibutylamino)pyridine-3,4-dicarbonitriles **4.**

In the next step, the method for preparation of compounds bearing two bulky alkyl substituents was developed. It was found that the reaction of 2-chloropyridine-3,4-dicarbonitriles **1** with *N,N-*dibutylamine in propan-2-ol in the presence of *N,N-*diisopropylethylamine (DIPEA) gave 2-dibutylaminopyridine-3,4-dicarbonitriles **4a**–**d** (Scheme 3, Table 1), with 43–77% yields. The method based on the *N-*alkylation of compound **2** with butyl halides gave extremely poor yields (less than 10%).

**Figure 7:**
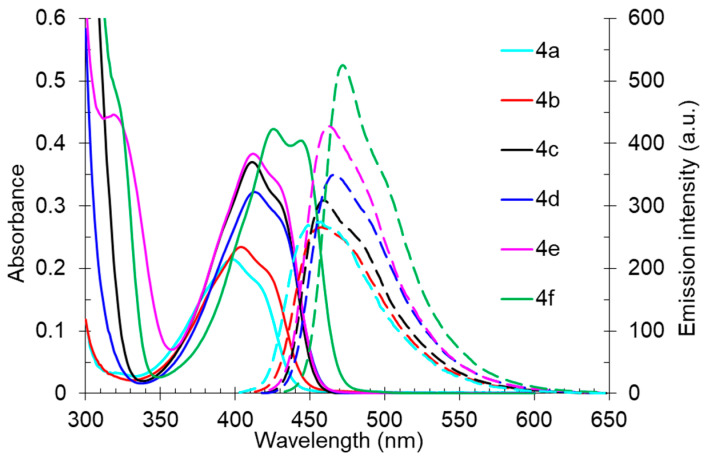
UV-vis absorption (5 × 10^−5^ M, solid lines) and emission (5 × 10^−5^ M, dashed lines) spectra of compound **4** in toluene.

Studies of the photophysical properties of the 2-dibutylamino-substituted derivatives **4** showed that the absorption maxima of these compounds in toluene were in the range of 398–426 nm and were also characterized by intermediate values of light absorption coefficients (4300–8460 M^−1^ cm^−1^) (Table 3, Figure 7). The expected bathochromic shift in the absorption band along with an increase in intensity was observed for aryl-substituted derivatives **4c**–**f**. Compound **4f**, containing a spatially locked aromatic fragment, showed the biggest red shift and hyperchromic effect. The fluorescence bands of compound **4** were also in the blue region of the spectra, with maxima in the range of 456–472 nm, that also showed a bathochromic shift for aryl-substituted derivatives **4c**–**f**. The photoluminescence quantum yields were in the range from 33% to 43%, and the highest emission intensity was also noted for compound **4f** with fused cyclic fragments. The Stokes shift values (2288–3196 cm^−1^) showed the expected inverse correlation with the emission efficiency, and the shape of the emission bands was also characterized by a pronounced long-wavelength shoulder, probably caused by another radiative excited state.

**Figure 8:**
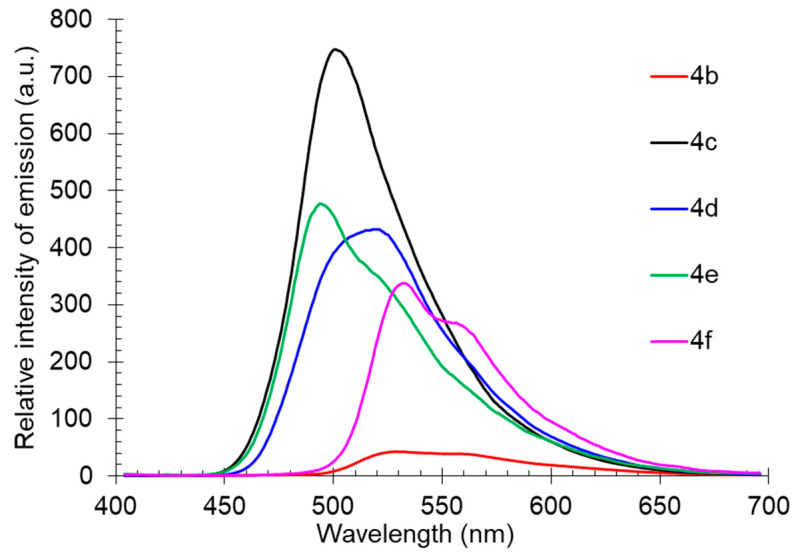
Solid-state photoluminescence spectra of compound **4** in a powder.

The solid-state photoluminescence was registered at room temperature for the powder samples of compounds **4b-f** (compound **4a** is liquid at rt). It was found that excitation by UV (365 nm) caused fluorescence of compound **4** with emission maxima in the green-yellow region of the spectra between 494–532 nm. Compound **4c** showed the highest emission intensity; all other compounds observed slight quenching, probably caused by self-aggregation. It was supported by the shape of the fluorescence spectra showing a pronounced long-wavelength shoulder (Table 3, Figure 8).

### 2.4. Synthesis and Photophysical Properties of 2-(Diisobutylamino)Pyridine-3,4-Dicarbonitriles 5

**Scheme 4:**
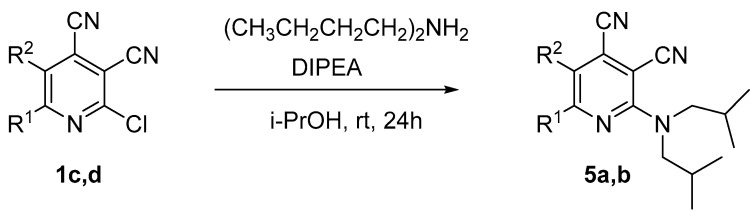
Synthesis of 2-(diisobutylamino)pyridine-3,4-dicarbonitriles **5.**

The next part of the presented study was the synthesis of compounds bearing branched alkyl substituents. It was found that the reaction of 2-chloropyridine-3,4-dicarbonitriles **1** with diisobutylamine in propan-2-ol in the presence of *N,N*-diisopropylethylamine (DIPEA) gave 2-diisobutylaminopyridine-3,4-dicarbonitrile derivatives **5a,b,** with yields of 48–56% (Scheme 4, Table 4).

**Figure 9:**
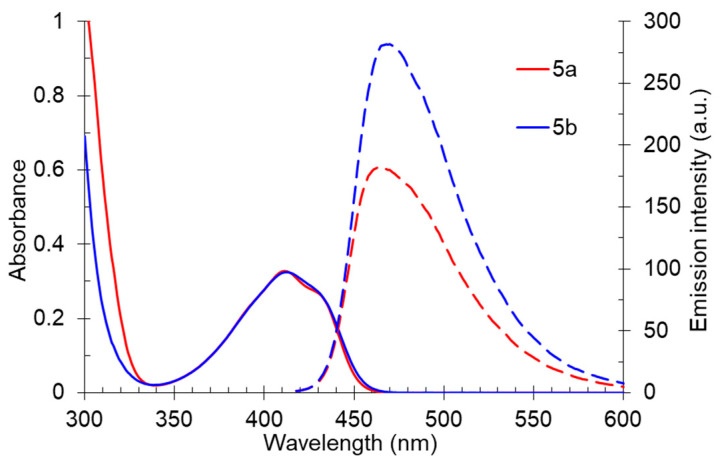
UV-vis absorption (5 × 10^−5^ M, solid line) and emission (5 × 10^−5^ M, dashed line) spectra of compound **5** in toluene.

To study the effect of the branching alkyl fragment of the substituted amino group on the spectral properties, the electronic spectra of 2-diisobutylamino-substituted pyridines **5** were analyzed (Table 3, Figure 9). It was found that their absorption maxima were in a narrow range of 411–413 nm, and the emission maxima were between 464–470 nm. Moreover, it should be noted that the 5-methyl-substituted derivative **5b** was characterized with a high photoluminescence quantum yield, reaching 33% in a toluene solution. For compound **5a**, the fluorescence efficiency was 23%. Apparently, the presence of the methyl group at the C5 atom of the pyridine ring prevented the planarity of the molecule; as a result, the efficiency of conjugation between aromatic fragments was decreased. Apparently, it hindered the process of deactivation of the excited state through intramolecular charge transfer and increased the probability of radiative relaxation. The Stokes shift values (2779–2936 cm^−1^) also indicated a significant nonradiative loss of excitation energy, which was associated with an increase in the contribution of vibrational relaxation due to an increase in the number of mobile moieties.

**Figure 10:**
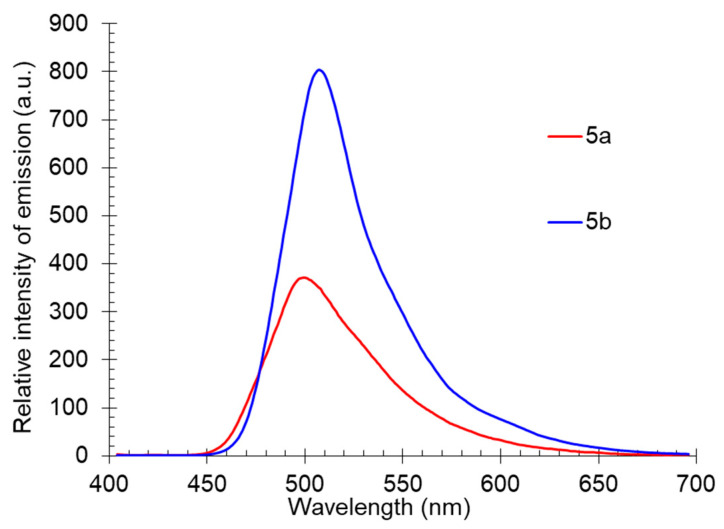
Solid-state photoluminescence spectra of compound **5** in a powder.

The solid-state photoluminescence spectra of compounds **5** were registered in powders at room temperature (excitation wavelength was 365 nm). The spectra demonstrated a similar dependence with solutions: the 5-methyl-substituted derivative **5b** was characterized by a higher fluorescence intensity. The maxima of the solid-state emission bands of both compounds were in the yellow region of the spectra (500 nm and 507 nm) and did not show a pronounced “shoulder” (Table 3, Figure 10).

### 2.5. Synthesis of N-Alkyl-2-(Butylamino)Pyridine-3,4-Dicarbonitriles 6

**Scheme 5:**
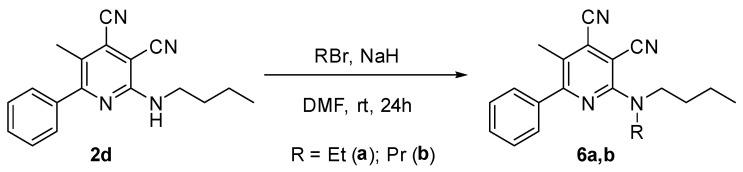
Synthesis of 2-((alkyl)butylamino)pyridine-3,4-dicarbonitriles **6.**

One of the tasks of the presented study was to establish the effects of the length of the alkyl substituent at the amino group on the photophysical properties of cinchomeronic dinitrile derivatives. Therefore, we directly prepared a series of *N*-substituted derivatives of 2-butylamino-5-methyl-6-phenylpyridine-3,4-dicarbonitrile **2d**, **3d**, and **4d** and additional *N*-ethyl (**6a**) and *N*-propyl (**6b**) derivatives. They were obtained by the reaction of 2-butylamino-5-methyl-6-phenylpyridine-3,4-dicarbonitrile **2d** with bromoethane or bromopropane in absolute DMF in the presence of sodium hydride (Scheme 5, Table 4).

## 3. Discussion

**Figure 11:**
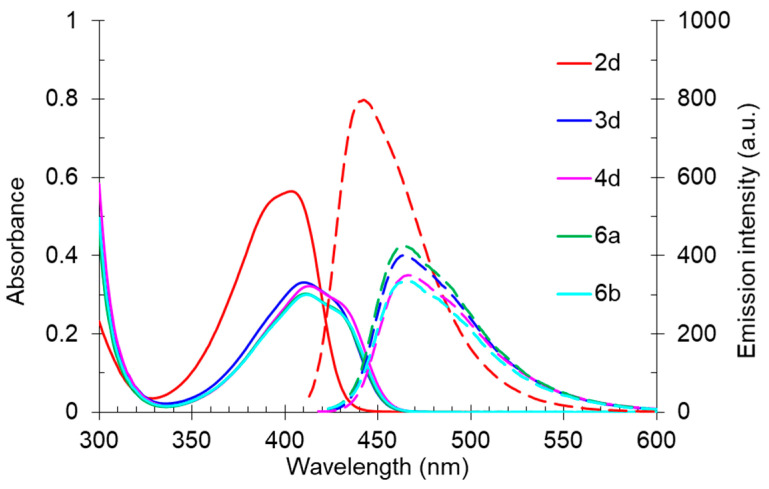
UV-vis absorption (5 × 10^–5^ M, solid line) and emission (5 × 10^–5^ M, dashed line) spectra of compounds **2d, 3d, 4d, 6a,** and **6b** in toluene.

A study of the optical properties showed that the highest fluorescence intensity was observed for compound **2d**, bearing a free NH fragment. Its photoluminescence quantum yield reached 60% in a toluene solution. A replacement of a hydrogen atom of NH moiety by alkyl substituents of various lengths led to a decrease in the photoluminescence efficiency, down to 37%, and that decrease occurred with an increase in the alkyl chain per carbon atom. Moreover, a slight bathochromic shift in the absorption bands from 403 nm (derivative **2d**) to 410–413 nm (compounds **3d**, **6a**, **6b**, **4d**) also occurred (Figure 11). A similar trend was also observed for the emission band, shifting from 442 nm to the region of 463–466 nm.

**Figure 12:**
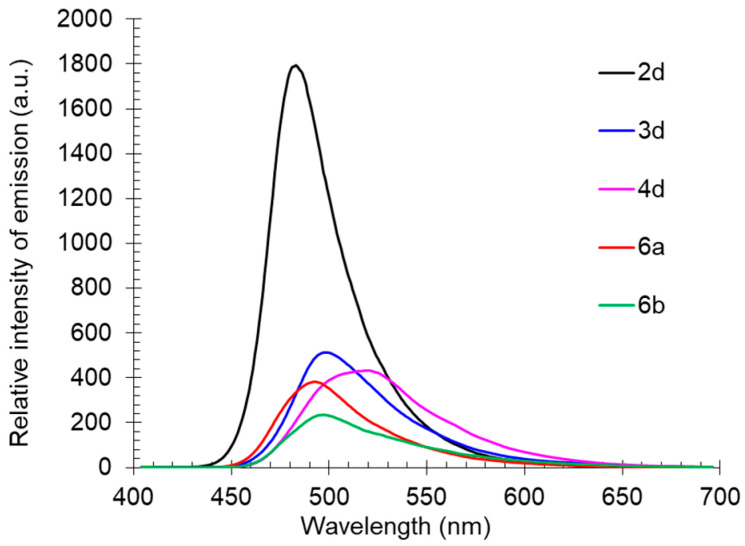
Solid-state photoluminescence spectra of compounds **2d, 3d, 4d, 6a,** and **6b** in a powder.

The solid-state photoluminescence spectra of compounds **2d**, **3d**, **6a**, **6b,** and **4d,** containing various substituents at the nitrogen atom of the amino group, were characterized by emission maxima in the range of 483–519 nm. Compound **2d,** with a free NH fragment was the most intense, similarly as in solution. The introduction of an alkyl substituent into the amino group led to a bathochromic shift in the emission band and caused a decrease in its intensity (Table 3, Figure 12).

Thus, it can be concluded that the presence of a free NH fragment in the studied compounds is essential for the intense photoluminescence both in solution and in solid state (dual-state emission). The introduction of a second substituent into the amino group, as well as an increase in its size, lead to a decrease in the fluorescence efficiency (Table 3, Figure 11).

## 4. Materials and Methods

The progress of reactions and the purity of products were monitored by thin-layer chromatography (TLC) on Sorbfil plates (spots were visualized under UV light, by treatment with iodine vapor, or by heating). Melting points were determined using an OptiMelt MPA100 device. The IR spectra were recorded on an FSM-2201 spectrometer with Fourier transforms from samples dispersed in mineral oil. The NMR spectra were measured a DMSO-*d*_6_ on Bruker AV-500 spectrometer using tetramethylsilane or residual solvent peak as the internal references. Copies of the NMR spectra are provided in supplementary materials. Elemental analyses were performed using a FlashEA 1112 CHN analyzer. The mass spectra (electron impact, 70 eV) were obtained on a Shimadzu GCMS-QP2020 using a direct-probe inlet. The UV spectra were recorded on an Agilent Cary 60 UV-Vis spectrophotometer using a standard quartz cuvette with a pathlength of 1 cm. The fluorescence spectra were recorded on an Agilent Cary Eclipse spectrofluorometer using a quartz cuvette with four optically clear sides. The relative fluorescence quantum yields in solution (Φ_s_) were evaluated by the comparative method using quinine sulfate in 0.05 M H_2_SO_4_ as the standard compound with a known fluorescence efficiency (Φ_std_ = 60%, excited at 380 nm) [46] by the creation of a calibration curve, plotting the area of fluorescence against the absorbance for different concentrations of the fluorophore. The solid-state emission spectra were registered at room temperature for sample powders using the Agilent Cary Eclipse solid sample holder. Photoluminescence quantum yields in the crystalline state (Φ_c_) were determined by Wrighton’s method [47] using 365 nm of excitation.

### 4.1. General Procedure for the Preparation of 2-(Butylamino)Pyridine-3,4-Dicarbonitriles 2

An appropriate 2-chloropyridine-3,4-dicarbonitrile **1** (0.01 mol) was suspended in *i-*PrOH (5 mL), and then *N*-butylamine (0.80 g, 0.011 mol) and DIPEA (1.42 g, 0.011 mol) were added. The reaction mixture was stirred at 60–70 °C for 24 h. After reaction completion (TLC monitoring), the mixture was cooled, and the precipitated product was filtered off and washed with ice-cold water and *i-*PrOH. The resulting product was crystallized from *i-*PrOH, and then dried in a vacuum desiccator over CaCl_2_.

Compound **2a**: 2-(Butylamino)-5,6-dimethylpyridine-3,4-dicarbonitrile. Yield 53%, mp 160–161 °C. IR (mineral oil, cm^−1^): 3377 (NH); 2233, 2214 (C≡N); 1591 (C=C). ^1^H NMR (500.13 MHz, DMSO-d_6_): δ 0.89 (t, *J* = 7.4 Hz, 3H, CH_2_CH_3_), 1.25–1.33 (m, 2H, CH_2_CH_3_), 1.48–1.54 (m, 2H, CH_2_CH_2_), 2.25 (s, 3H, CH_3_), 2.40 (s, 3H, CH_3_), 3.34–3.39 (m, 2H, CH_2_NH), 7.38 (br t, 1H, NH). ^13^C NMR (125.76 MHz, DMSO-d_6_): δ 13.5, 15.8, 19.3, 23.7, 30.6, 40.2, 86.6, 114.5, 114.9, 121.0, 123.3, 156.1, 163.3. MS (EI, 70 eV): *m*/*z* (%) 228 ([M]^+^, 25), 185 ([M-C_3_H_7_]^+^, 100). Anal: Calcd for C_13_H_16_N_4_: C, 68.39; H, 7.06; N, 24.54. Found: C, 68.18; H, 7.03; N, 24.64.

Compound **2b**: 2-(Butylamino)-5,6,7,8-tetrahydroquinoline-3,4-dicarbonitrile. Yield 54%, mp 149–150 °C. IR (mineral oil, cm^−1^): 3367 (NH); 2214 (C≡N); 1582 (C=C). ^1^H NMR (500.13 MHz, DMSO-d_6_): δ 0.88 (t, *J* = 7.3 Hz, 3H, CH_2_CH_3_), 1.25–1.32 (m, 2H, CH_2_CH_3_), 1.45–1.54 (m, 2H, CH_2_), 1.71–1.79 (m, 4H, 2CH_2_), 2.66–2.72 (m, 4H, 2CH_2_), 3.33–3.37 (m, 2H, CH_2_NH), 7.36 (br t, 1H, NH). ^13^C NMR (125.76 MHz, DMSO-d_6_): δ 13.7, 19.46, 21.6, 21.6, 25.9, 30.7, 33.0, 40.3, 87.9, 114.0, 114.8, 121.8, 123.9, 155.9, 163.4. MS (EI, 70 eV): *m*/*z* (%) 254 ([M]^+^, 22), 211 ([M-C_3_H_7_]^+^, 100). Anal: Calcd for C_15_H_18_N_4_: C, 70.84; H, 7.13; N, 22.03. Found: C, 70.51; H, 7.02; N, 22.13.

Compound **2c**: 2-(Butylamino)-6-phenylpyridine-3,4-dicarbonitrile. Yield 65%, mp 151–152 °C. IR (mineral oil, cm^−1^): 3393 (NH); 2242, 2213 (C≡N); 1588 (C=C). ^1^H NMR (500.13 MHz, DMSO-d_6_): δ 0.91 (t, *J* = 7.4 Hz, 3H, CH_2_CH_3_), 1.28–1.39 (m, 2H, CH_2_CH_3_), 1.54–1.65 (m, 2H, CH_2_), 3.47–3.54 (m, 2H, CH_2_NH), 7.50–7.55 (m, 3H, Ph), 7.76 (s, 1H, CH_pyr_), 7.82 (t, *J* = 5.6 Hz, 1H, NH), 8.11–8.17 (m, 2H, Ph). ^13^C NMR (125.76 MHz, DMSO-d_6_): δ 13.8, 19.6, 30.6, 40.6, 88.9, 110.2, 114.7, 115.3, 125.3, 127.3, 128.9, 131.2, 136.3, 157.7, 159.6. MS (EI, 70 eV): *m*/*z* (%) 276 ([M]^+^, 37), 233 ([M-C_3_H_7_]^+^, 100). Anal: Calcd for C_17_H_16_N_4_: C, 73.89; H, 5.84; N, 20.27. Found: C, 74.01; H, 5.81; N, 20.18.

Compound **2d**: 2-(Butylamino)-5-methyl-6-phenylpyridine-3,4-dicarbonitrile. Yield 74%, mp 141–142 °C. IR (mineral oil, cm^−1^): 3361 (NH); 2231, 2217 (C≡N); 1586 (C=C). ^1^H NMR (500.13 MHz, DMSO-d_6_): δ 0.86 (t, *J* = 7.4 Hz, 3H, CH_2_CH_3_), 1.25–1.32 (m, 2H, CH_2_CH_3_), 1.49–1.58 (m, 2H, CH_2_), 2.31 (s, 3H, CH_3_), 3.35–3.39 (m, 2H, CH_2_NH), 7.48–7.51 (m, 3H, Ph), 7.54–7.57 (m, 2H, Ph), 7.60 (t, *J* = 5.6 Hz, 1H, NH). ^13^C NMR (125.76 MHz, DMSO-d_6_): δ 13.7, 17.4, 19.5, 30.7, 40.5, 89.1, 114.7, 114.8, 120.2, 125.8, 128.2, 128.8, 129.3, 138.5, 156.1, 162.2. MS (EI, 70 eV): *m*/*z* (%) 290 ([M]^+^, 43), 247 ([M-C_3_H_7_]^+^, 100). Anal: Calcd for C_18_H_18_N_4_: C, 74.46; H, 6.25; N, 19.30. Found: C, 74.59; H, 6.21; N, 19.24.

Compound **2e**: 2-(Butylamino)-6-(4-methoxyphenyl)-5-methylpyridine-3,4-dicarbonitrile. Yield 63%, mp 125–126 °C. IR (mineral oil, cm^−1^): 3377 (NH); 2234, 2211 (C≡N); 1585 (C=C). ^1^H NMR (500.13 MHz, DMSO-d_6_): δ 0.88 (t, *J* = 7.4 Hz, 3H, CH_2_CH_3_), 1.26-1.34 (m, 2H, CH_2_CH_3_), 1.51–1.57 (m, 2H, CH_2_), 2.35 (s, 3H, CH_3_), 3.36–3.42 (m, 2H, CH_2_NH), 3.83 (s, 3H, OCH_3_), 7.02–7.09 (m, 2H, C_6_H_4_), 7.50–7.61 (m, 3H, C_6_H_4_+NH). ^13^C NMR (125.76 MHz, DMSO-d_6_): δ 14.3, 18.3, 20.1, 31.4, 41.1, 55.9, 88.9, 114.2, 115.4, 115.6, 120.7, 126.3, 131.3, 131.4, 156.6, 160.9, 162.3. MS (EI, 70 eV): *m*/*z* (%) 320 ([M]^+^, 51), 277 ([M-C_3_H_7_]^+^, 100). Anal: Calcd for C_19_H_20_N_4_O: C, 71.23; H, 6.29; N, 17.49. Found: C, 71.44; H, 6.32; N, 17.38.

Compound **2f**: 2-(Butylamino)-5,6-dihydrobenzo[*h*]quinoline-3,4-dicarbonitrile. Yield 81%, mp 161–162 °C. IR (mineral oil, cm^−1^): 3368 (NH); 2215 (C≡N); 1583 (C=C). ^1^H NMR (500.13 MHz, DMSO-d_6_): δ 0.91 (t, *J* = 7.4 Hz, 3H, CH_2_CH_3_), 1.29–1.39 (m, 2H, CH_2_CH_3_), 1.56–1.63 (m, 2H, CH_2_), 2.86–2.96 (m, 4H, 2CH_2_), 3.45–3.49 (m, 2H, CH_2_NH), 7.30–7.44 (m, 3H, C_6_H_4_), 7.56 (br t, 1H, NH), 8.07–8.12 (m, 1H, C_6_H_4_). ^13^C NMR (125.76 MHz, DMSO-d_6_): δ 14.4, 20.24, 25.4, 27.1, 31.3, 41.2, 88.9, 114.8, 115.7, 122.1, 123.8, 126.1, 127.8, 128.9, 131.9, 132.7, 140.1, 155.9, 157.4. MS (EI, 70 eV): *m*/*z* (%) 302 ([M]^+^, 46), 259 ([M-C_3_H_7_]^+^, 100). Anal: Calcd for C_19_H_18_N_4_: C, 75.47; H, 6.00; N, 18.53. Found: C, 75.22; H, 5.97; N, 18.61.

### 4.2. General Procedure for the Preparation of 2-(Butyl(methyl)amino)Pyridine-3,4-Dicarbonitriles 3

*Method A.* An appropriate 2-butylaminopyridine-3,4-dicarbonitrile **2** (0.01 mol) was dissolved in dry DMF (10 mL), and then NaH (0.52 g, 0.013 mol, 60% in mineral oil) and methyl iodide (2.13 g, 0.015 mol) were added. The reaction mixture was stirred at room temperature for 24 h. After reaction completion (TLC monitoring), the mixture was diluted with water (50 mL) and neutralized by acetic acid. The precipitated solid was filtered off, washed with ice-cold water, and crystallized from *i-*PrOH, and then dried in a vacuum desiccator over CaCl_2_.

*Method B.* An appropriate 2-chloropyridine-3,4-dicarbonitrile **1** (0.01 mol) was suspended in *i-*PrOH (5 mL), and then *N*-butyl-*N*-methylamine (0.96 g, 0.011 mol) and DIPEA (1.42 g, 0.011 mol) were added. The reaction mixture was stirred at 60–70 °C for 24 h. After reaction completion (TLC monitoring), the mixture was cooled, and the precipitated product was filtered off and washed with ice-cold water and *i-*PrOH. The resulting product **3** was crystallized from *i-*PrOH, and then dried in a vacuum desiccator over CaCl_2_.

Compound **3a**: 2-(Butyl(methyl)amino)-5,6-dimethylpyridine-3,4-dicarbonitrile. Yield 17% (method A), 71% (method B), mp 43–44 °C. IR (mineral oil, cm^−1^): 2232, 2206 (C≡N); 1578 (C=C). ^1^H NMR (500.13 MHz, DMSO-d_6_): δ 0.90 (t, *J* = 7.4 Hz, 3H, CH_2_CH_3_), 1.25–1.32 (m, 2H, CH_2_CH_3_), 1.54–1.60 (m, 2H, CH_2_CH_2_), 2.28 (s, 3H, CH_3_), 2.41 (s, 3H, CH_3_), 3.19 (s, 3H, CH_3_N), 3.62 (t, *J* = 7.6 Hz, 2H, CH_2_N). ^13^C NMR (125.76 MHz, DMSO-d_6_): δ 13.7, 16.0, 19.3, 23.7, 29.1, 37.9, 50.8, 85.8, 114.8, 116.9, 122.9, 125.3, 155.9, 162.4. MS (EI, 70 eV): *m*/*z* (%) 242 ([M]^+^, 19), 199 ([M-C_3_H_7_]^+^, 100). Anal: Calcd for C_14_H_18_N_4_: C, 69.39; H, 7.49; N, 23.12. Found: C, 69.18; H, 7.54; N, 23.04.

Compound **3b**: 2-(Butyl(methyl)amino)-5,6,7,8-tetrahydroquinoline-3,4-dicarbonitrile. Yield 12% (method A), 84% (method B), mp 42–43 °C. IR (mineral oil, cm^−1^): 2231, 2212 (C≡N); 1571 (C=C). ^1^H NMR (500.13 MHz, DMSO-d_6_): δ 0.90 (t, *J* = 7.4 Hz, 3H, CH_2_CH_3_), 1.25–1.32 (m, 2H, CH_2_CH_3_), 1.54–1.60 (m, 2H, CH_2_), 1.74–1.80 (m, 4H, 2CH_2_), 2.69–2.74 (m, 4H, 2CH_2_), 3.18 (s, 3H, CH_3_N), 3.61 (t, *J* = 7.6 Hz, 2H, CH_2_N). ^13^C NMR (125.76 MHz, DMSO-d_6_): δ 13.7, 19.3, 21.5, 21.6, 26.0, 29.1, 32.9, 38.0, 50.8, 87.0, 114.2, 116.7, 123.5, 125.7, 155.8, 162.4. MS (EI, 70 eV): *m*/*z* (%) 268 ([M]^+^, 20), 225 ([M-C_3_H_7_]^+^, 100). Anal: Calcd for C_16_H_20_N_4_: C, 71.61; H, 7.51; N, 20.88. Found: C, 71.22; H, 7.55; N, 20.96.

Compound **3c**: 2-(Butyl(methyl)amino)-6-phenylpyridine-3,4-dicarbonitrile. Yield 20% (method A), 87% (method B), mp 69–70 °C. IR (mineral oil, cm^−1^): 2240, 2205 (C≡N); 1588 (C=C). ^1^H NMR (500.13 MHz, DMSO-d_6_): δ 0.92 (t, *J* = 7.4 Hz, 3H, CH_2_CH_3_), 1.29–1.39 (m, 2H, CH_2_CH_3_), 1.60–1.68 (m, 2H, CH_2_), 3.31 (s, 3H, CH_3_N), 3.72 (t, *J* = 7.7 Hz, 2H, CH_2_N), 7.50–7.54 (m, 3H, Ph), 7.86 (s, 1H, CH_pyr_), 8.10–8.14 (m, 2H, Ph). ^13^C NMR (125.76 MHz, DMSO-d_6_): δ 13.7, 19.4, 29.0, 38.2, 51.2, 87.4, 111.4, 115.5, 116.7, 127.3, 127.5, 128.9, 131.2, 135.9, 157.0, 158.5. MS (EI, 70 eV): *m*/*z* (%) 290 ([M]^+^, 29), 247 ([M-C_3_H_7_]^+^, 100). Anal: Calcd for C_18_H_18_N_4_: C, 74.46; H, 6.25; N, 19.30. Found: C, 74.62; H, 6.21; N, 19.11.

Compound **3d**: 2-(Butyl(methyl)amino)-5-methyl-6-phenylpyridine-3,4-dicarbonitrile. Yield 19% (method A), 96% (method B), mp 126–127 °C. IR (mineral oil, cm^−1^): 2237, 2212 (C≡N); 1584 (C=C). ^1^H NMR (500.13 MHz, DMSO-d_6_): δ 0.89 (t, *J* = 7.4 Hz, 3H, CH_2_CH_3_), 1.25–1.33 (m, 2H, CH_2_CH_3_), 1.58–1.64 (m, 2H, CH_2_), 2.35 (s, 3H, CH_3_), 3.25 (s, 3H, CH_3_N), 3.65 (t, *J* = 7.7 Hz, 2H, CH_2_N), 7.48–7.52 (m, 3H, Ph), 7.57–7.60 (m, 2H, Ph). ^13^C NMR (125.76 MHz, DMSO-d_6_): δ 13.7, 17.4, 19.3, 29.1, 38.1, 51.0, 87.8, 114.8, 116.7, 121.8, 127.7, 128.2, 128.9, 129.5, 138.2, 155.7, 161.2. MS (EI, 70 eV): *m*/*z* (%) 304 ([M]^+^, 31), 261 ([M-C_3_H_7_]^+^, 100). Anal: Calcd for C_19_H_20_N_4_: C, 74.97; H, 6.62; N, 18.41. Found: C, 74.69; H, 6.66; N, 18.31.

Compound **3e**: 2-(Butyl(methyl)amino)-6-(4-methoxyphenyl)-5-methylpyridine-3,4-dicarbonitrile. Yield 37% (method A), 79% (method B), mp 118–119 °C. IR (mineral oil, cm^−1^): 2229, 2210 (C≡N); 1582 (C=C). ^1^H NMR (500.13 MHz, DMSO-d_6_): δ 0.90 (t, *J* = 7.4 Hz, 3H, CH_2_CH_3_), 1.26–1.34 (m, 2H, CH_2_CH_3_), 1.58–1.64 (m, 2H, CH_2_), 2.39 (s, 3H, CH_3_), 3.25 (s, 3H, CH_3_N), 3.66 (t, *J* = 7.6 Hz, 2H, CH_2_N), 3.82 (s, 3H, OCH_3_), 7.03–7.06 (m, 2H, C_6_H_4_), 7.58–7.61 (m, 2H, C_6_H_4_). ^13^C NMR (125.76 MHz, DMSO-d_6_): δ 13.7, 17.7, 19.3, 29.1, 38.1, 51.0, 55.3, 87.0, 113.7, 114.9, 116.9, 121.7, 127.6, 130.4, 130.7, 155.7, 160.3, 160.7. MS (EI, 70 eV): *m*/*z* (%) 334 ([M]^+^, 39), 291 ([M-C_3_H_7_]^+^, 100). Anal: Calcd for C_20_H_22_N_4_O: C, 71.83; H, 6.63; N, 16.75. Found: C, 71.57; H, 6.65; N, 16.60.

Compound **3f**: 2-(Butyl(methyl)amino)-5,6-dihydrobenzo[*h*]quinoline-3,4-dicarbonitrile. Yield 14% (method A), 92% (method B), mp 135–136 °C. IR (mineral oil, cm^−1^): 2233, 2203 (C≡N); 1584 (C=C). ^1^H NMR (500.13 MHz, DMSO-d_6_): δ 0.92 (t, *J* = 7.4 Hz, 3H, CH_2_CH_3_), 1.30–1.38 (m, 2H, CH_2_CH_3_), 1.60–1.66 (m, 2H, CH_2_), 2.87–2.98 (m, 4H, 2CH_2_), 3.28 (s, 3H, CH_3_N), 3.70 (t, *J* = 7.6 Hz, 2H, CH_2_N), 7.29–7.48 (m, 3H, C_6_H_4_), 8.08 (d, *J* = 7.7 Hz, 1H, C_6_H_4_). ^13^C NMR (125.76 MHz, DMSO-d_6_): δ 13.7, 19.4, 24.8, 26.4, 29.0, 38.2, 51.2, 87.1, 114.3, 117.0, 123.0, 125.1, 125.5, 127.2, 128.3, 131.4, 131.8, 139.5, 154.4, 156.5. MS (EI, 70 eV): *m*/*z* (%) 316 ([M]^+^, 32), 273 ([M-C_3_H_7_]^+^, 100). Anal: Calcd for C_20_H_20_N_4_: C, 75.92; H, 6.37; N, 17.71. Found: C, 75.54; H, 6.39; N, 17.64.

### 4.3. General Procedure for the Preparation of 2-(Dibutylamino)Pyridine-3,4-Dicarbonitriles 4

An appropriate 2-chloropyridine-3,4-dicarbonitrile **1** (0.01 mol) was suspended in *i-*PrOH (5 mL), and then *N,N*-dibutylamine (1.42 g, 0.011 mol) and DIPEA (1.42 g, 0.011 mol) were added. The reaction mixture was stirred at 60–70 °C for 24 h. After reaction completion (TLC monitoring), the mixture was cooled, the solvent was removed using a rotary evaporator, and the residue was extracted with ethyl acetate. The extract was dried with CaCl_2_ and then purified with column chromatography (ethyl acetate/hexane, 1/1 *v*/*v*). Product **4** was isolated after solvent evaporation.

Compound **4a**: 2-(Dibutylamino)-5,6-dimethylpyridine-3,4-dicarbonitrile. Yield 58%, yellow oil. IR (thin film, cm^−1^): 2233, 2208 (C≡N); 1580 (C=C). ^1^H NMR (500.13 MHz, DMSO-d_6_): δ 0.91 (t, *J* = 7.4 Hz, 6H, 2CH_2_CH_3_), 1.27–1.34 (m, 4H, 2CH_2_CH_3_), 1.54–1.60 (m, 4H, 2CH_2_CH_2_), 2.29 (s, 3H, CH_3_), 2.41 (s, 3H, CH_3_), 3.60 (t, *J* = 7.7 Hz, 4H, (CH_2_)_2_N). ^13^C NMR (125.76 MHz, DMSO-d_6_): δ 13.7, 16.0, 19.2, 23.7, 29.6, 49.3, 85.4, 114.9, 117.0, 122.8, 125.6, 154.9, 162.6. MS (EI, 70 eV): *m*/*z* (%) 284 ([M]^+^, 32), 241 ([M-C_3_H_7_]^+^, 56), 199 (100), 185 (73). Anal: Calcd for C_17_H_24_N_4_: C, 71.79; H, 8.51; N, 19.70. Found: C, 71.88; H, 8.47; N, 19.64.

Compound **4b**: 2-(Dibutylamino)-5,6,7,8-tetrahydroquinoline-3,4-dicarbonitrile. Yield 43%, mp 42–43 °C. IR (mineral oil, cm^−1^): 2232, 2203 (C≡N); 1572 (C=C). ^1^H NMR (500.13 MHz, DMSO-d_6_): 0.90 (t, *J* = 7.4 Hz, 6H, 2CH_2_CH_3_), 1.26–1.33 (m, 4H, 2CH_2_CH_3_), 1.53–1.59 (m, 4H, 2CH_2_), 1.72–1.80 (m, 4H, 2CH_2_), 2.67–2.74 (m, 4H, 2CH_2_), 3.57 (t, *J* = 7.7 Hz, 4H, (CH_2_)_2_N). ^13^C NMR (125.76 MHz, DMSO-d_6_): δ 13.7, 19.2, 21.6, 21.6, 26.0, 29.6, 32.9, 49.3, 86.5, 114.2, 116.8, 123.2, 126.0, 154.7, 162.5. MS (EI, 70 eV): *m*/*z* (%) 310 ([M]^+^, 36), 267 ([M-C_3_H_7_]^+^, 66), 225 (100), 211 (74). Anal: Calcd for C_19_H_26_N_4_: C, 73.51; H, 8.44; N, 18.05. Found: C, 73.22; H, 8.49; N, 17.94.

Compound **4c**: 2-(Dibutylamino)-6-phenylpyridine-3,4-dicarbonitrile. Yield 59%, mp 75–76 °C. IR (mineral oil, cm^−1^): 2240, 2202 (C≡N); 1587 (C=C). ^1^H NMR (500.13 MHz, DMSO-d_6_): δ 0.92 (t, *J* = 7.4 Hz, 6H, 2CH_2_CH_3_), 1.31–1.38 (m, 4H, 2CH_2_CH_3_), 1.60–1.66 (m, 4H, 2CH_2_), 3.65 (t, *J* = 7.6 Hz, 4H, (CH_2_)_2_N), 7.48–7.53 (m, 3H, Ph), 7.83 (s, 1H, CH_pyr_), 8.07–8.11 (m, 2H, Ph). ^13^C NMR (125.76 MHz, DMSO-d_6_): δ 13.7, 19.3, 29.6, 49.7, 86.9, 111.2, 115.5, 116.6, 127.2, 127.8, 128.9, 131.2, 136.0, 156.0, 158.6. MS (EI, 70 eV): *m*/*z* (%) 332 ([M]^+^, 51), 289 ([M-C_3_H_7_]^+^, 54), 247 (100), 233 (76). Anal: Calcd for C_21_H_24_N_4_: C, 75.87; H, 7.28; N, 16.85. Found: C, 75.51; H, 7.31; N, 16.80.

Compound **4d**: 2-(Dibutylamino)-5-methyl-6-phenylpyridine-3,4-dicarbonitrile. Yield 77%, mp 77–78 °C. IR (mineral oil, cm^−1^): 2229, 2203 (C≡N); 1583 (C=C). ^1^H NMR (500.13 MHz, DMSO-d_6_): δ 0.88 (t, *J* = 7.3 Hz, 6H, 2CH_2_CH_3_), 1.26–1.34 (m, 4H, 2CH_2_CH_3_), 1.57–1.64 (m, 4H, 2CH_2_), 2.34 (s, 3H, CH_3_), 3.61 (t, *J* = 7.6 Hz, 4H, (CH_2_)_2_N), 7.48–7.51 (m, 3H, Ph), 7.56–7.59 (m, 2H, Ph). ^13^C NMR (125.76 MHz, DMSO-d_6_): δ 13.7, 17.4, 19.3, 29.6, 49.5, 87.3, 114.9, 116.8, 121.9, 128.0, 128.2, 128.8, 129.5, 138.2, 154.7, 161.3. MS (EI, 70 eV): *m*/*z* (%) 346 ([M]^+^, 50), 303 ([M-C_3_H_7_]^+^, 47), 261 (100), 247 (77). Anal: Calcd for C_22_H_26_N_4_: C, 76.27; H, 7.56; N, 16.17. Found: C, 76.44; H, 7.53; N, 16.08.

Compound **4e**: 2-(Dibutylamino)-6-(4-methoxyphenyl)-5-methylpyridine-3,4-dicarbonitrile. Yield 61%, mp 76–77 °C. IR (mineral oil, cm^−1^): 2229, 2207 (C≡N); 1580 (C=C). ^1^H NMR (500.13 MHz, DMSO-d_6_): 0.90 (t, *J* = 7.4 Hz, 6H, 2CH_2_CH_3_), 1.26–1.36 (m, 4H, 2CH_2_CH_3_), 1.57–1.64 (m, 4H, 2CH_2_), 2.38 (s, 3H, CH_3_), 3.57–3.64 (m, 4H, (CH_2_)_2_N), 3.82 (s, 3H, OCH_3_), 7.02–7.06 (m, 2H, C_6_H_4_), 7.56–7.60 (m, 2H, C_6_H_4_).^13^C NMR (125.76 MHz, DMSO-d_6_): δ 13.7, 17.7, 19.3, 29.7, 49.5, 55.3, 86.5, 113.6, 115.0, 116.9, 121.4, 127.9, 130.4, 130.7, 154.6, 160.3, 160.8. MS (EI, 70 eV): *m*/*z* (%) 376 ([M]^+^, 46), 333 ([M-C_3_H_7_]^+^, 40), 291 (100), 277 (68). Anal: Calcd for C_23_H_28_N_4_O: C, 73.37; H, 7.50; N, 14.88. Found: C, 73.11; H, 7.54; N, 14.79.

Compound **4f**: 2-(Dibutylamino)-5,6-dihydrobenzo[*h*]quinoline-3,4-dicarbonitrile. Yield 64%, mp 89–90 °C. IR (mineral oil, cm^−1^): 2238, 2201 (C≡N); 1584 (C=C). ^1^H NMR (500.13 MHz, DMSO-d_6_): δ 0.93 (t, *J* = 7.4 Hz, 6H, 2CH_2_CH_3_), 1.31–1.41 (m, 4H, 2CH_2_CH_3_), 1.60–1.68 (m, 4H, 2CH_2_), 2.92–2.98 (m, 4H, 2CH_2_), 3.64–3.71 (m, 4H, (CH_2_)_2_N), 7.32–7.48 (m, 3H, C_6_H_4_), 8.07 (m, 1H, C_6_H_4_). ^13^C NMR (125.76 MHz, DMSO-d_6_): δ 13.8, 19.4, 24.9, 26.5, 29.7, 49.8, 86.7, 114.4, 117.0, 122.9, 125.3, 125.4, 127.3, 128.4, 131.4, 131.9, 139.7, 154.6, 155.5. MS (EI, 70 eV): *m*/*z* (%) 358 ([M]^+^, 58), 315 ([M-C_3_H_7_]^+^, 65), 273 (100), 259 (76). Anal: Calcd for C_23_H_26_N_4_: C, 77.06; H, 7.31; N, 15.63. Found: C, 76.87; H, 7.35; N, 15.57.

### 4.4. General Procedure for the Preparation of 2-(Diisobutylamino)Pyridine-3,4-Dicarbonitriles 5

An appropriate 2-chloropyridine-3,4-dicarbonitrile **1** (0.01 mol) was suspended in *i-*PrOH (5 mL), and then *N,N*-diisobutylamine (1.42 g, 0.011 mol) and DIPEA (1.42 g, 0.011 mol) were added. The reaction mixture was stirred at 60–70 °C for 24 h. After reaction completion (TLC monitoring), the mixture was cooled, and the precipitated product was filtered off and washed with ice-cold water and *i-*PrOH. The resulting product **5** was crystallized from *i-*PrOH, and then dried in a vacuum desiccator over CaCl_2_.

Compound **5a**: 2-(Diisobutylamino)-6-phenylpyridine-3,4-dicarbonitrile. Yield 48%, mp 180–181 °C. IR (mineral oil, cm^−1^): 2242, 2215 (C≡N); 1586 (C=C). ^1^H NMR (500.13 MHz, DMSO-d_6_): δ 0.91 (d, *J* = 6.6 Hz, 12H, 4CH_3_), 2.06–2.16 (m, 2H, 2CH(CH_3_)_2_), 3.67 (d, *J* = 7.4 Hz, 4H, (CH_2_)_2_N), 7.51–7.57 (m, 3H, Ph), 7.93 (s, 1H, CH), 8.11–8.16 (m, 2H, Ph). ^13^C NMR (125.76 MHz, DMSO-d_6_): δ 19.5, 26.8, 58.4, 87.9, 111.6, 115.5, 116.8, 127.3, 127.9, 129.1, 131.3, 136.0, 156.4, 158.6. MS (EI, 70 eV): *m*/*z* (%) 332 ([M]^+^, 16), 289 ([M-C_3_H_7_]^+^, 77), 233 (100). Anal: Calcd for C_21_H_24_N_4_: C, 75.87; H, 7.28; N, 16.85. Found: C, 75.66; H, 7.32; N, 16.80.

Compound **5b**: 2-(Diisobutylamino)-5-methyl-6-phenylpyridine-3,4-dicarbonitrile. Yield 56%, mp 105–106 °C. IR (mineral oil, cm^−1^): 2207 (C≡N); 1582 (C=C). ^1^H NMR (500.13 MHz, DMSO-d_6_): δ 0.86 (d, *J* = 6.6 Hz, 12H, 4CH_3_), 1.99–2.10 (m, 2H, 2CH(CH_3_)_2_), 2.36 (s, 3H, CH_3_), 3.57 (d, *J* = 7.4 Hz, 4H, (CH_2_)_2_N), 7.49–7.53 (m, 3H, Ph), 7.57–7.62 (m, 2H, Ph). ^13^C NMR (125.76 MHz, DMSO-d_6_): δ 17.5, 19.5, 26.7, 58.0, 88.4, 114.8, 116.8, 122.0, 128.0, 128.3, 128.8, 129.5, 138.2, 155.0, 161.3. MS (EI, 70 eV): *m*/*z* (%) 346 ([M]^+^, 14), 303 ([M-C_3_H_7_]^+^, 66), 247 (100). Anal: Calcd for C_22_H_26_N_4_: C, 76.27; H, 7.56; N, 16.17. Found: C, 76.04; H, 7.58; N, 16.09.

### 4.5. General Procedure for the Preparation of 2-(Butylamino)-5-Methyl-6-Phenylpyridine-3,4-Dicarbonitriles 6

The compound 2-(Butylamino)-5-methyl-6-phenylpyridine-3,4-dicarbonitrile **2d** (2.9 g, 0.01 mol) was dissolved in dry DMF (10 mL), and then NaH (0.52 g, 0.013 mol, 60% in mineral oil) and bromoethane (1.64 g, 0.015 mol, for **6a**) or bromopropane (1.85 g, 0.015 mol, for **6b**) were added. The reaction mixture was stirred at room temperature for 24 h. After reaction completion (TLC monitoring), the mixture was diluted with water (50 mL) and neutralized by acetic acid. The precipitated solid was filtered off, washed with ice-cold water, and crystallized from *i-*PrOH, and then dried in a vacuum desiccator over CaCl_2_.

Compound **6a**: 2-(Butyl(ethyl)amino)-5-methyl-6-phenylpyridine-3,4-dicarbonitrile. Yield 36%, mp 116–117 °C. IR (mineral oil, cm^−1^): 2235, 2209 (C≡N); 1582 (C=C). ^1^H NMR (500.13 MHz, DMSO-d_6_): δ 0.90 (t, *J* = 7.4 Hz, 3H, CH_2_CH_3_), 1.21 (t, *J* = 6.9 Hz, 3H, CH_2_CH_3_), 1.29–1.36 (m, 2H, CH_2_CH_3_), 1.60–1.66 (m, 2H, CH_2_), 2.35 (s, 3H, CH_3_), 3.59–3.64 (m, 2H, CH_2_N), 3.69 (q, *J* = 7.0 Hz, 2H, CH_2_N), 7.49–7.53 (m, 3H, Ph), 7.57–7.61 (m, 2H, Ph). ^13^C NMR (125.76 MHz, DMSO-d_6_): δ 13.9, 14.3, 18.1, 20.0, 30.4, 45.1, 49.5, 87.9, 115.5, 117.4, 122.2, 128.6, 128.9, 129.4, 130.1, 138.8, 155.3, 162.0. MS (EI, 70 eV): *m*/*z* (%) 318 ([M]^+^, 40), 275 ([M-C_3_H_7_]^+^, 100), 261 (25), 247 (84). Anal: Calcd for C_20_H_22_N_4_: C, 75.44; H, 6.96; N, 17.60. Found: C, 75.21; H, 7.00; N, 17.55.

Compound **6b**: 2-(Butyl(propyl)amino)-5-methyl-6-phenylpyridine-3,4-dicarbonitrile. Yield 29%, mp 78–79 °C. IR (mineral oil, cm^−1^): 2231, 2203 (C≡N); 1581 (C=C). ^1^H NMR (500.13 MHz, DMSO-d_6_): δ 0.85-0.90 (m, 6H, 2CH_2_CH_3_), 1.25–1.35 (m, 2H, CH_2_CH_3_), 1.56–1.69 (m, 4H, 2CH_2_), 2.35 (s, 3H, CH_3_), 3.51–3.67 (m, 4H, 2CH_2_N), 7.48–7.52 (m, 3H, Ph), 7.55–7.60 (m, 2H, Ph). ^13^C NMR (125.76 MHz, DMSO-d_6_): δ 10.6, 13.7, 17.4, 19.3, 20.9, 29.6, 49.5, 51.2, 87.3, 114.8, 116.7, 121.6, 128.0, 128.2, 128.8, 129.4, 138.2, 154.7, 161.3. MS (EI, 70 eV): *m*/*z* (%) 332 ([M]^+^, 40), 289 ([M-C_3_H_7_]^+^, 59), 261 (55), 247 (100). Anal: Calcd for C_21_H_24_N_4_: C, 75.87; H, 7.28; N, 16.85. Found: C, 75.63; H, 7.30; N, 16.78.

## 5. Conclusions

Thus, we have developed a facile approach to the synthesis of novel 2-(butylamino)pyridine-3,4-dicarbonitrile derivatives and have investigated their absorption, fluorescence, and solvatochromic properties. The synthesized compounds showed a unique property to be efficiently fluorescent both in solution and in solid state (dual-state emission). The highest fluorescence of the synthesized compounds was observed in nonpolar media with a quantum yield up to 63%. The strongest photoluminescence was noted for the butylaminocinchomeronic dinitrile derivative. The introduction of an additional substituent to the amino nitrogen atom led to the decrease in emissions in a row of *N-*substituted methyl, ethyl, propyl and butyl derivatives. All the 2-(butylamino)pyridine-3,4-dicarbonitrile derivatives also showed solid-state emissions from the blue to green regions of the spectra 478–564 nm.

## Figures and Tables

**Table 1:** Structure of starting compounds 1 and isolated products 2–4.

Entry	Substrate 1	Product 2 ^a^	Product 3 ^b^	Product 4 ^c^
**a**	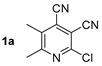	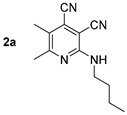	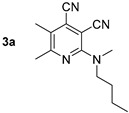	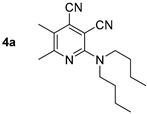
**b**	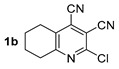	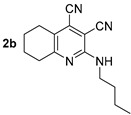	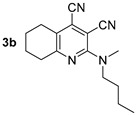	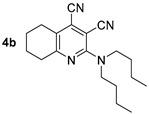
**c**	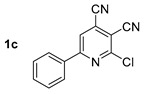	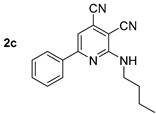	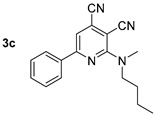	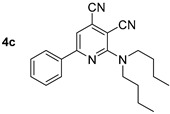
**d**	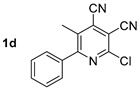	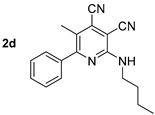	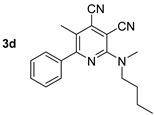	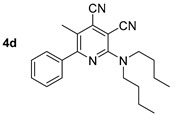
**e**	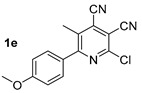	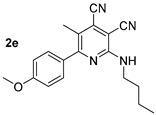	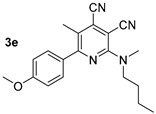	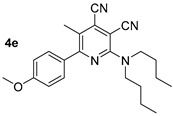
**f**	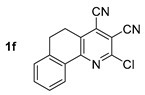	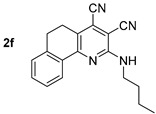	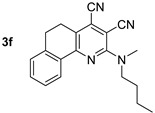	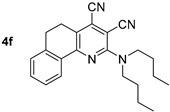

^a^ See Scheme 1 for details. ^b^ See Scheme 2 for details. ^c^ See Scheme 3 for details.

**Table 2:** Solvatochromic properties of the compound **2d.**

Solvent	λ_abs_, nm ^a^	ε, M^−1^ cm^−1^	λ_em_, nm ^b^	Δλ, nm (cm^−1^) ^c^	Φ_s_, % ^d^
PhMe	404	11288	442	38 (2128)	60
EtOAc	402	10766	456	54 (2946)	47
DCM	402	9794	452	50 (2752)	63
MeCN	403	7842	463	60 (3216)	51
DMSO	412	9462	477	65 (3307)	26
Acetone	404	9800	462	58 (3107)	49
EtOH	406	8986	470	64 (3354)	39
1,4-Dioxane	403	9646	454	51 (2787)	49
Pyridine	410	10834	468	58 (3023)	<1
AcOH	402	10754	467	65 (3462)	48

^a^ The absorption spectra were registered for 5 × 10^−5^ M solutions. ^b^ The photoluminescence spectra were registered for 5 × 10^−5^ M solutions (long-wavelength absorption maxima were used for excitation). ^c^ Stokes shift values were determined from long-wavelength absorption and emission maxima. ^d^ The relative photoluminescence quantum yields in solution (Φ_s_) were determined using quinine sulfate in 0.05 M sulfuric acid (Φ_std_ = 60 ± 2%); the excitation wavelength was 380 nm [46].

**Table 3:** Photophysical parameters of the synthesized 2-(butylamino)cinchomeronic dinitrile derivatives **2–6.**

Compound	λ_abs_, nm ^a^	ε, M^−1^ cm^−1^	λ_em_, nm ^b^	Δλ, nm (cm^−1^) ^c^	Φ_s_, % ^d^	λ_em_, nm ^e^	Φ_c_, % ^f^
**2a**	389	5720	429	40 (2397)	54	507	13
**2b**	396	5980	432	36 (2104)	60	547	<1
**2c**	403	7100	434	31 (1772)	63	474	16
**2d**	403	11,280	442	39 (2189)	60	483	72
**2e**	406	10,240	441	35 (1955)	61	496	38
**2f**	423	9920	452	29 (1517)	50	517	2
**3a**	395	4880	449	54 (3045)	36	478	14
**3b**	402	5020	455	53 (2898)	39	491, 520	6
**3c**	409	6600	458	49 (2616)	37	499	13
**3d**	410	6640	463	53 (2792)	41	499	11
**3e**	409	7130	461	52 (2758)	43	497	3
**3f**	439	8460	471	32 (1548)	47	540, 564	20
**4a**	398	4300	456	58 (3196)	33	–	–
**4b**	404	4680	457	53 (2871)	33	529	<1
**4c**	411	7400	459	48 (2544)	32	501	38
**4d**	413	6440	466	53 (2754)	37	519	17
**4e**	412	7620	462	50 (2627)	41	494	10
**4f**	426	8460	472	46 (2288)	43	532	7
**5a**	411	6560	464	53 (2779)	23	500	8
**5b**	413	6500	470	57 (2936)	33	507	20
**6a**	411	6060	464	53 (2779)	40	493	7
**6b**	412	6020	466	54 (2813)	38	497	2

^a^ The absorption spectra were registered for 5 × 10^−5^ M solutions. ^b^ The photoluminescence spectra were registered for 5 × 10^−5^ M solutions (long-wavelength absorption maxima were used for excitation). ^c^ Stokes shift values were determined from long-wavelength absorption and emission maxima. ^d^ The relative photoluminescence quantum yields in solution (Φ_s_) were determined using quinine sulfate in 0.05 M sulfuric acid (Φ_std_ = 60 ± 2%); the excitation wavelength was 380 nm [46]. ^e^ The solid-state photoluminescence spectra were registered in powder; the excitation wavelength was 365 nm. ^f^ The photoluminescence quantum yields in the crystalline state (Φ_c_) were determined at room temperature using 365 nm excitation.

**Table 4:** Structure of starting compounds 1 and isolated products 5 and 6.

Entry	Substrate 1	Product 5 ^a^	Entry	Substrate 2	Product 6 ^b^
1	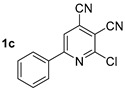	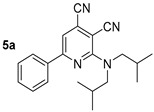	3	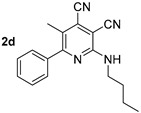	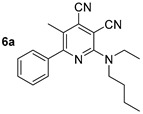
2	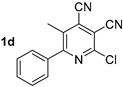	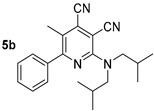	4	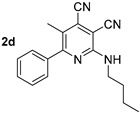	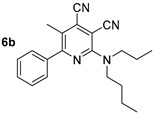

^a^ See Scheme 4 for details. ^b^ See Scheme 5 for details.

## Data Availability

Not applicable.

## Supplementary Materials

Copies of the NMR spectra for compounds **2—6** can be downloaded at: https://www.mdpi.com/article/10.3390/molecules27217144/s1.

## Sample Availability

Samples of all the reported compounds are available from the authors.
