# Supplementary material for: Dual-State Emission of 2-(Butylamino)Cinchomeronic Dinitrile Derivatives
Source: Molecules. 2022 Oct 22;27(21):7144. doi: 10.3390/molecules27217144 (PMC9653811; doi:10.3390/molecules27217144)
Supplement: Supplementary file 1 [file molecules-27-07144-s001.zip › molecules-1967073-supplementary1.pdf]

Supporting information

Dual-State Emission of 2-(Butylamino)cinchomeronic Dinitrile Derivatives

Konstantin V. Lipin, Mikhail Yu. Ievlev, Anastasiya I. Ershova, Oleg V. Ershov*

Department of organic and pharmaceutical chemistry, Chuvash State University named after I.N. Ulyanov, Moskovsky pr. 15, Cheboksary 428015, Russia

** Corresponding author. Tel.: +7-908-300-1643;*

E-mail address: oleg.ershov@mail.ru (O.V. Ershov)

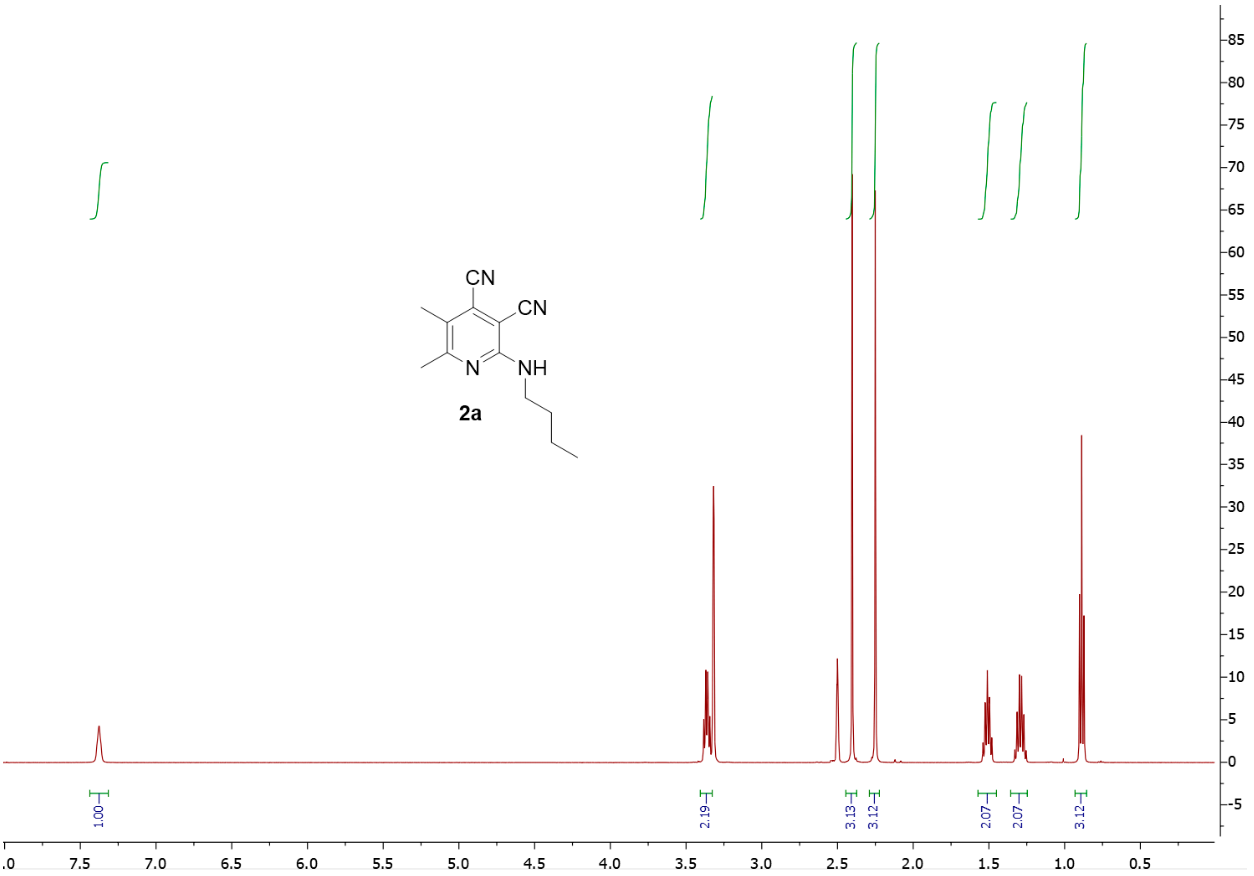

Figure S1. ^{1}H NMR-spectrum of **2a** (500.13 MHz, DMSO-d6, 298K).

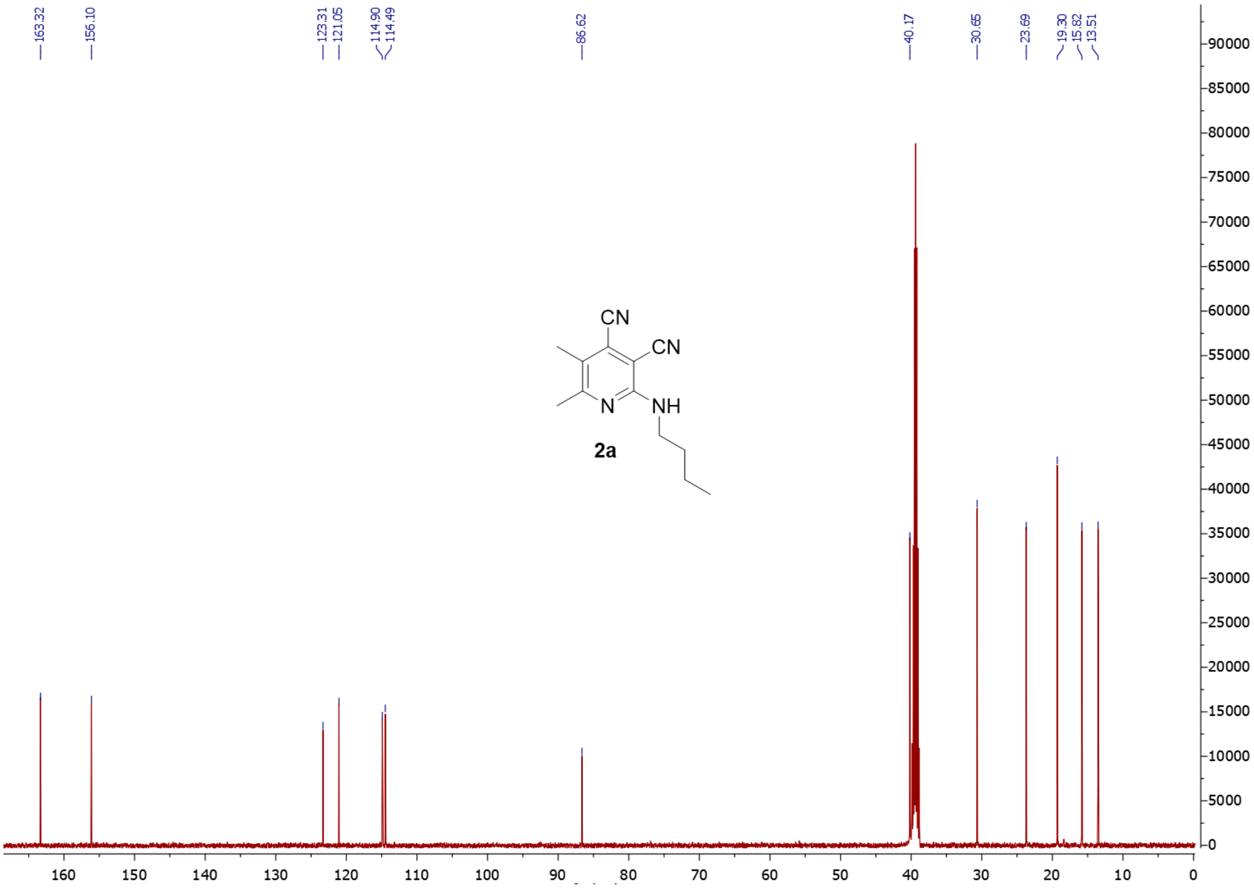

Figure S2. ^{13}C NMR-spectrum of **2a** (125.76 MHz, DMSO-d6, 299K)

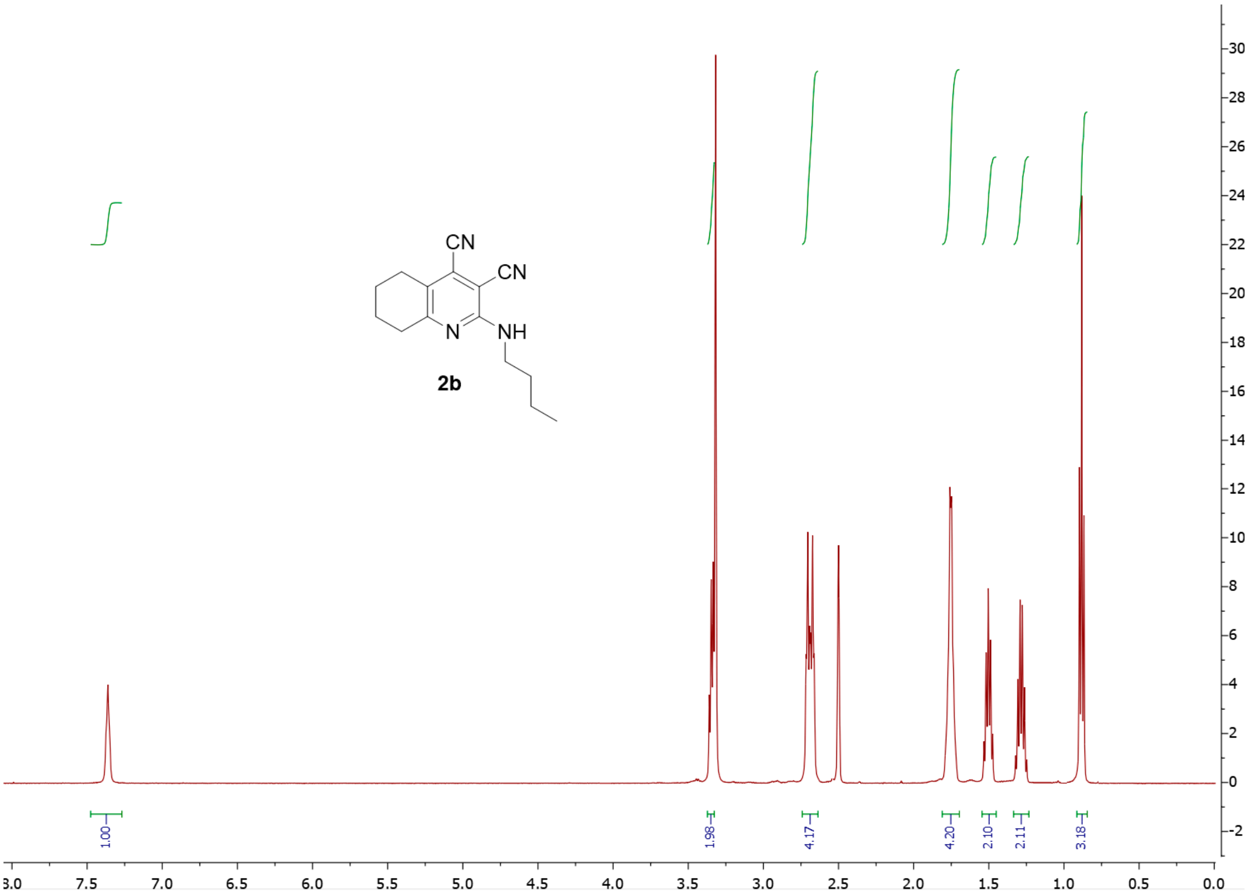

Figure S3. ^{1}H NMR-spectrum of **2b** (500.13 MHz, DMSO-d6, 298K)

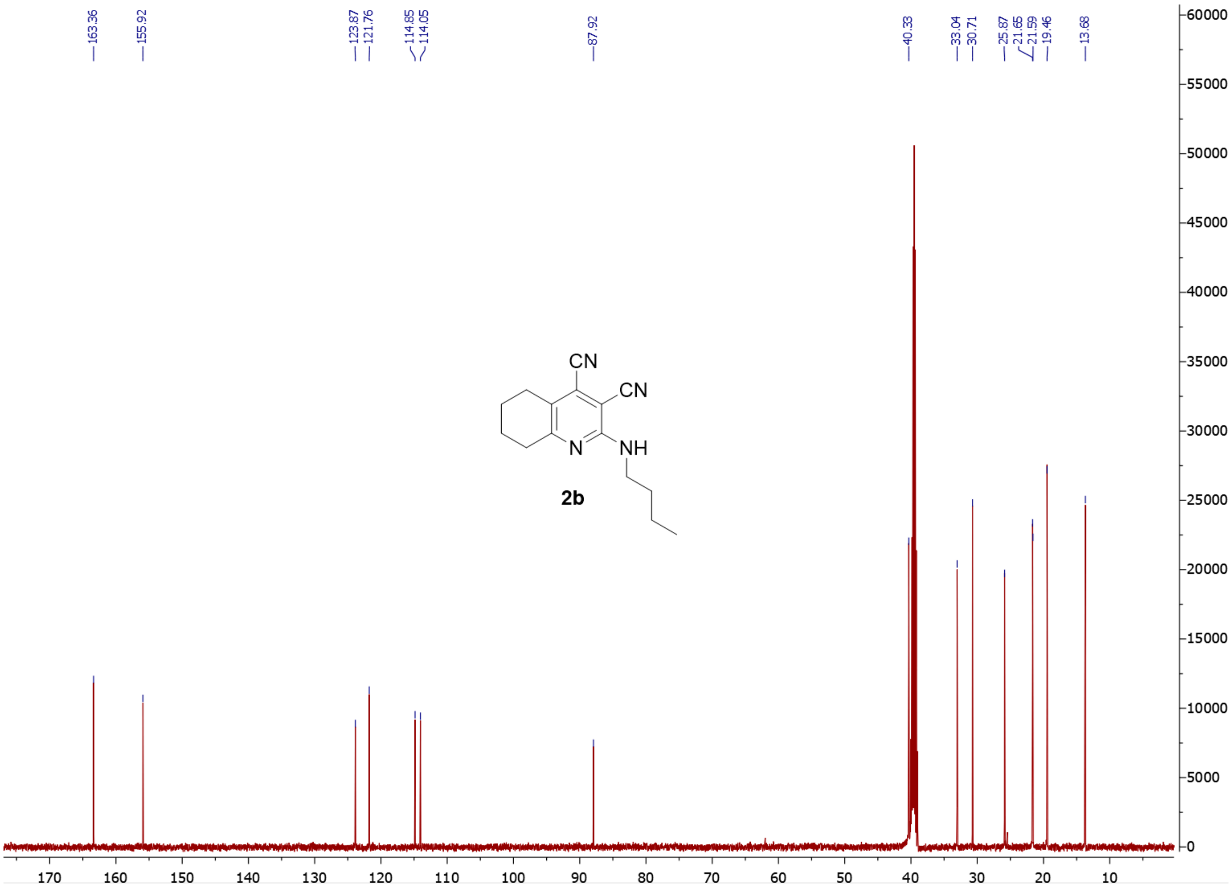

Figure S4. ^{13}C NMR-spectrum of **2b** (125.76 MHz, DMSO-d6, 299K)

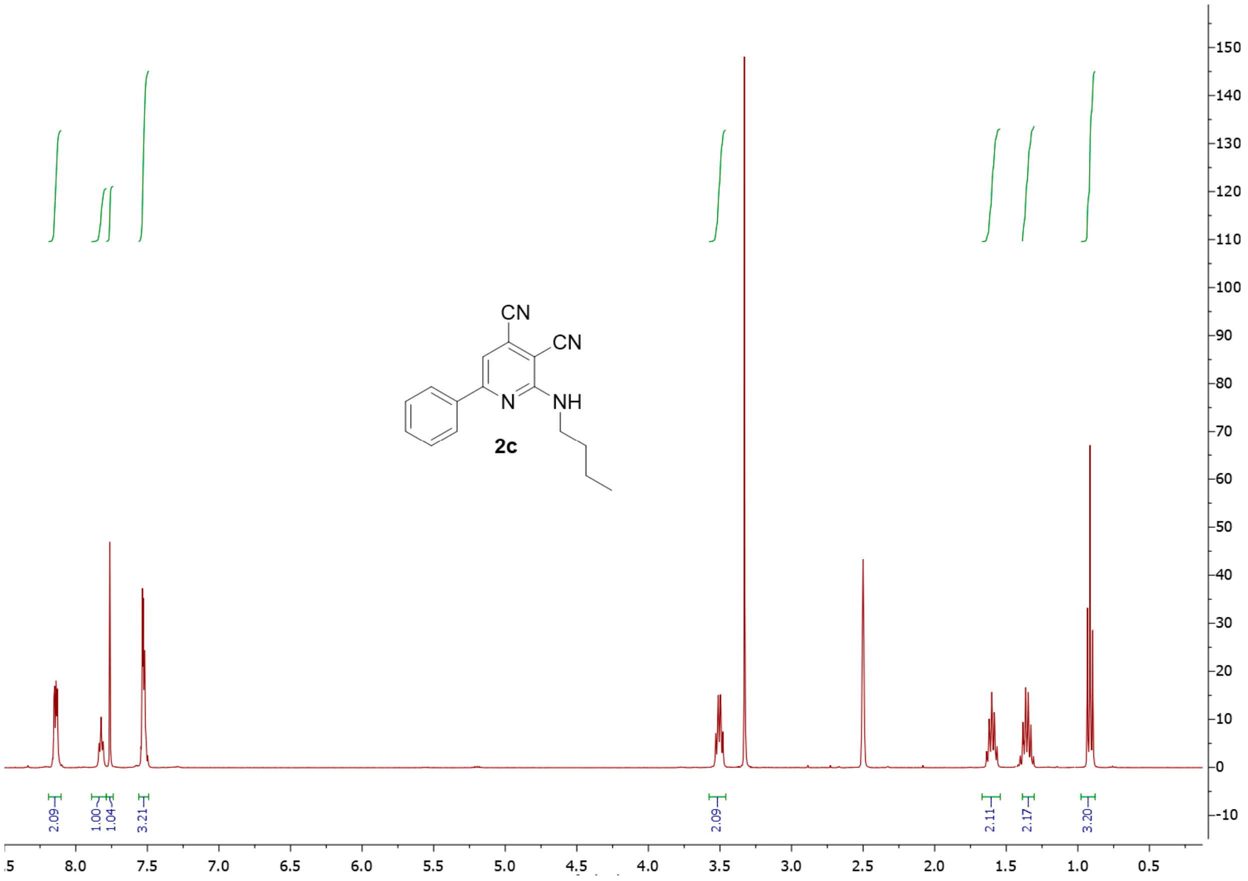

Figure S5. ^{1}H NMR-spectrum of **2c** (500.13 MHz, DMSO-d6, 298K)

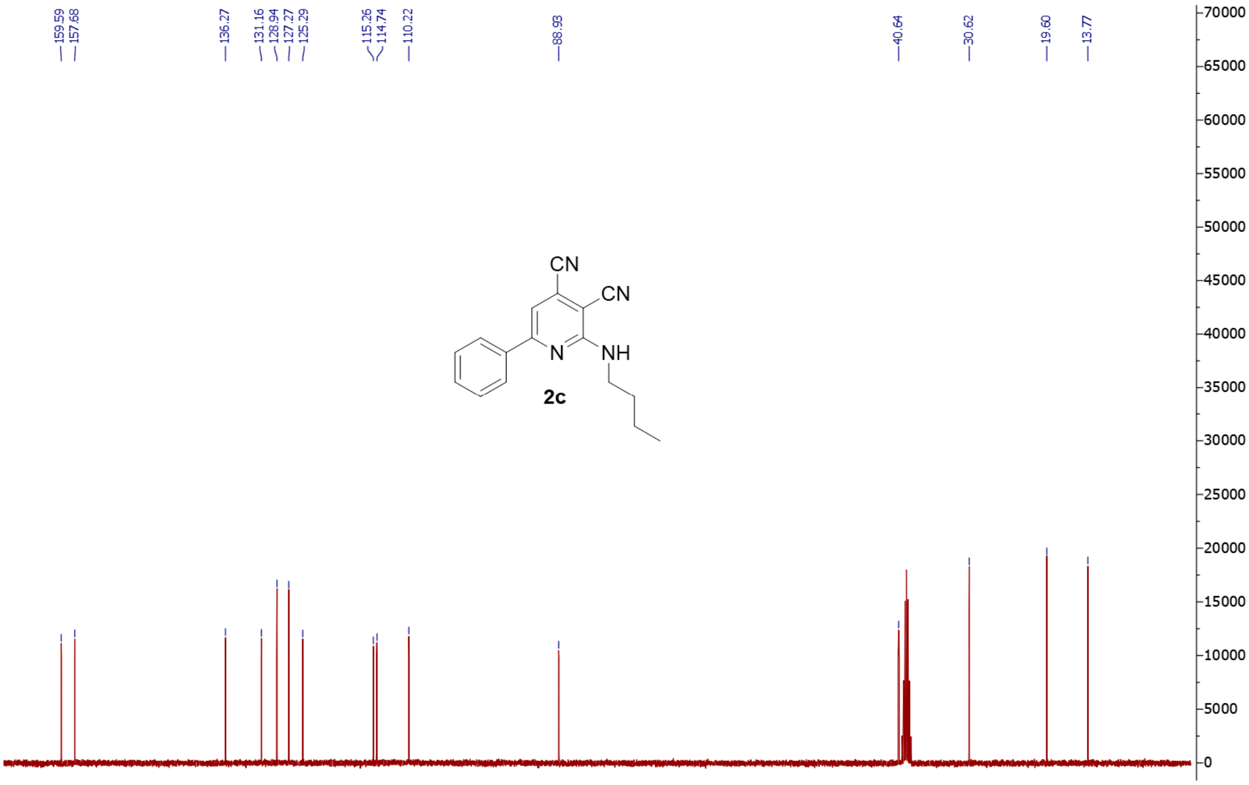

Figure S6. ^{13}C NMR-spectrum of **2c** (125.76 MHz, DMSO-d6, 299K)

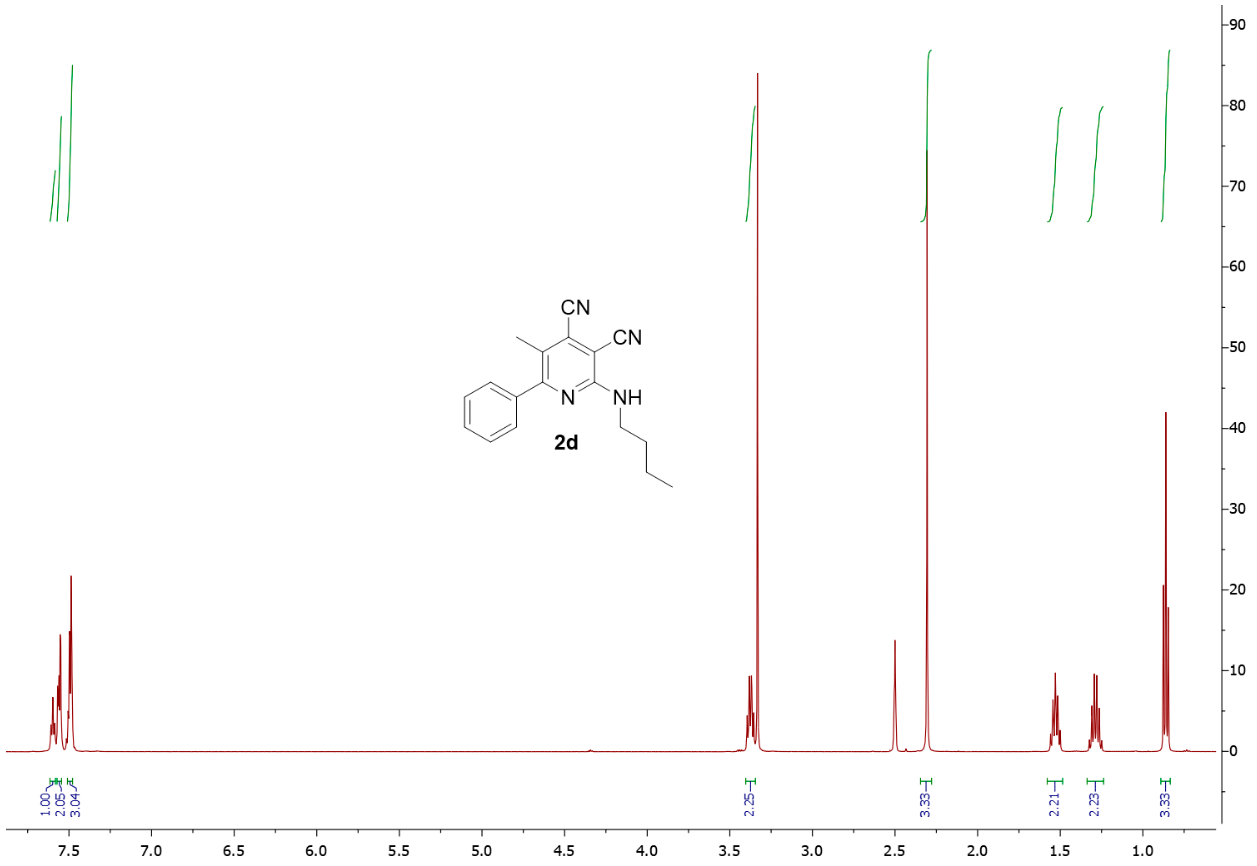

Figure S7. ^{1}H NMR-spectrum of **2d** (500.13 MHz, DMSO-d6, 298K)

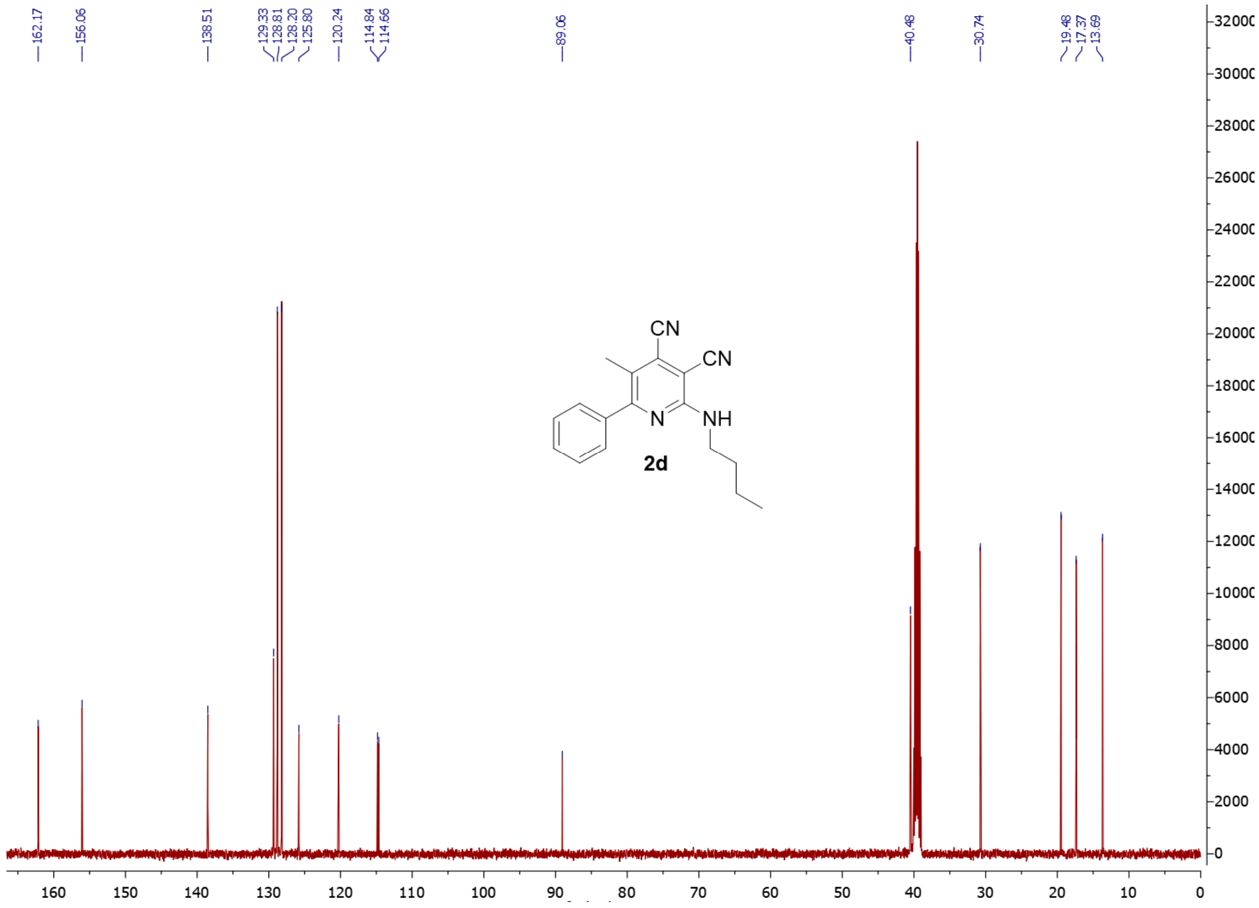

Figure S8. ^{13}C NMR-spectrum of **2d** (125.76 MHz, DMSO-d6, 299K)

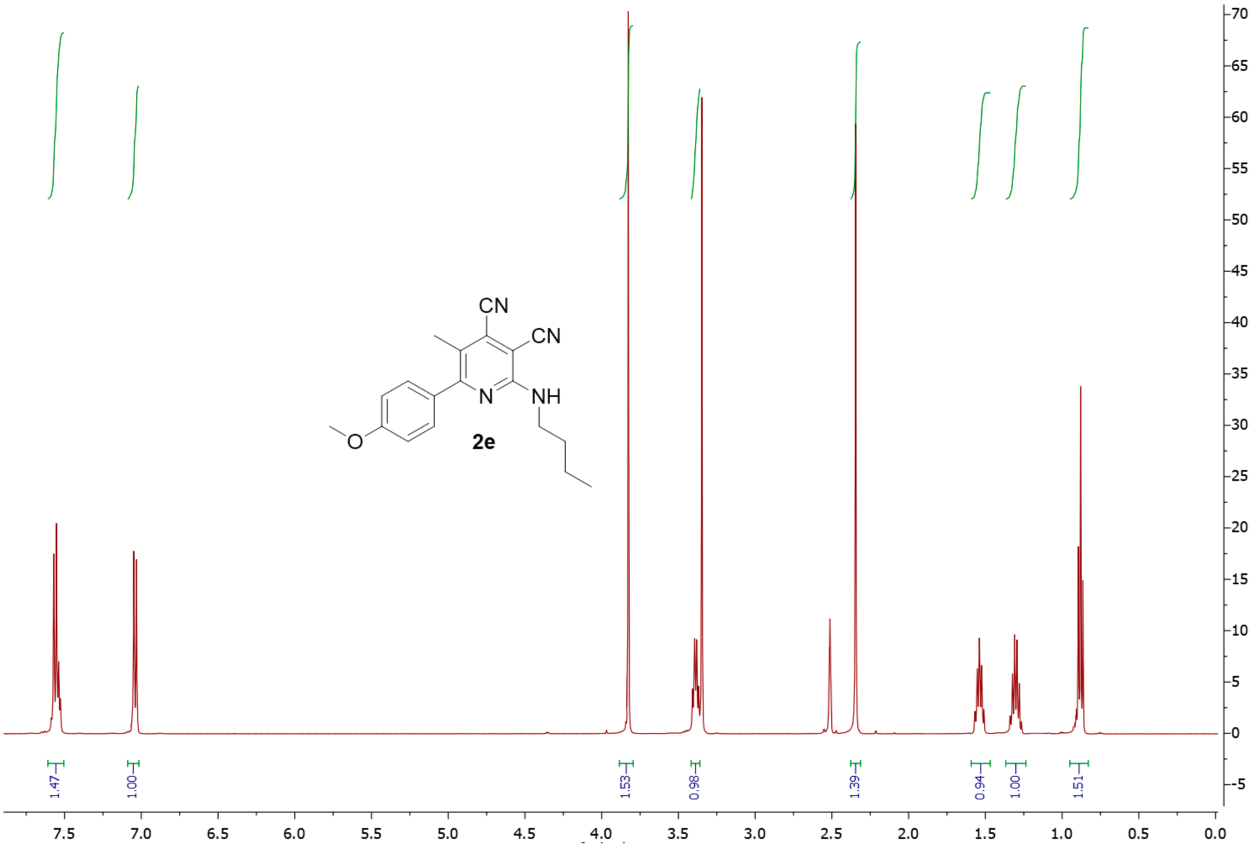

Figure S9. ^{1}H NMR-spectrum of **2e** (500.13 MHz, DMSO-d6, 298K)

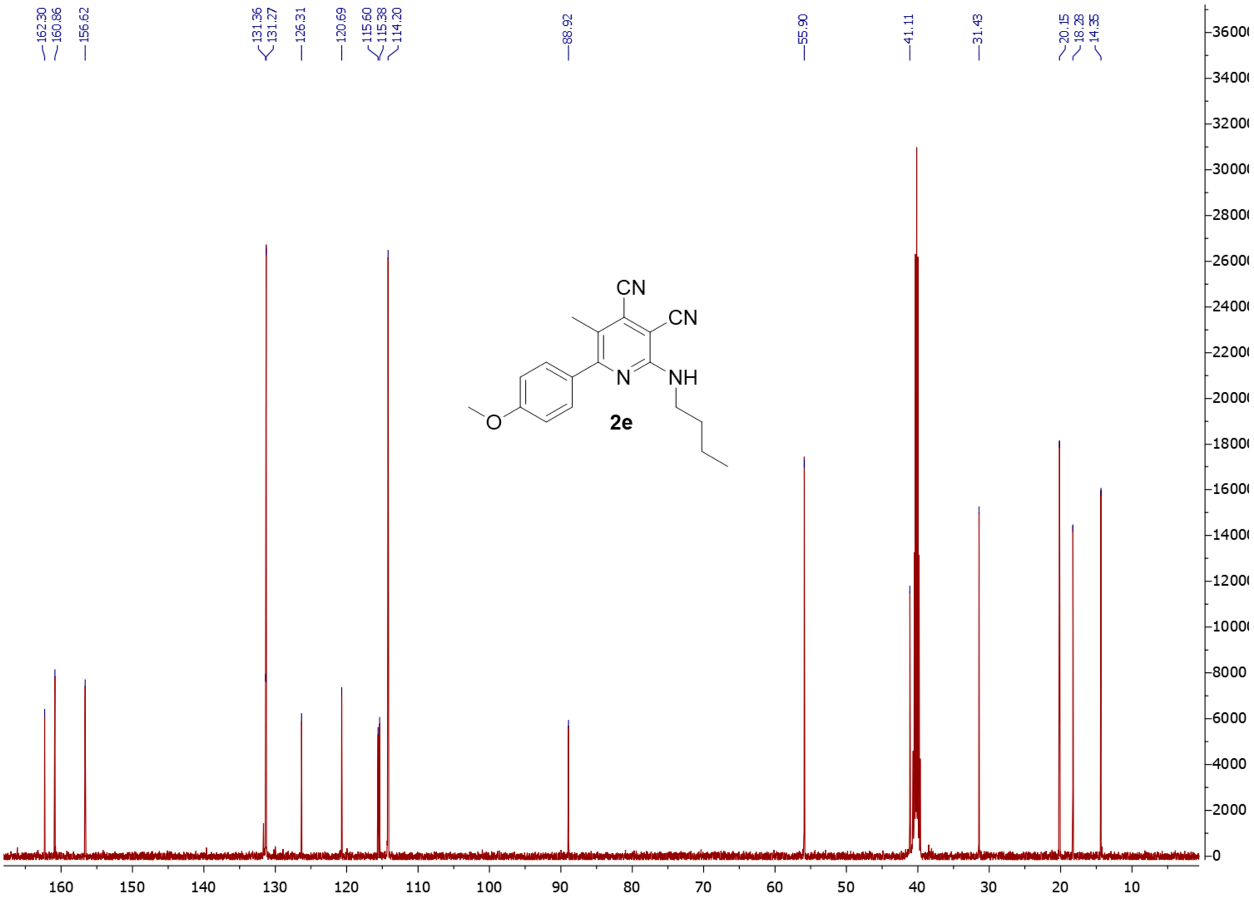

Figure S10. ^{13}C NMR-spectrum of **2e** (125.76 MHz, DMSO-d6, 299K)

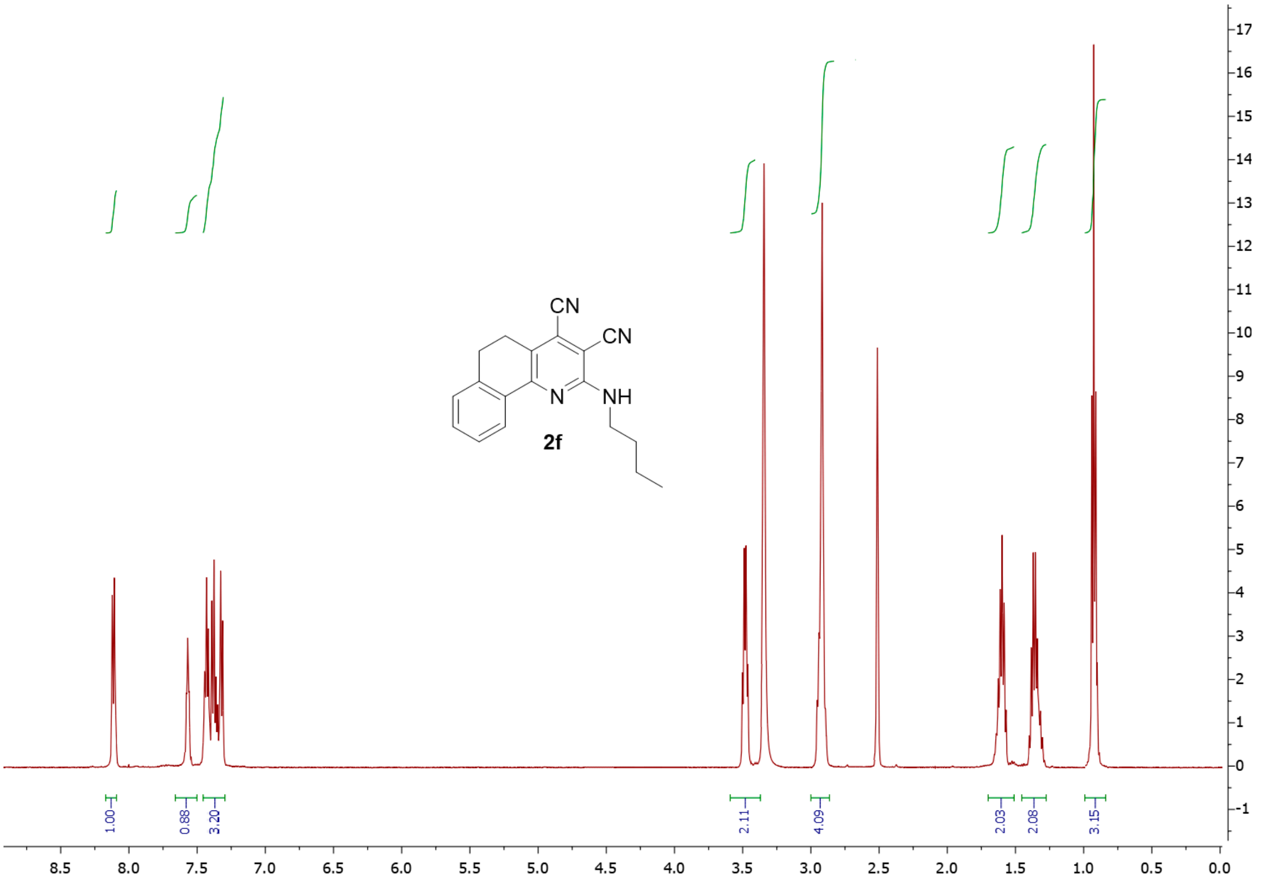

Figure S11. ^{1}H NMR-spectrum of **2f** (500.13 MHz, DMSO-d6, 298K)

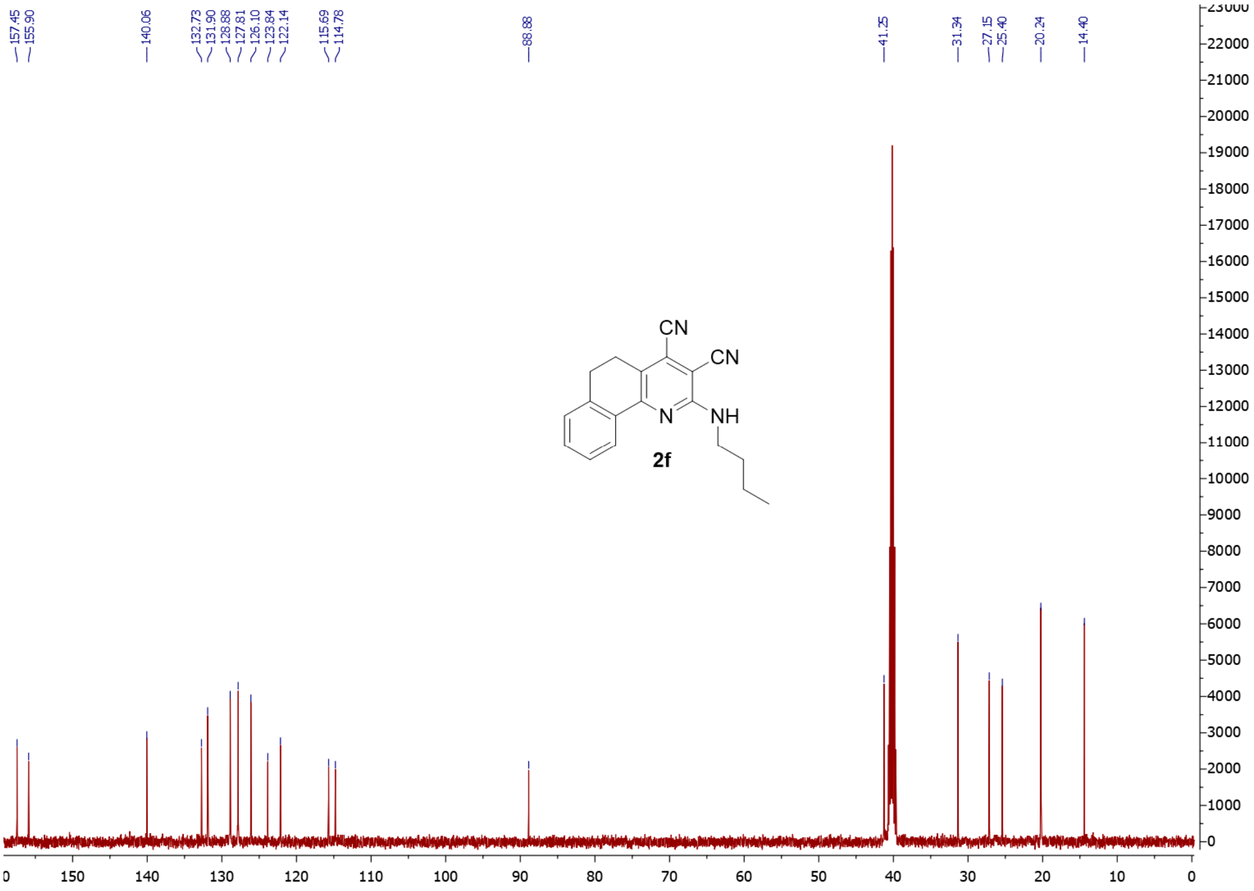

Figure S12. ^{13}C NMR-spectrum of **2f** (125.76 MHz, DMSO-d6, 299K)

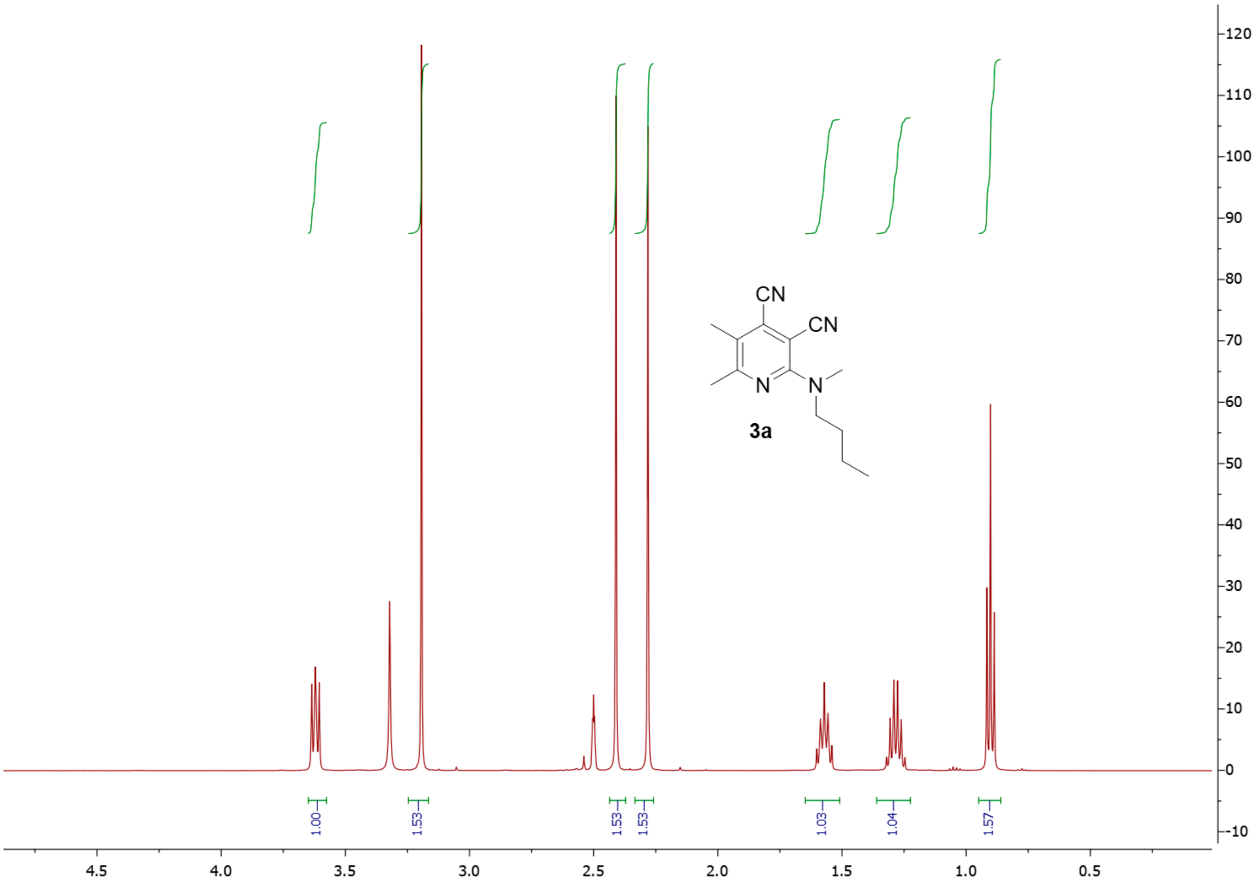

Figure S13. ^{1}H NMR-spectrum of **3a** (500.13 MHz, DMSO-d6, 298K)

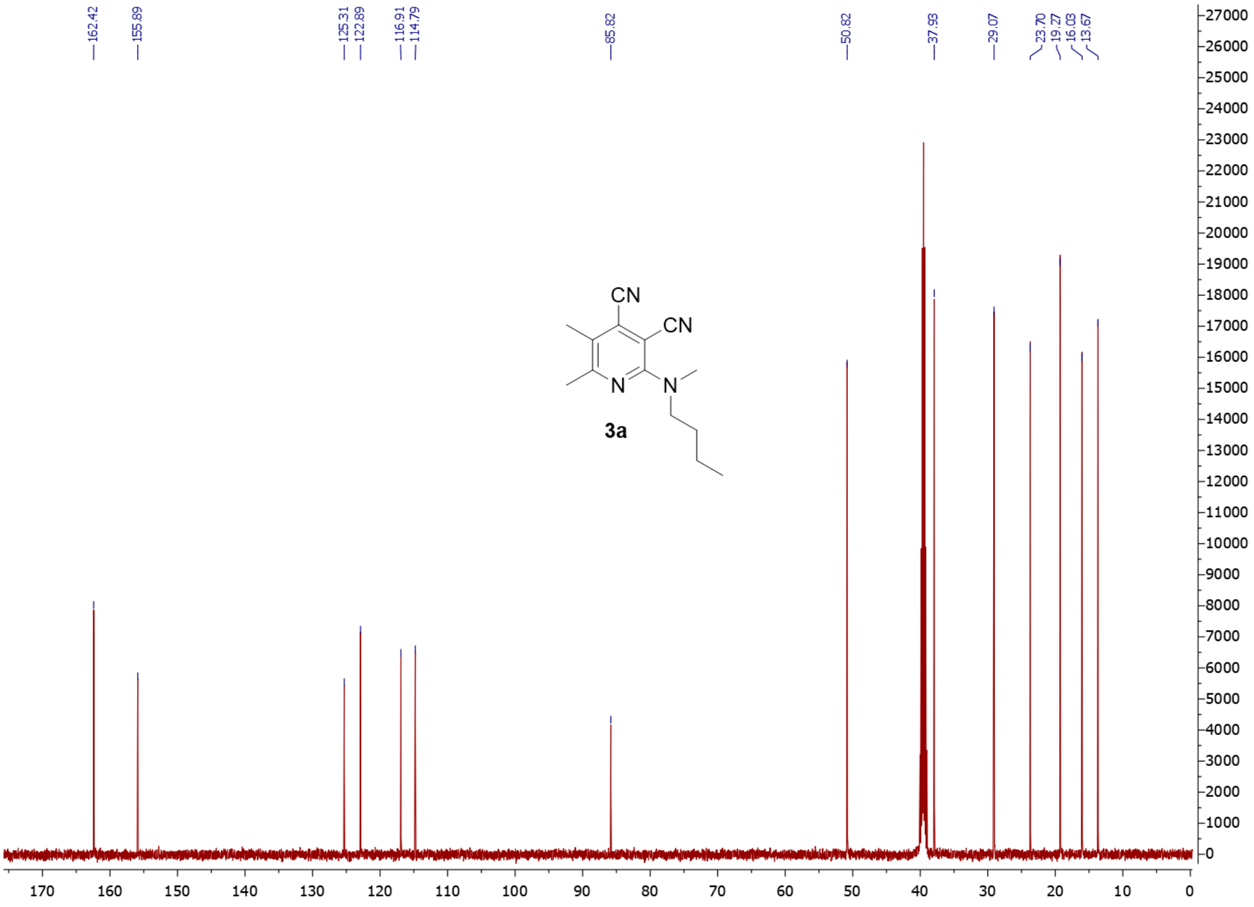

Figure S14. ^{13}C NMR-spectrum of **3a** (125.76 MHz, DMSO-d6, 299K)

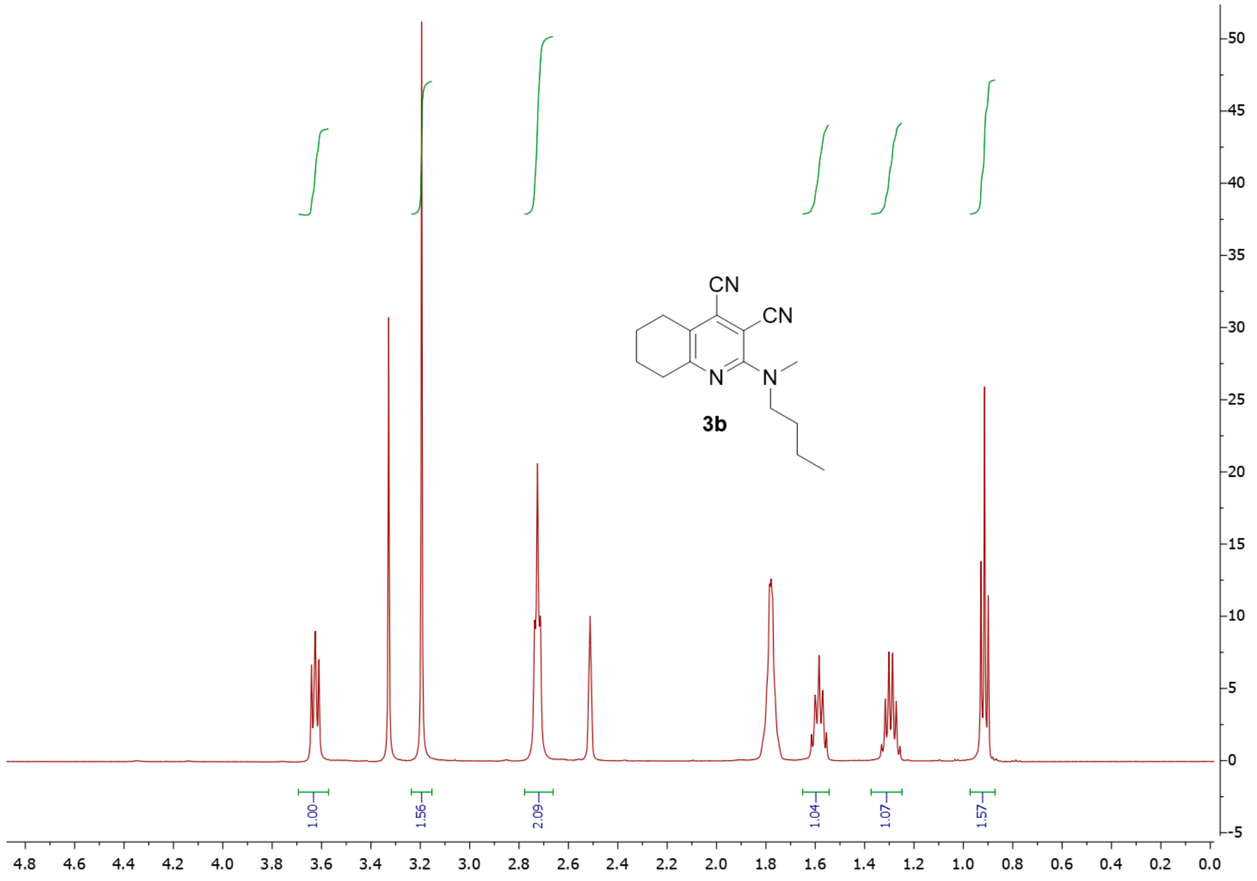

Figure S15. ^{1}H NMR-spectrum of **3b** (500.13 MHz, DMSO-d6, 298K)

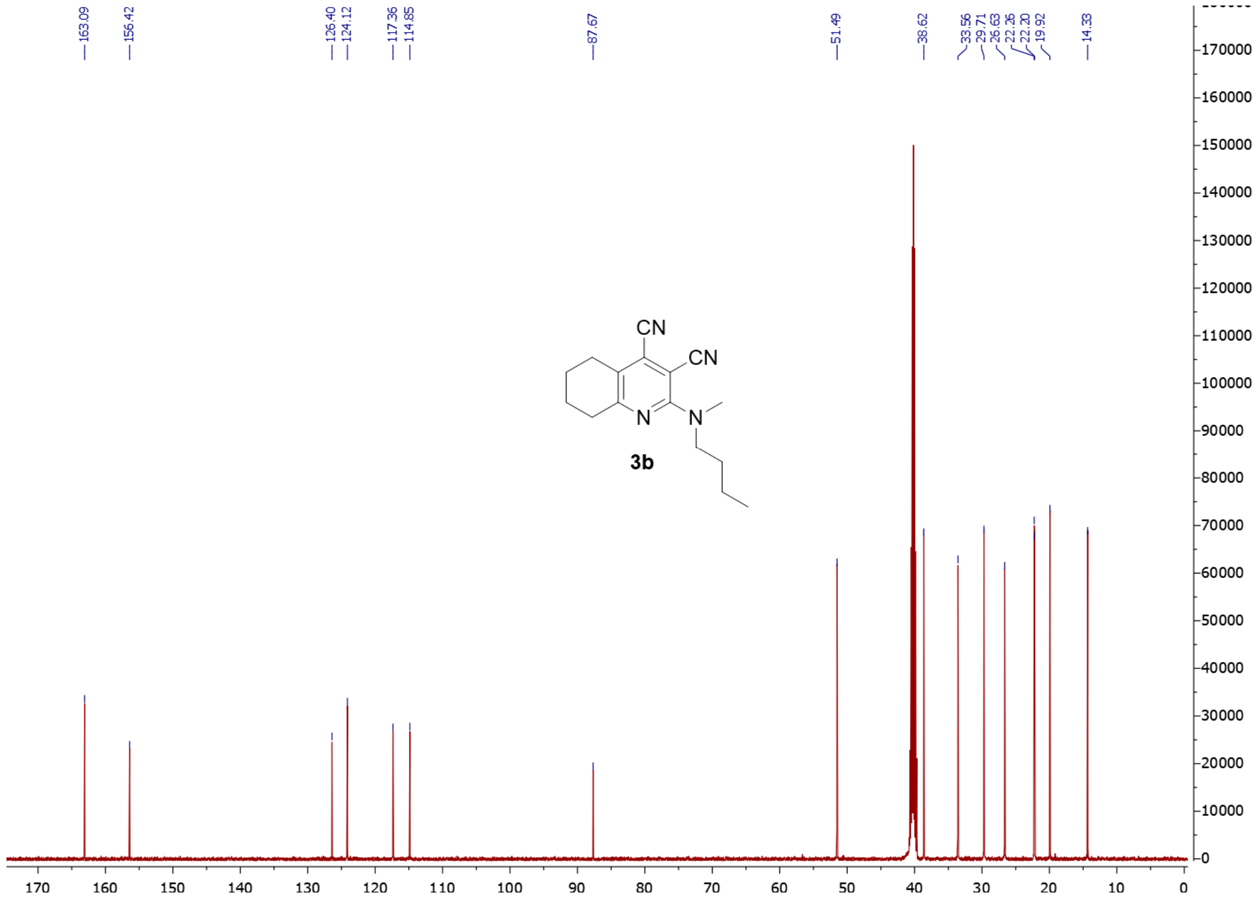

Figure S16. ^{13}C NMR-spectrum of **3b** (125.76 MHz, DMSO-d6, 299K)

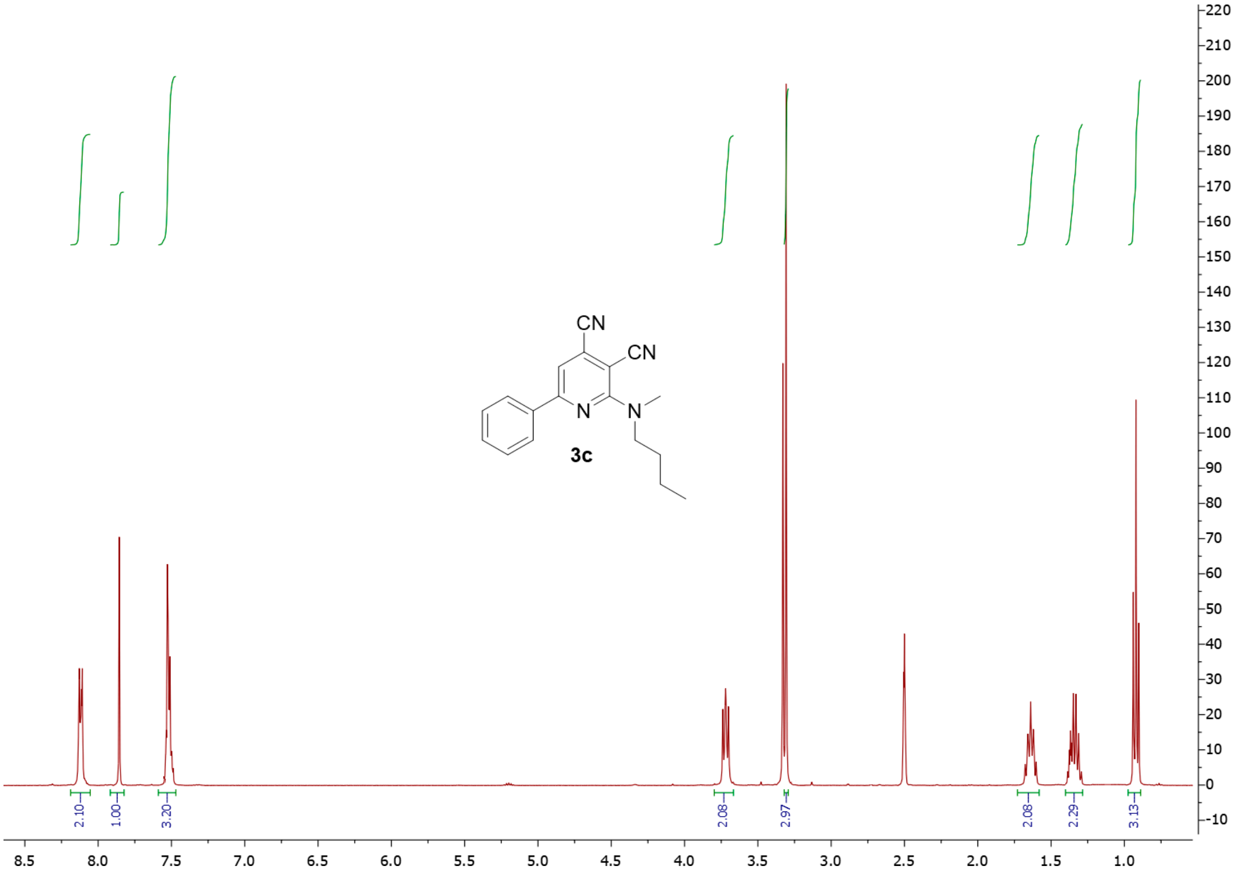

Figure S17. ^{1}H NMR-spectrum of **3c** (500.13 MHz, DMSO-d6, 298K)

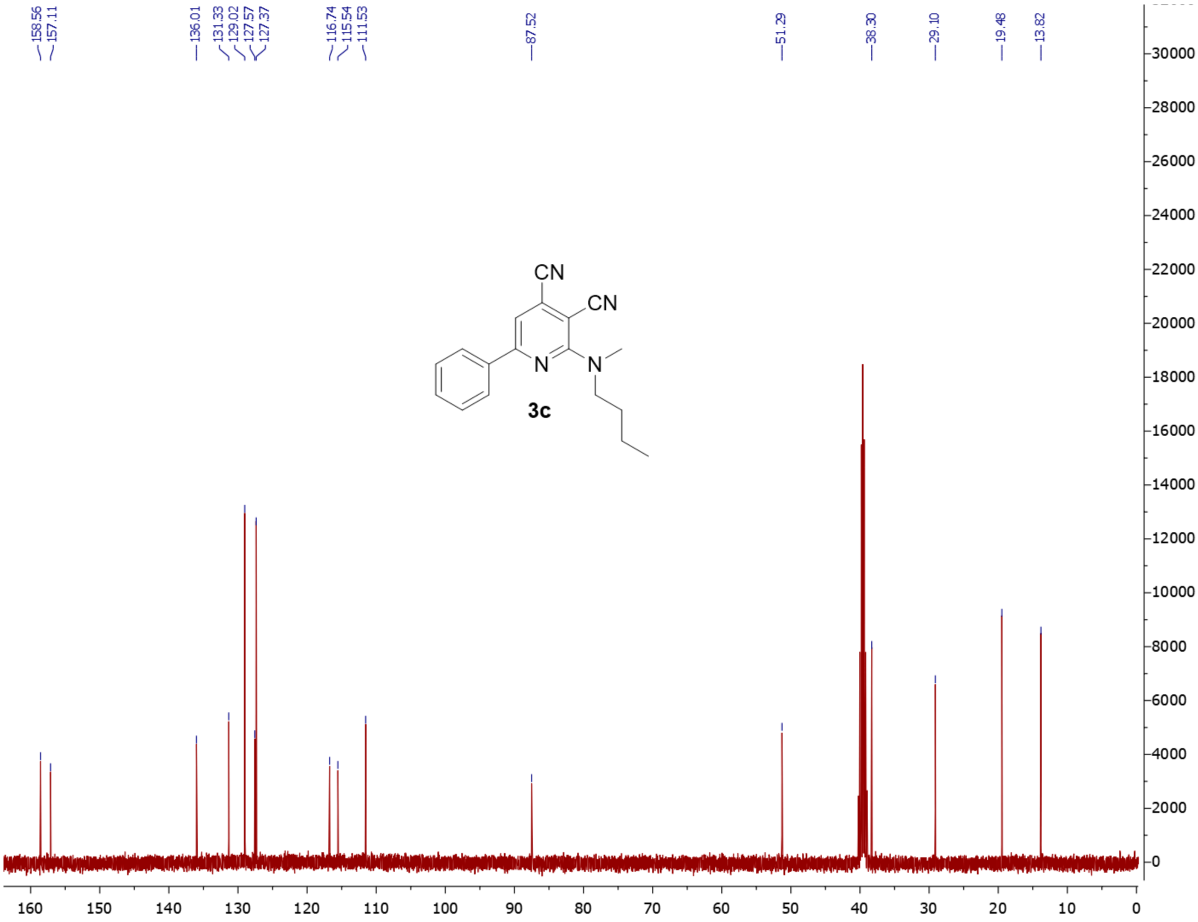

Figure S18. ^{13}C NMR-spectrum of **3c** (125.76 MHz, DMSO-d6, 299K)

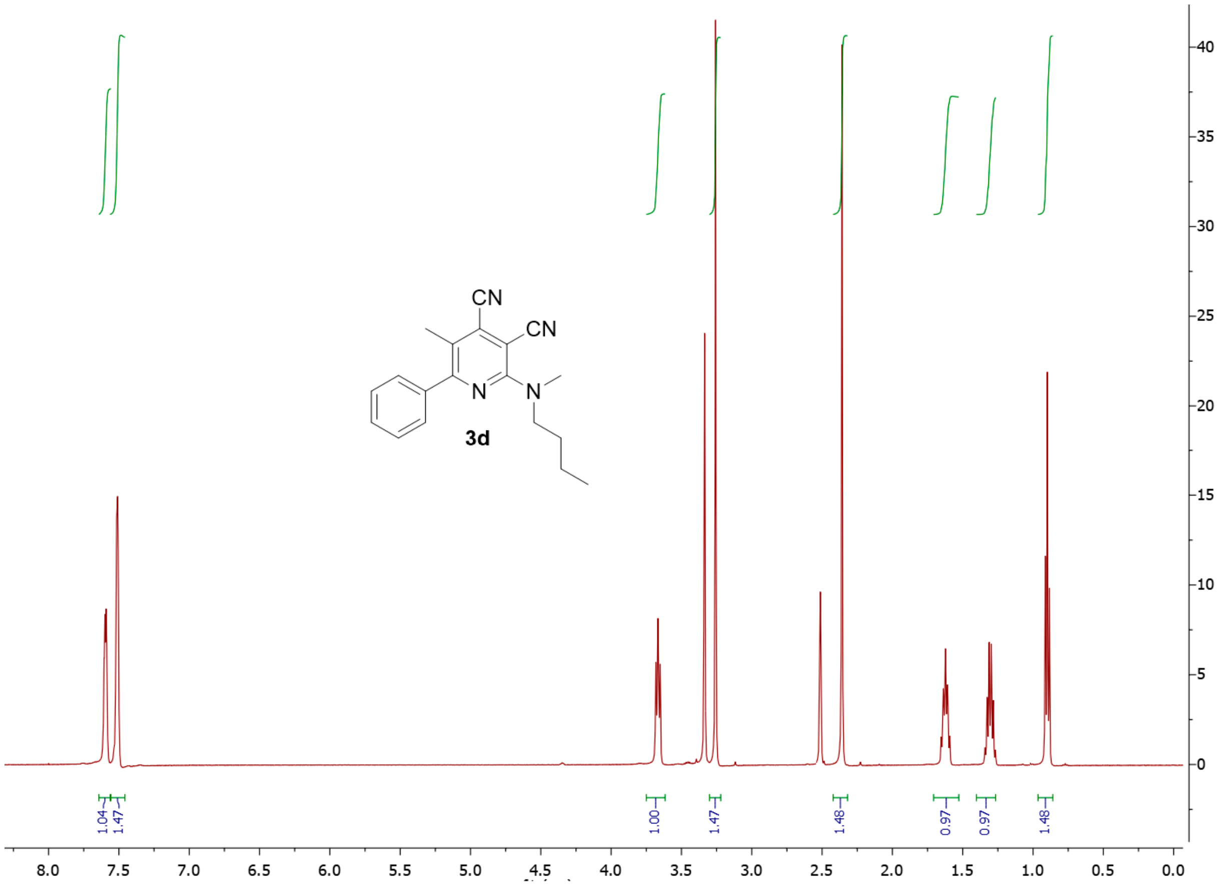

Figure S19. ^{1}H NMR-spectrum of **3d** (500.13 MHz, DMSO-d6, 298K)

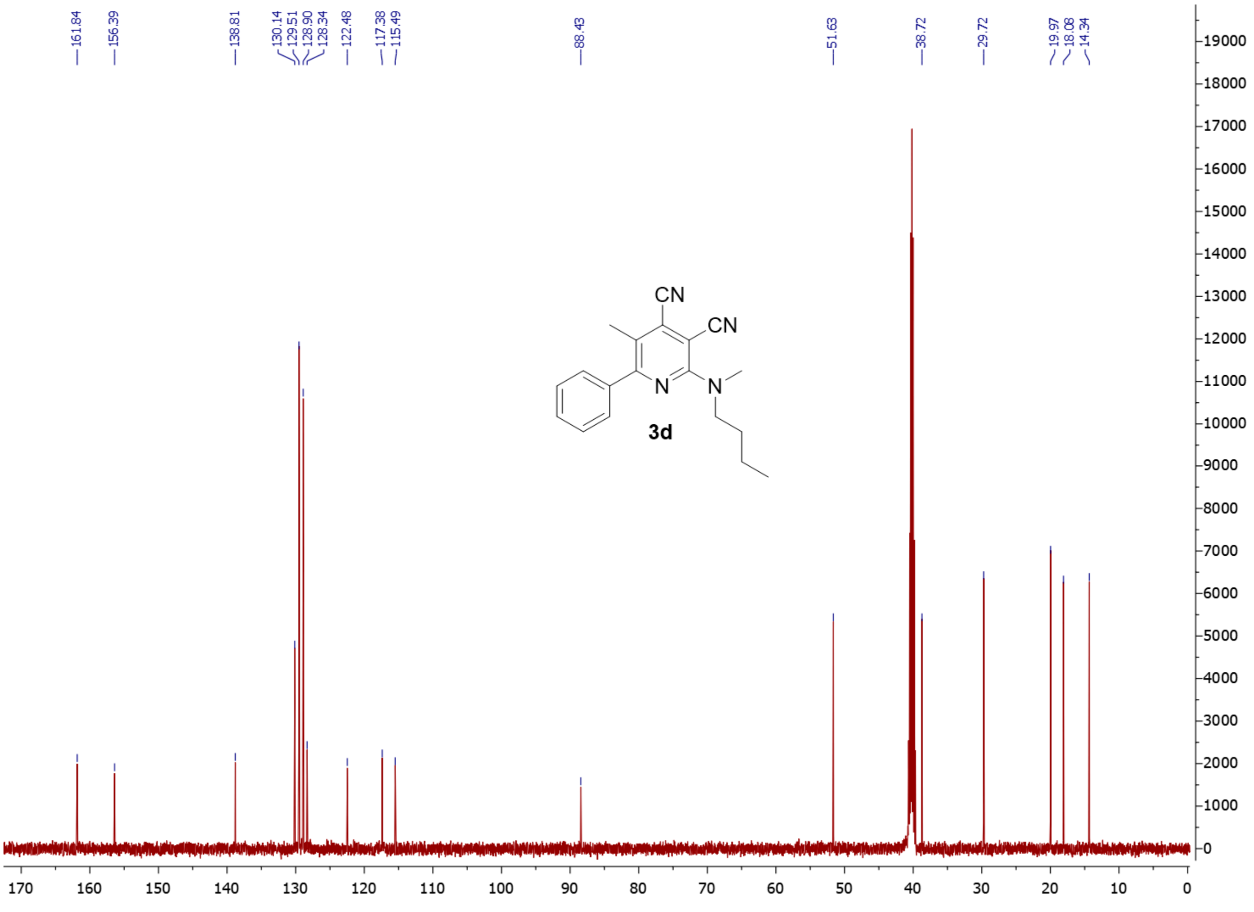

Figure S20. ^{13}C NMR-spectrum of **3d** (125.76 MHz, DMSO-d6, 299K)

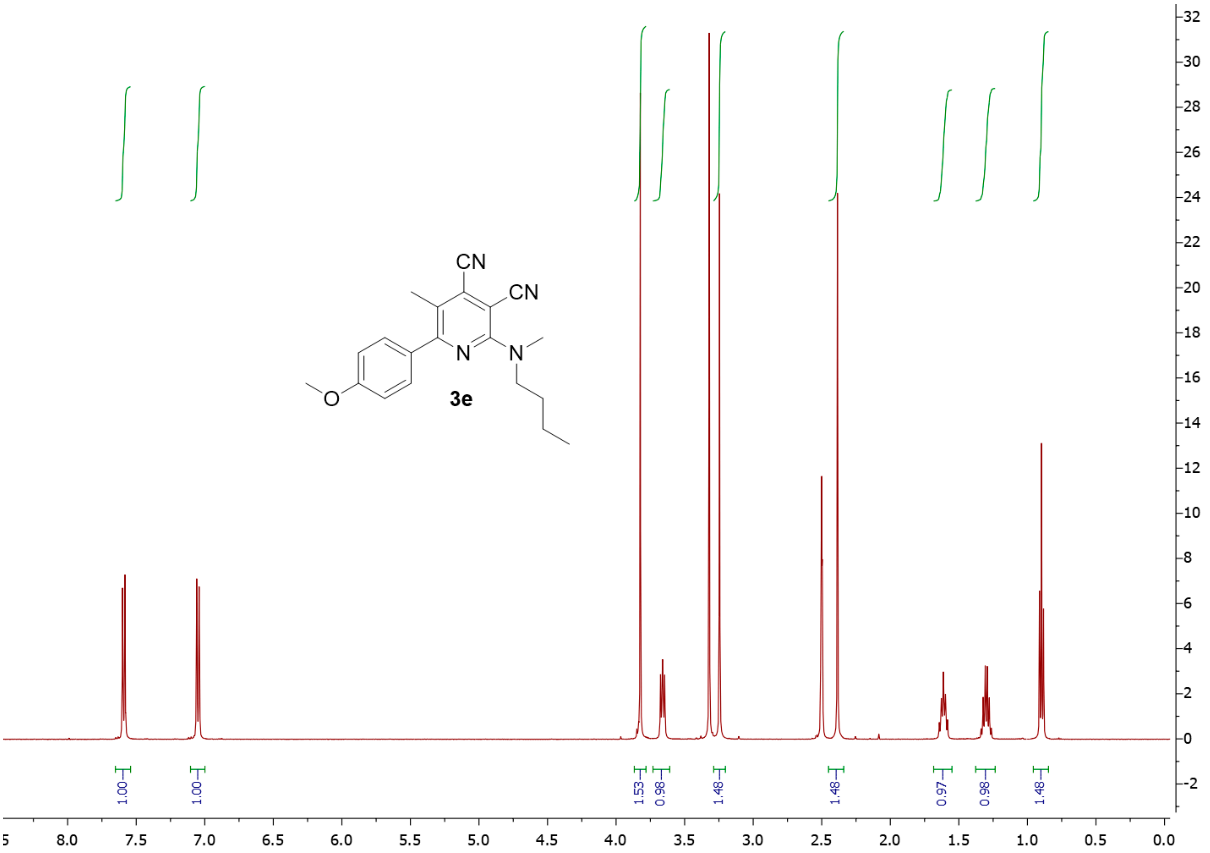

Figure S21. ^{1}H NMR-spectrum of **3e** (500.13 MHz, DMSO-d6, 298K)

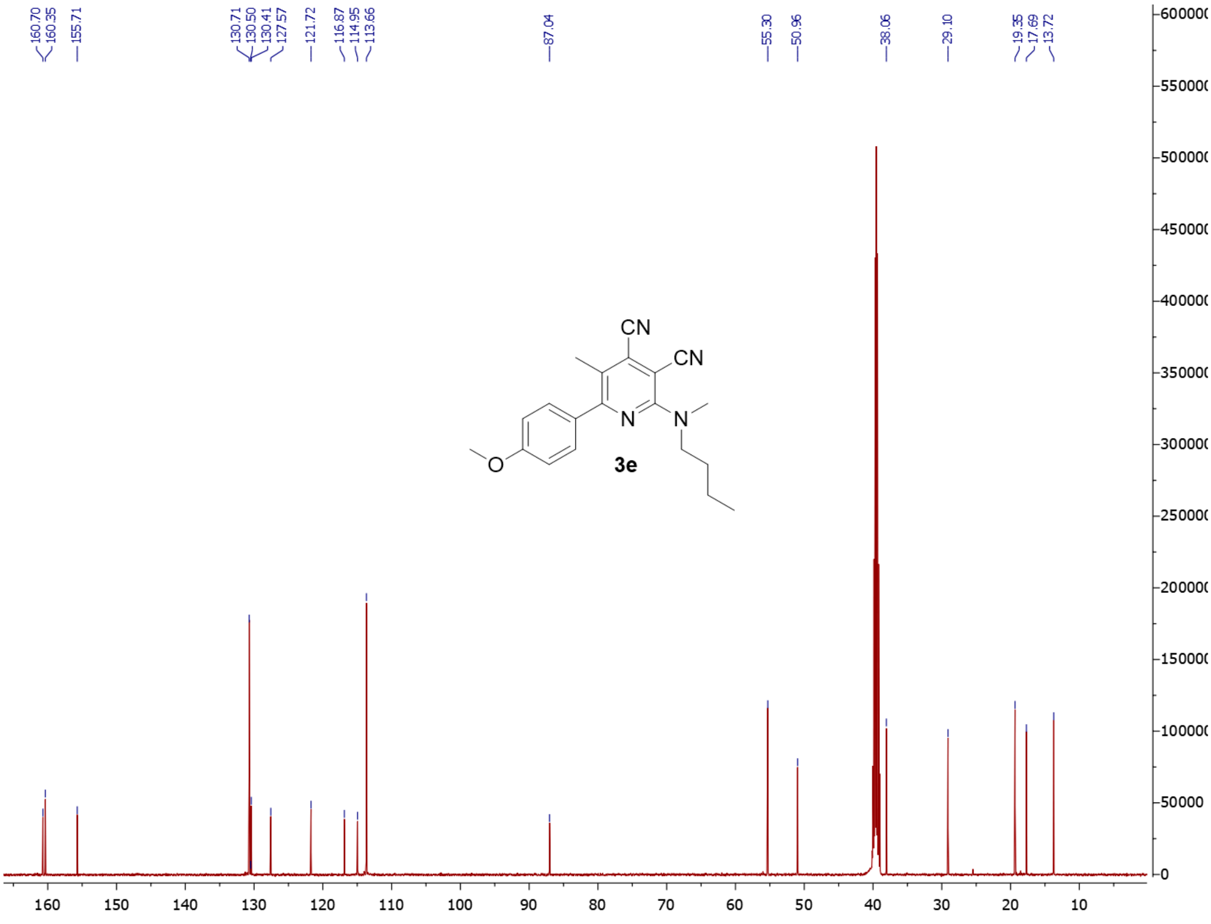

Figure S22. ^{13}C NMR-spectrum of **3e** (125.76 MHz, DMSO-d6, 299K)

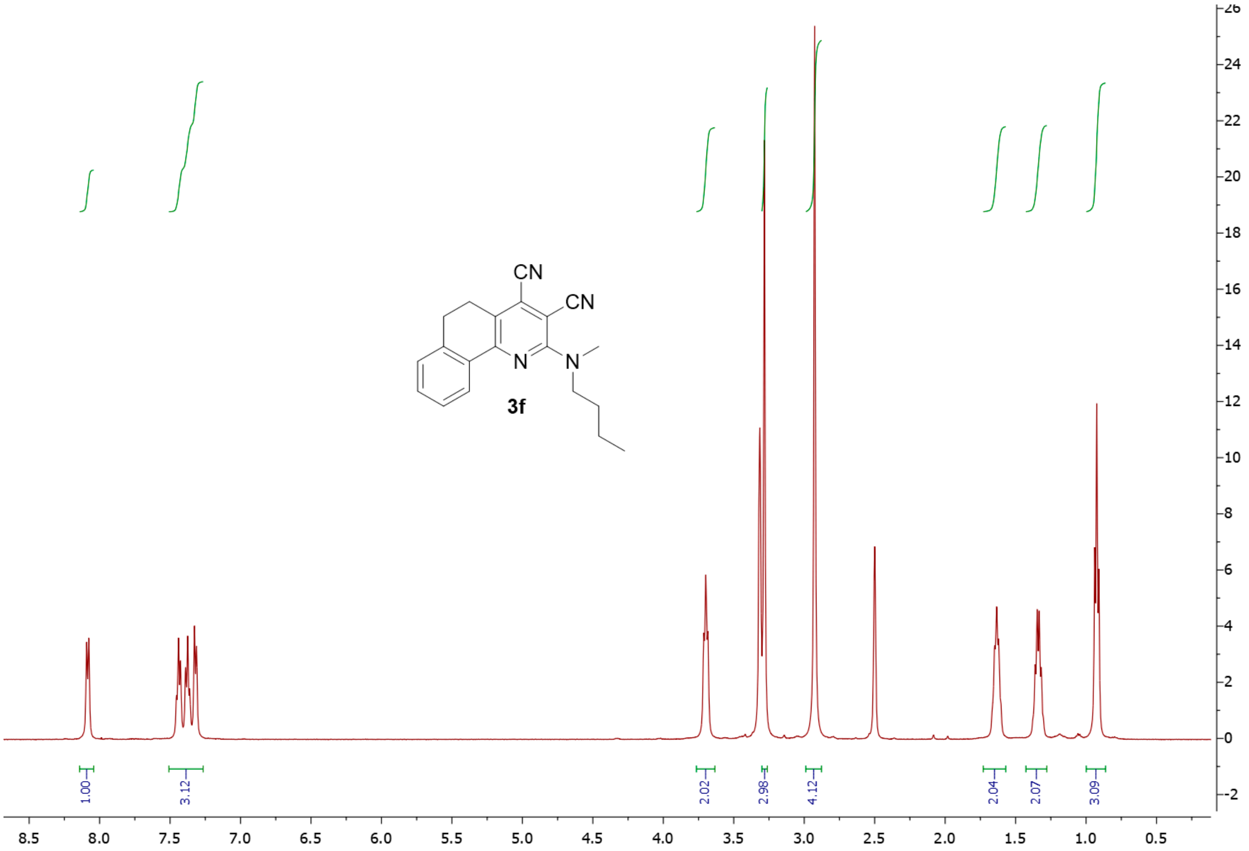

Figure S23. ^{1}H NMR-spectrum of **3f** (500.13 MHz, DMSO-d6, 298K)

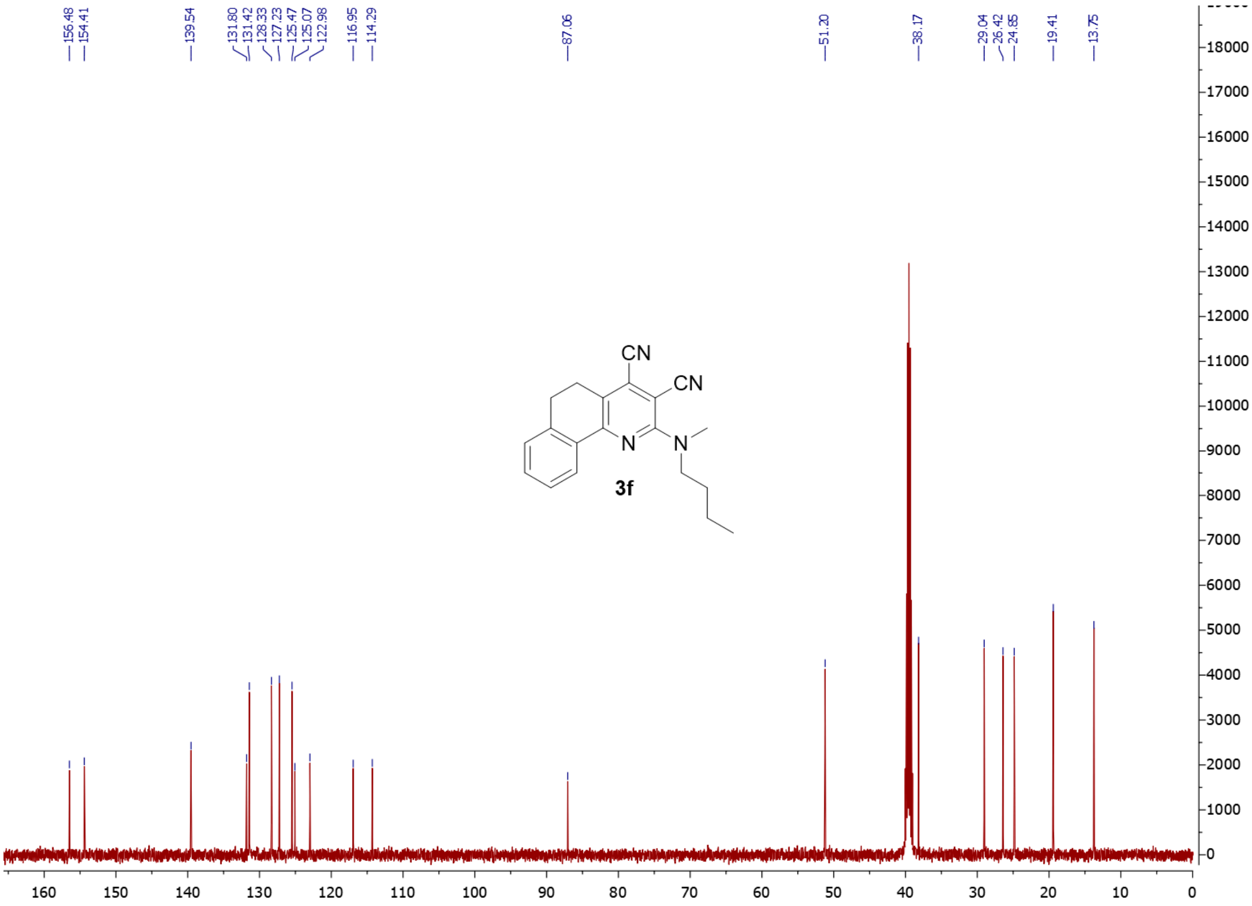

Figure S24. ^{13}C NMR-spectrum of **3f** (125.76 MHz, DMSO-d6, 299K)

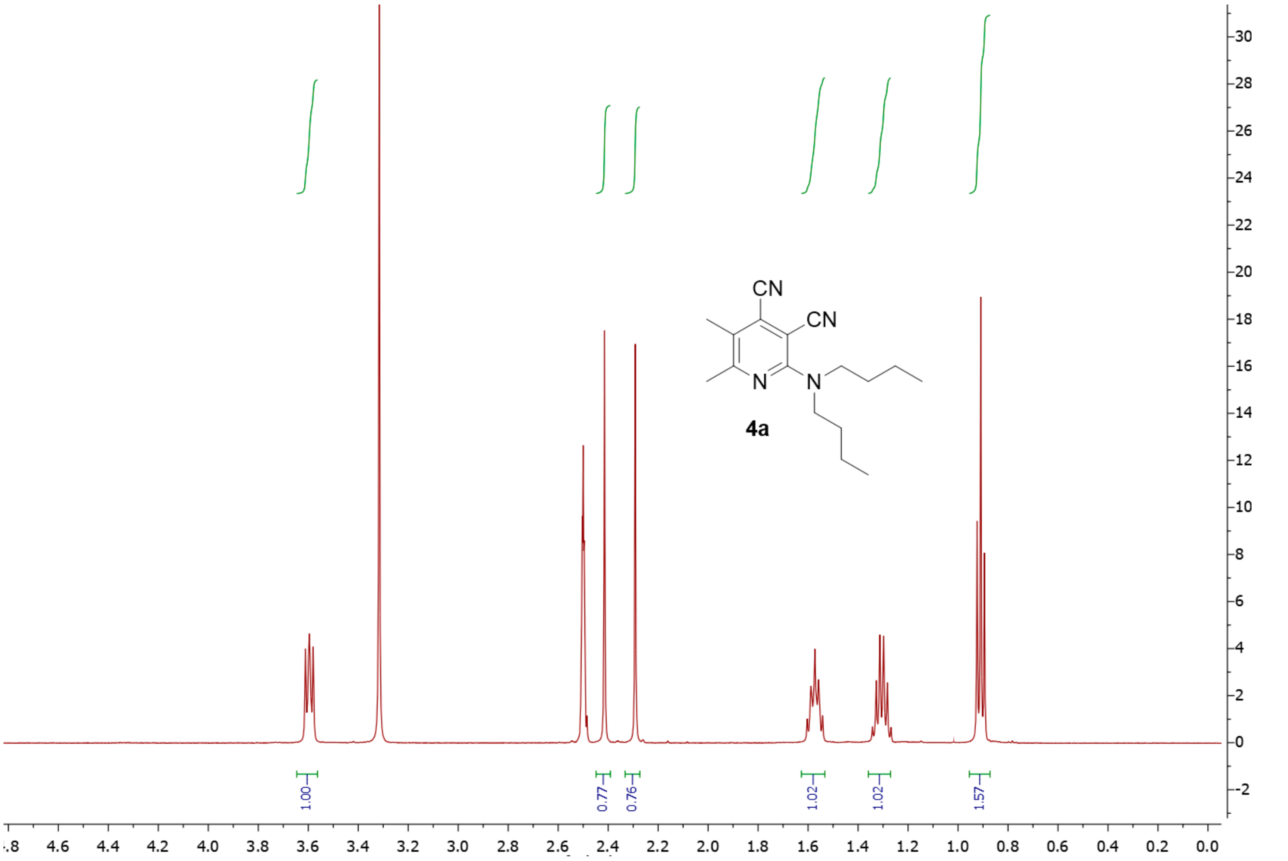

Figure S25. ^{1}H NMR-spectrum of **4a** (500.13 MHz, DMSO-d6, 298K)

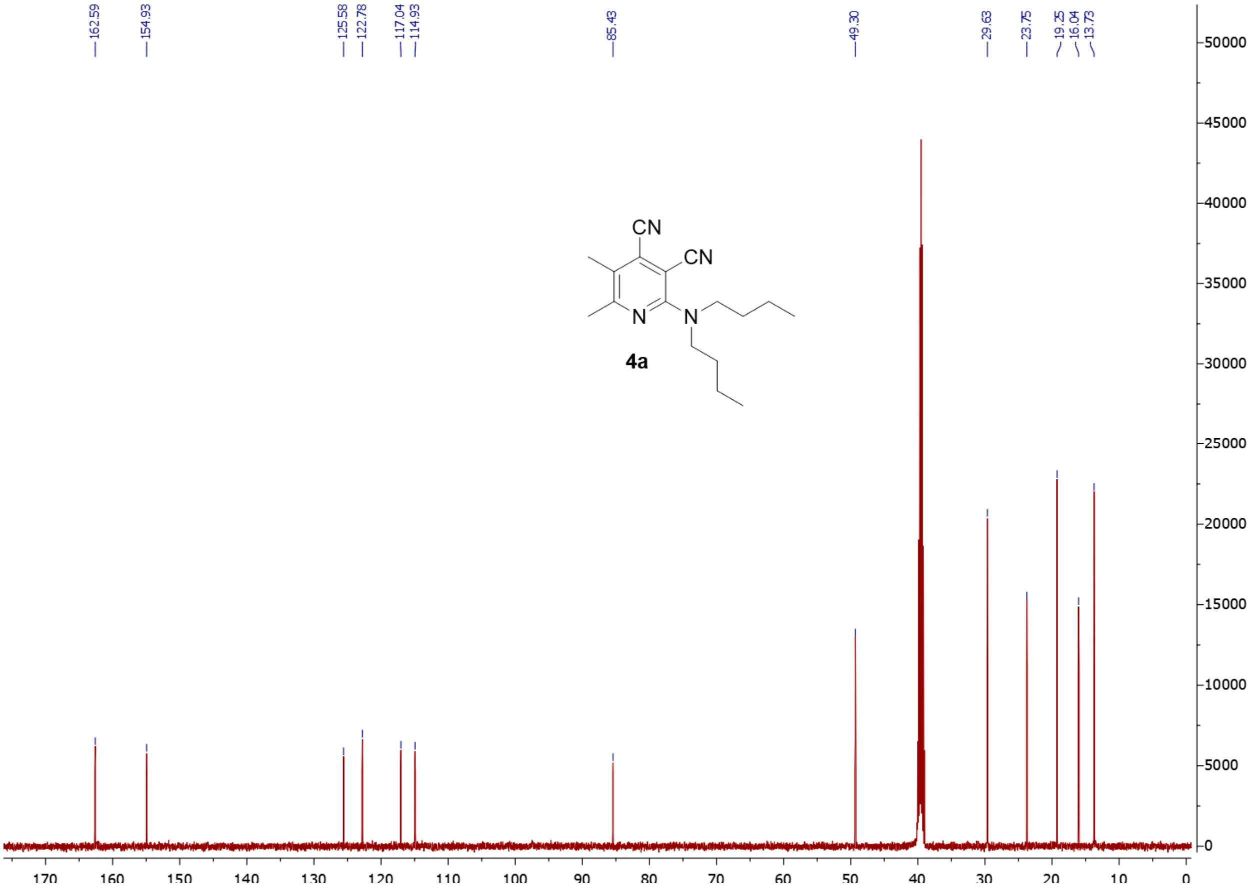

Figure S26. ^{13}C NMR-spectrum of **4a** (125.76 MHz, DMSO-d6, 299K)

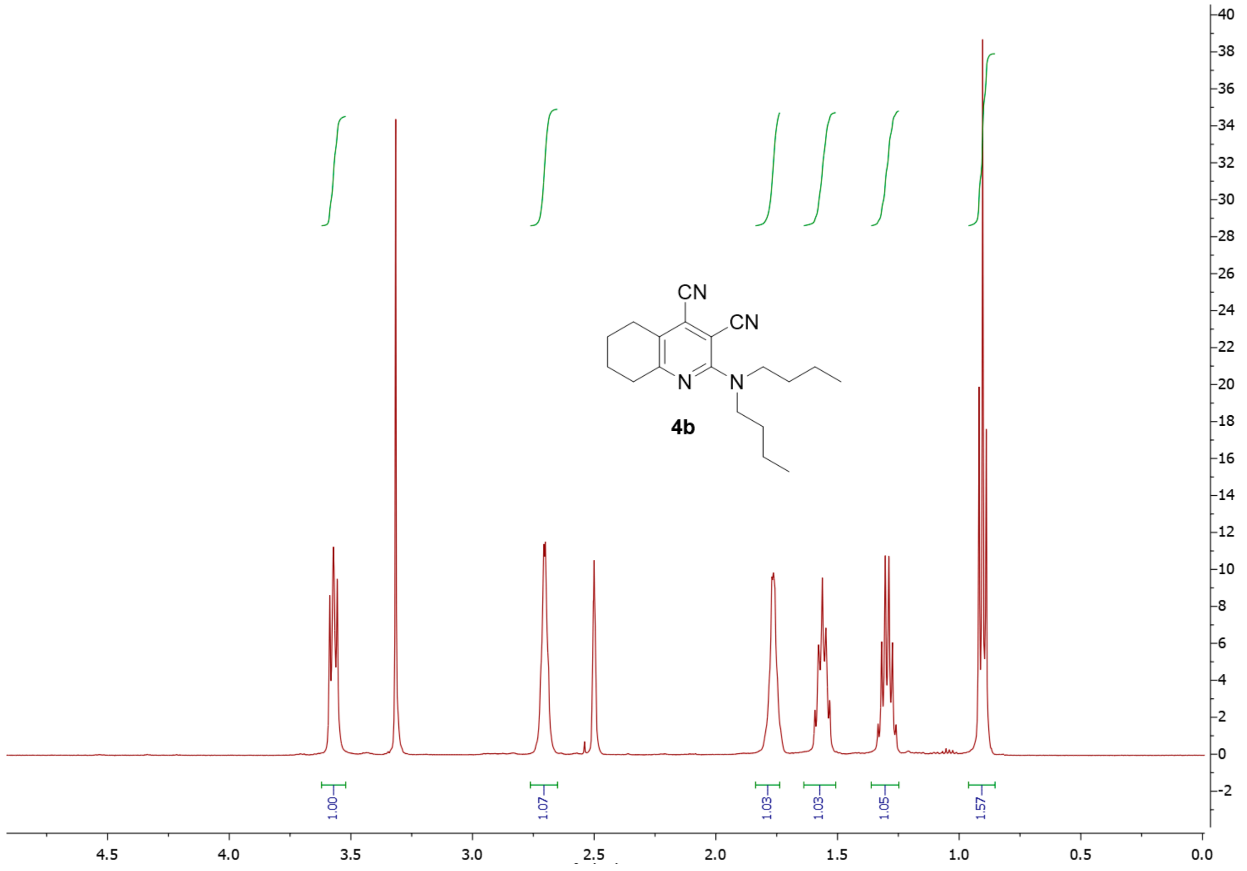

Figure S27. ^{1}H NMR-spectrum of **4b** (500.13 MHz, DMSO-d6, 298K)

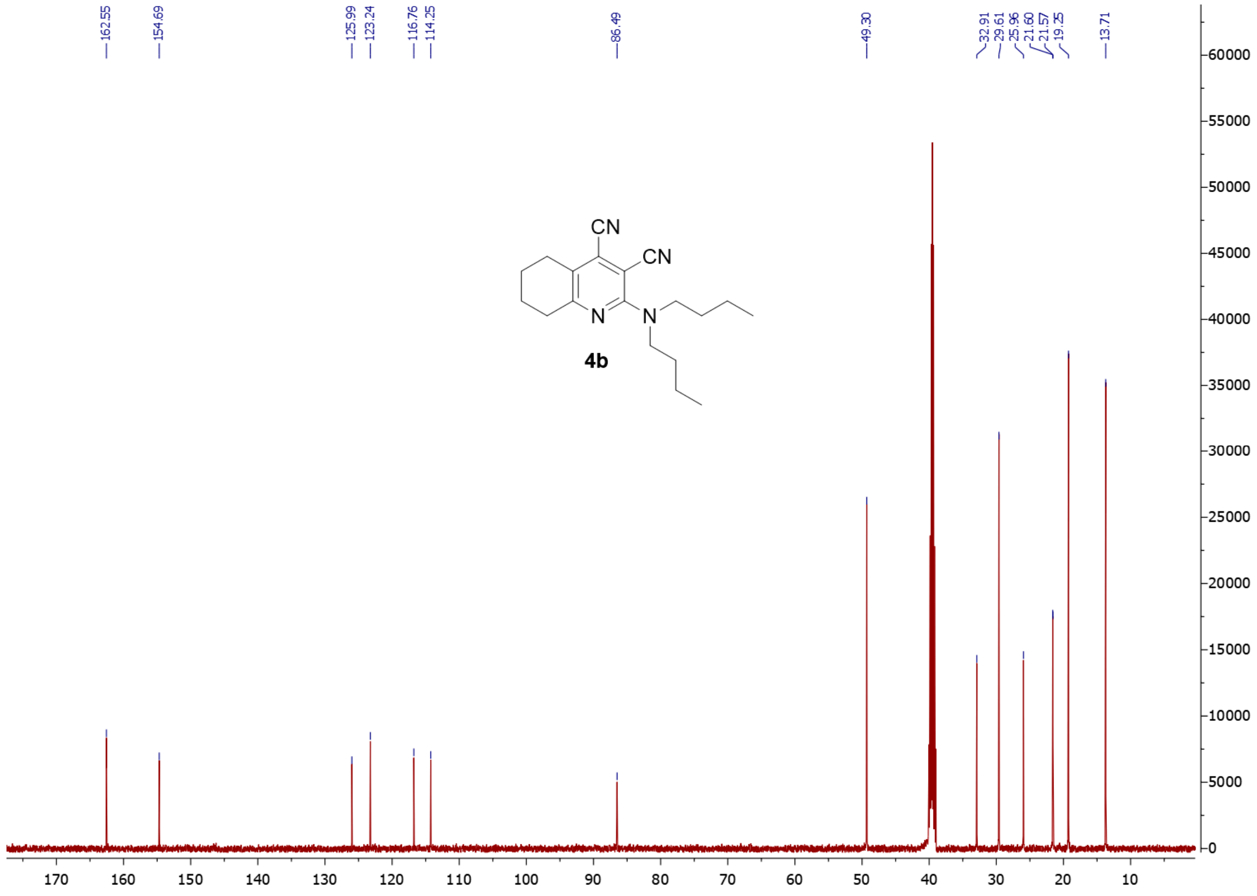

Figure S28. ^{13}C NMR-spectrum of **4b** (125.76 MHz, DMSO-d6, 299K)

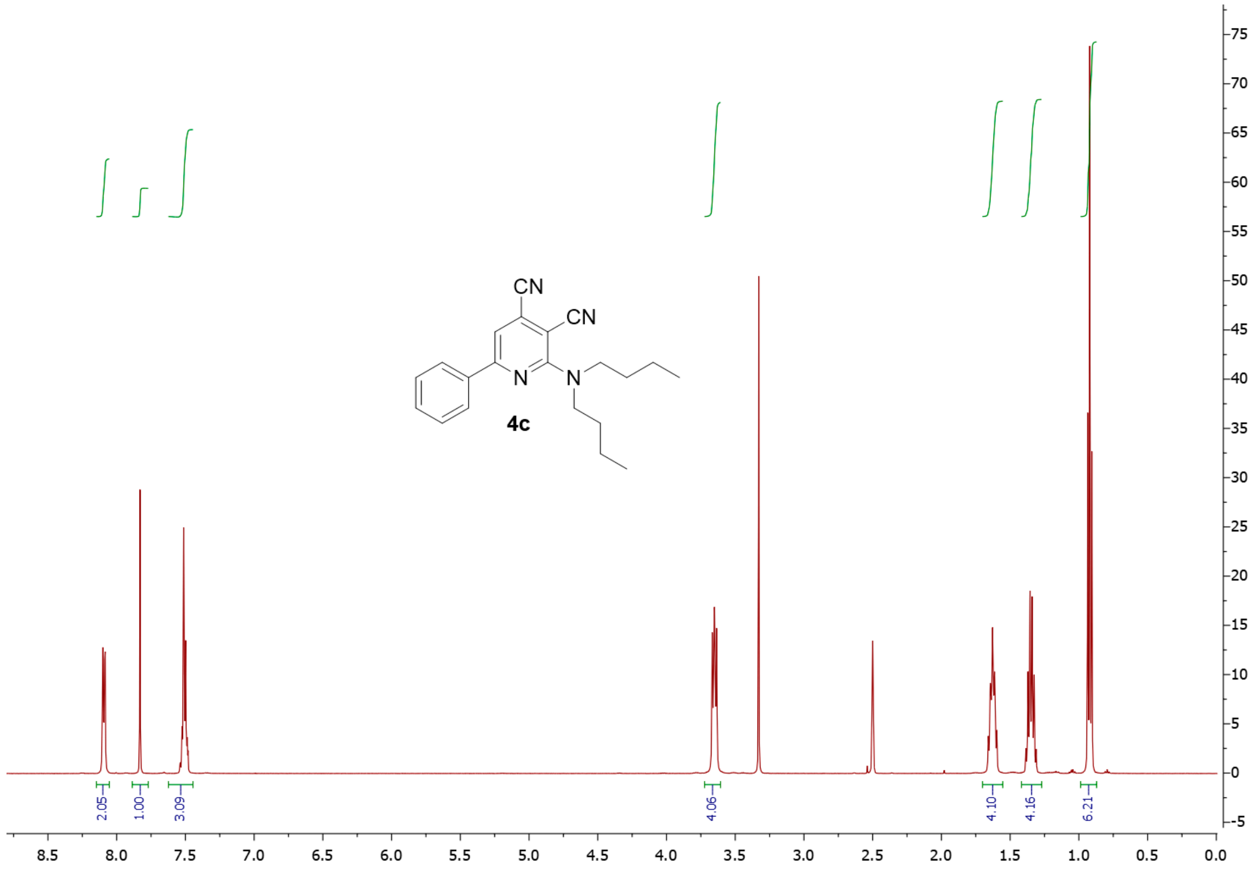

Figure S29. ^{1}H NMR-spectrum of **4c** (500.13 MHz, DMSO-d6, 298K)

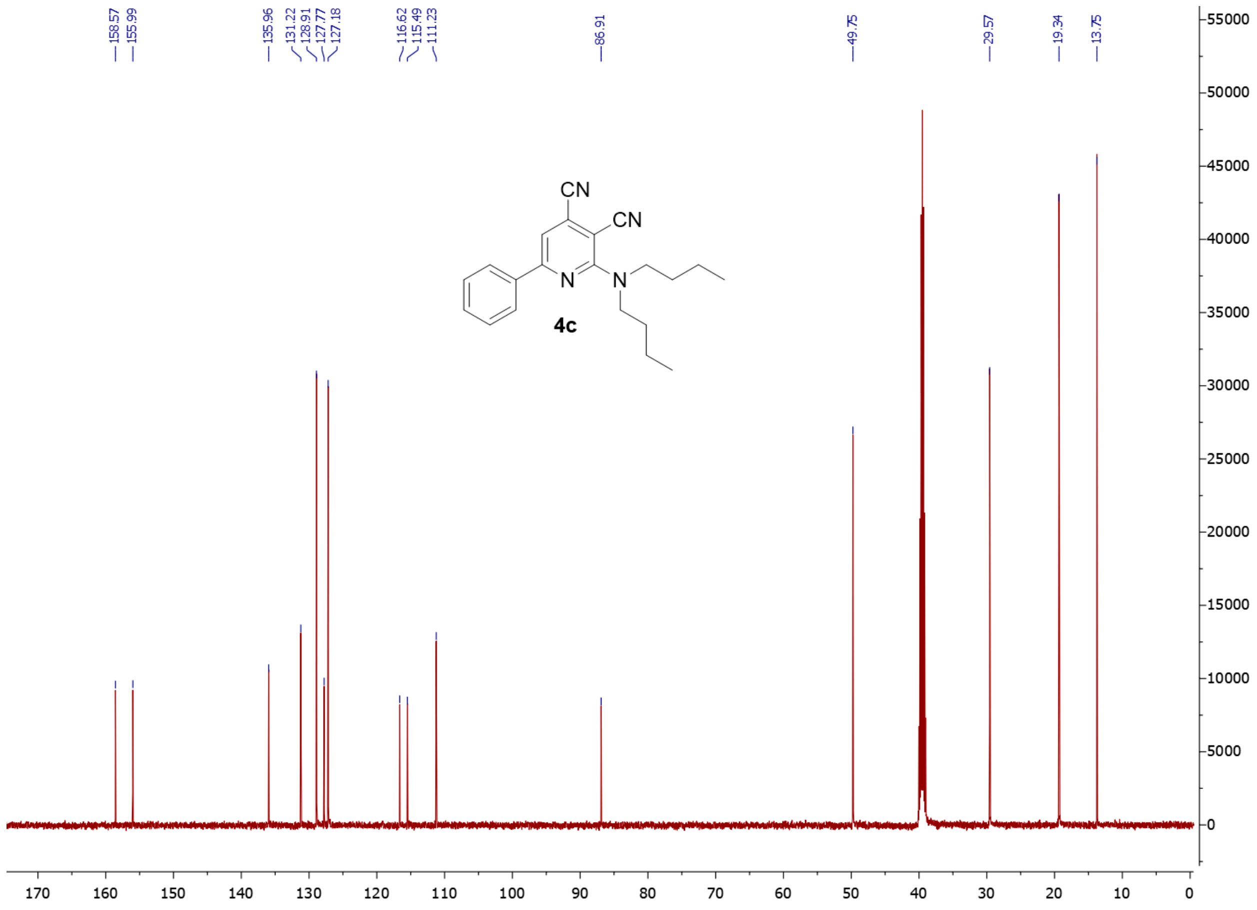

Figure S30. ^{13}C NMR-spectrum of **4c** (125.76 MHz, DMSO-d6, 299K)

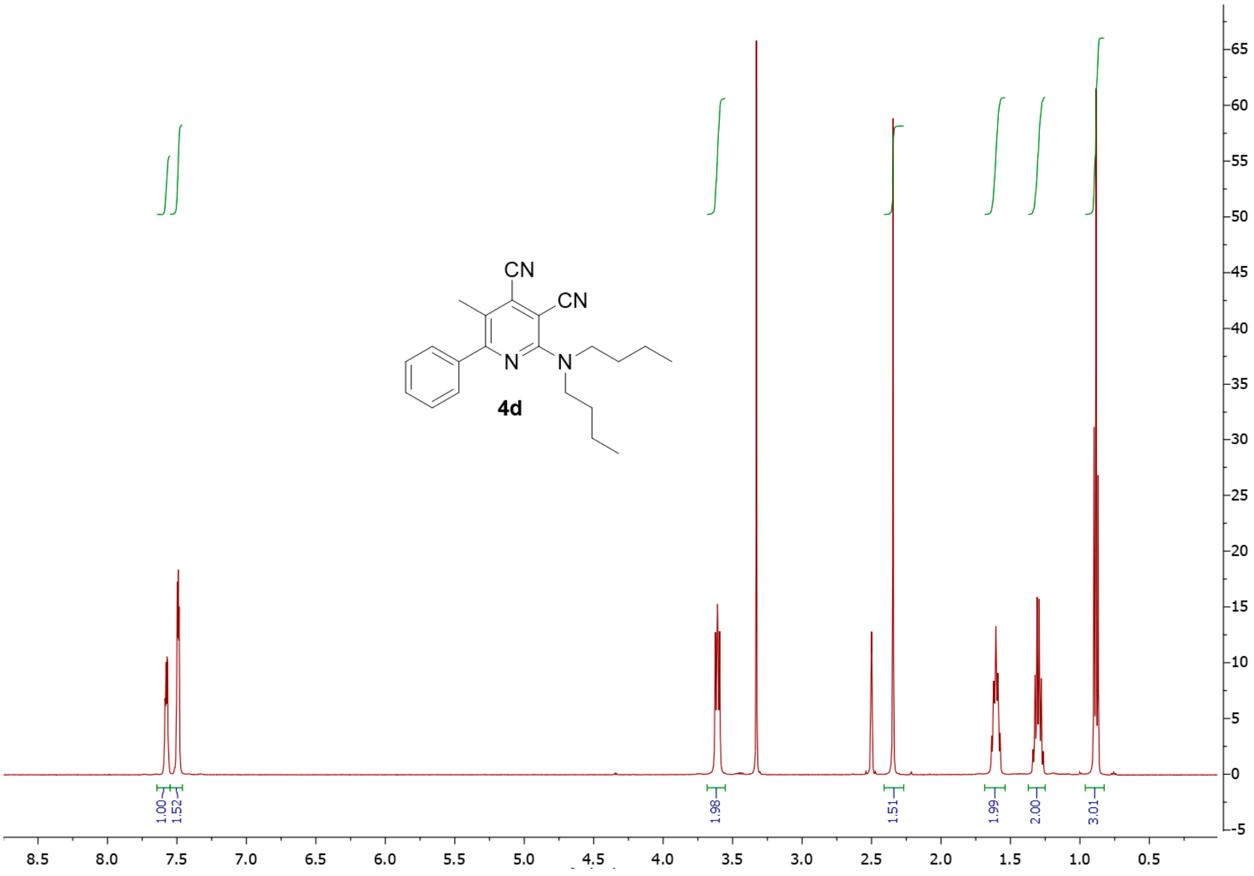

Figure S31. ^{1}H NMR-spectrum of **4d** (500.13 MHz, DMSO-d6, 298K)

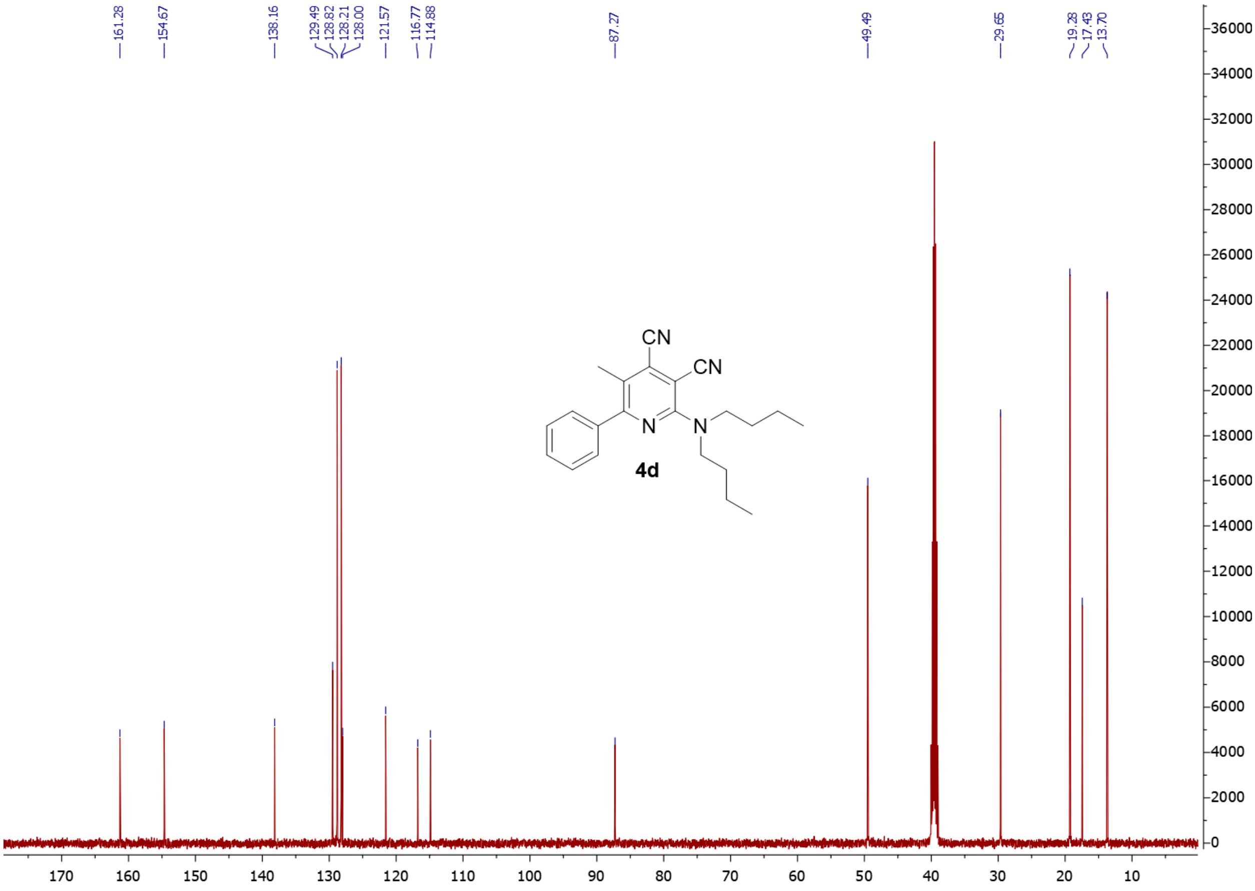

Figure S32. ^{13}C NMR-spectrum of **4d** (125.76 MHz, DMSO-d6, 299K)

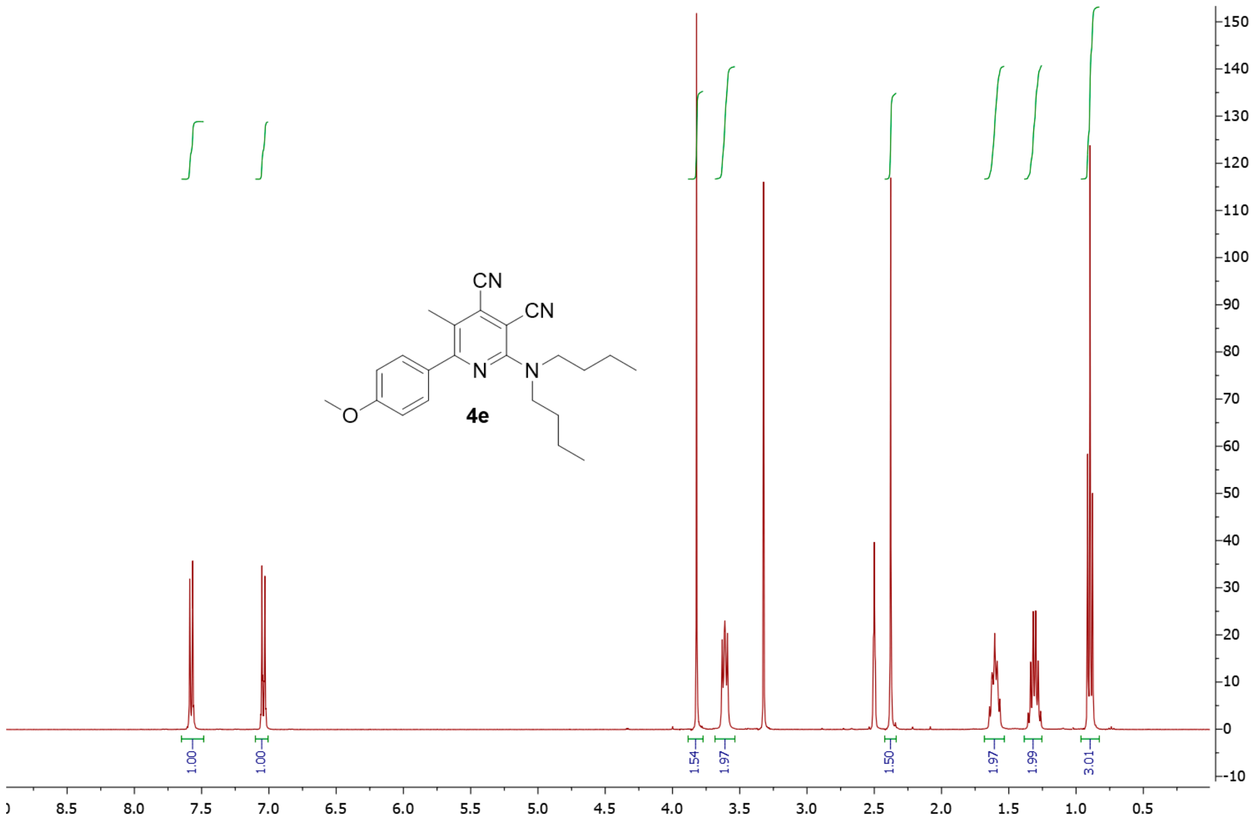

Figure S33. ^{1}H NMR-spectrum of **4e** (500.13 MHz, DMSO-d6, 298K)

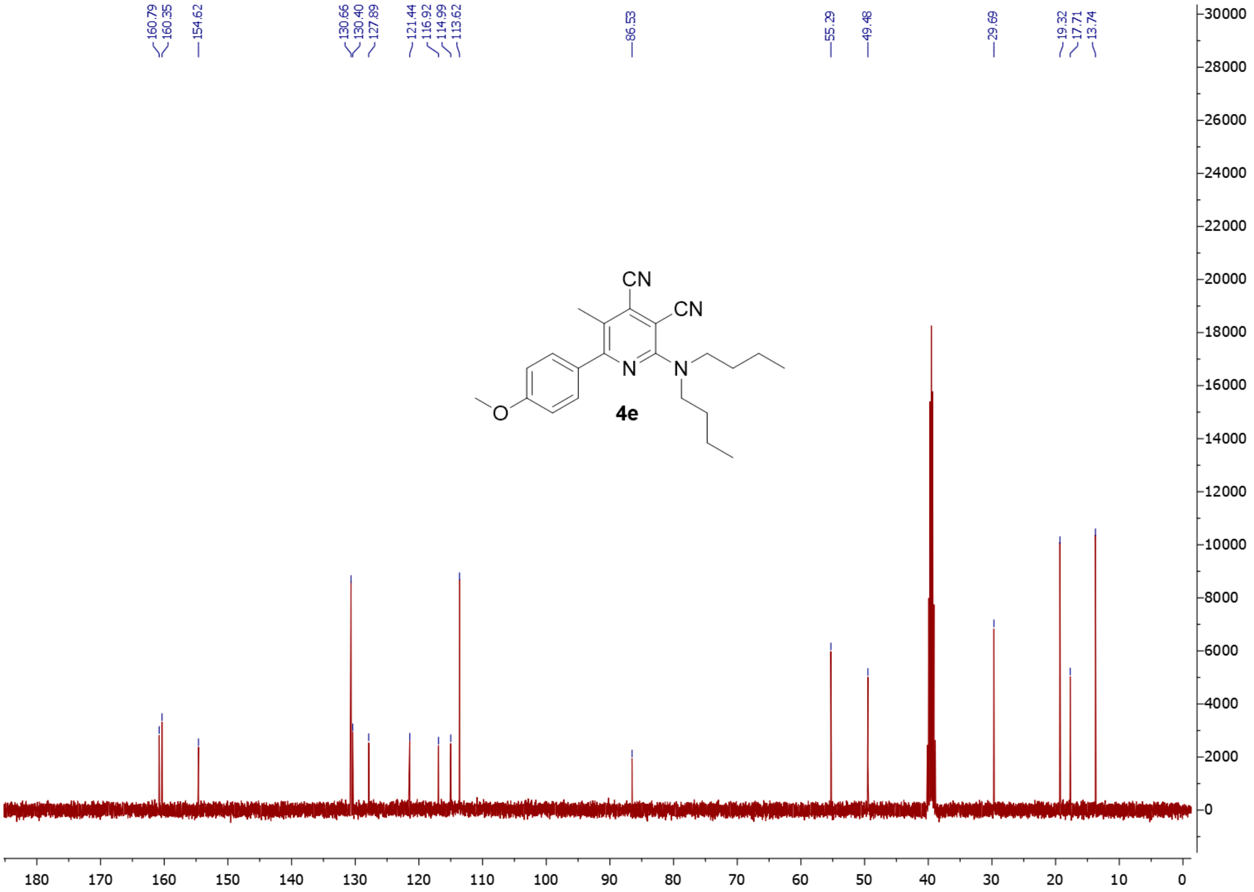

Figure S34. ^{13}C NMR-spectrum of **4e** (125.76 MHz, DMSO-d6, 299K)

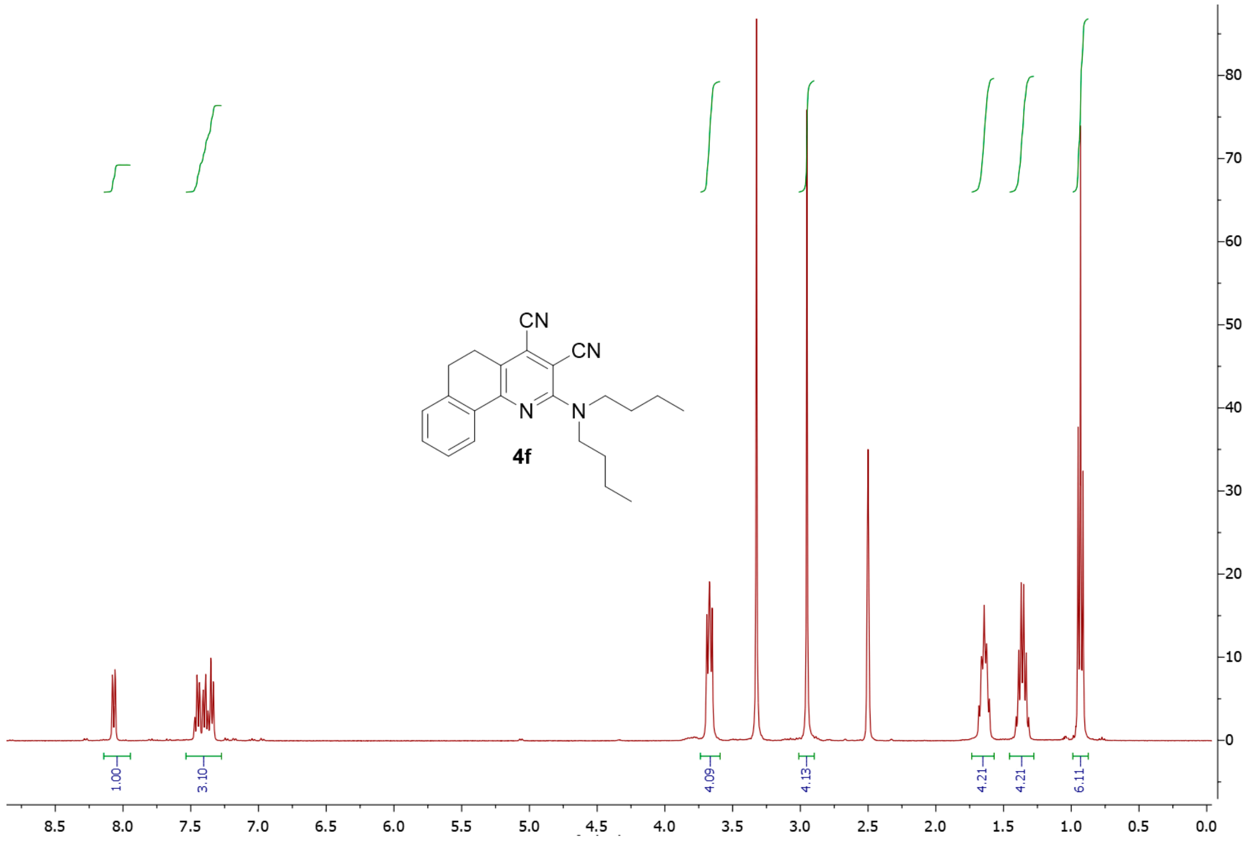

Figure S35. ^{1}H NMR-spectrum of **4f** (500.13 MHz, DMSO-d6, 298K)

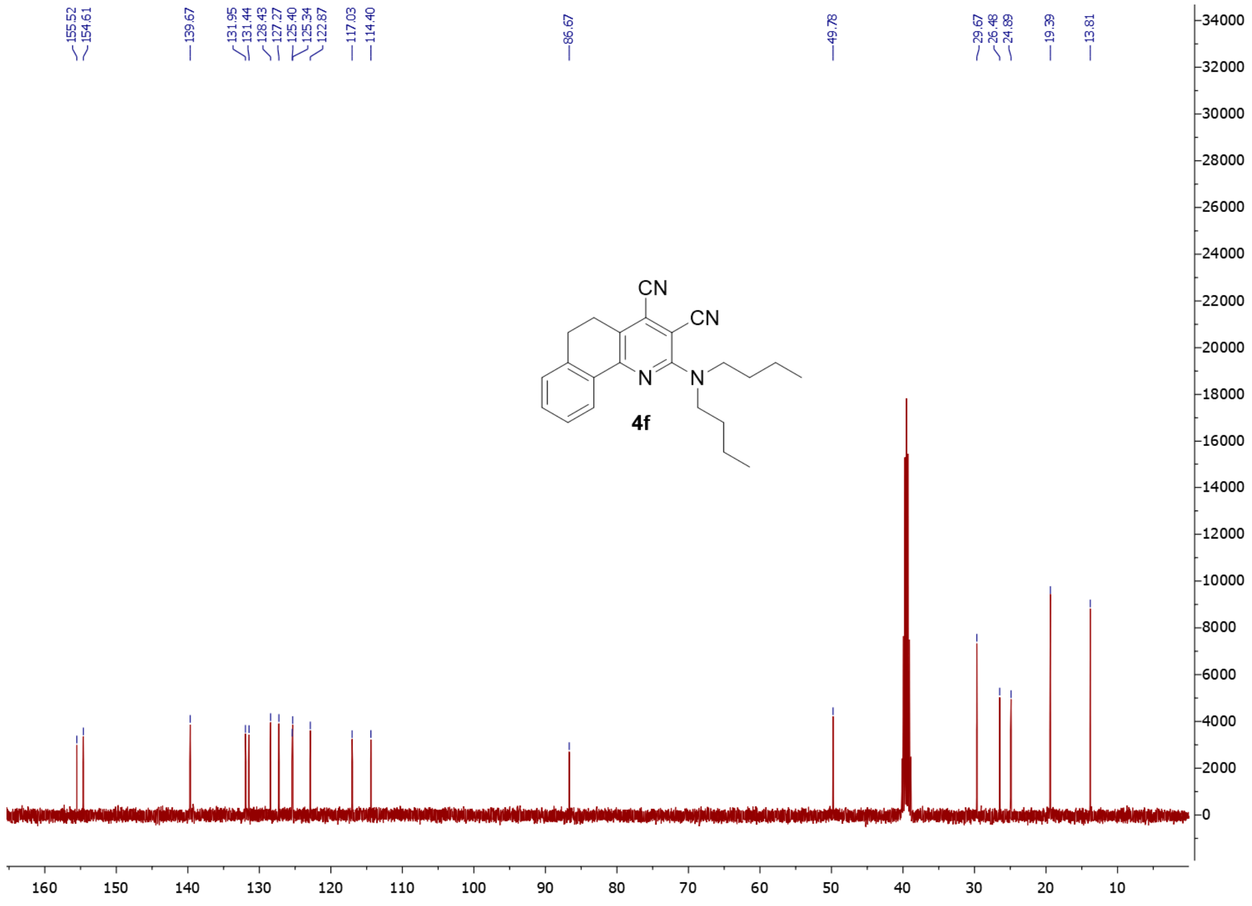

Figure S36. ^{13}C NMR-spectrum of **4f** (125.76 MHz, DMSO-d6, 299K)

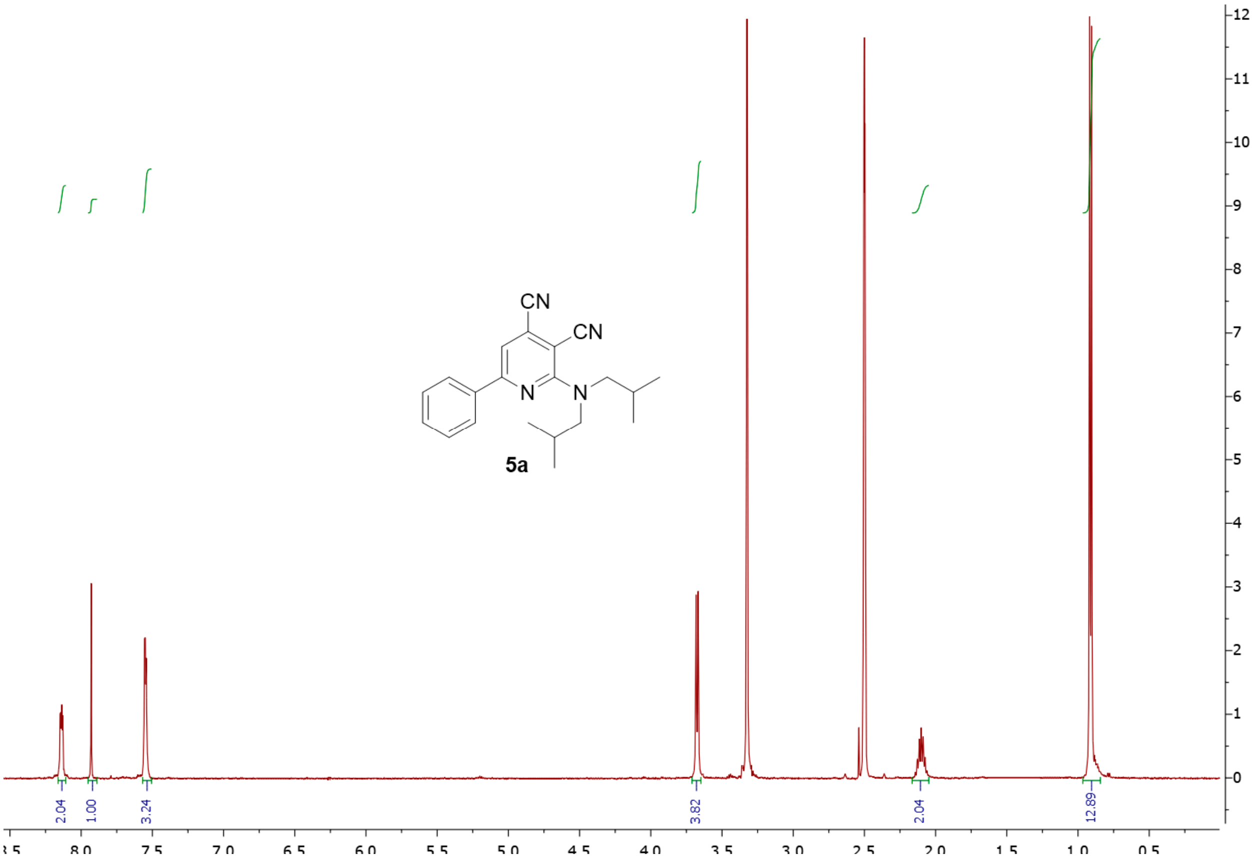

Figure S37. ^{1}H NMR-spectrum of **5a** (500.13 MHz, DMSO-d6, 298K)

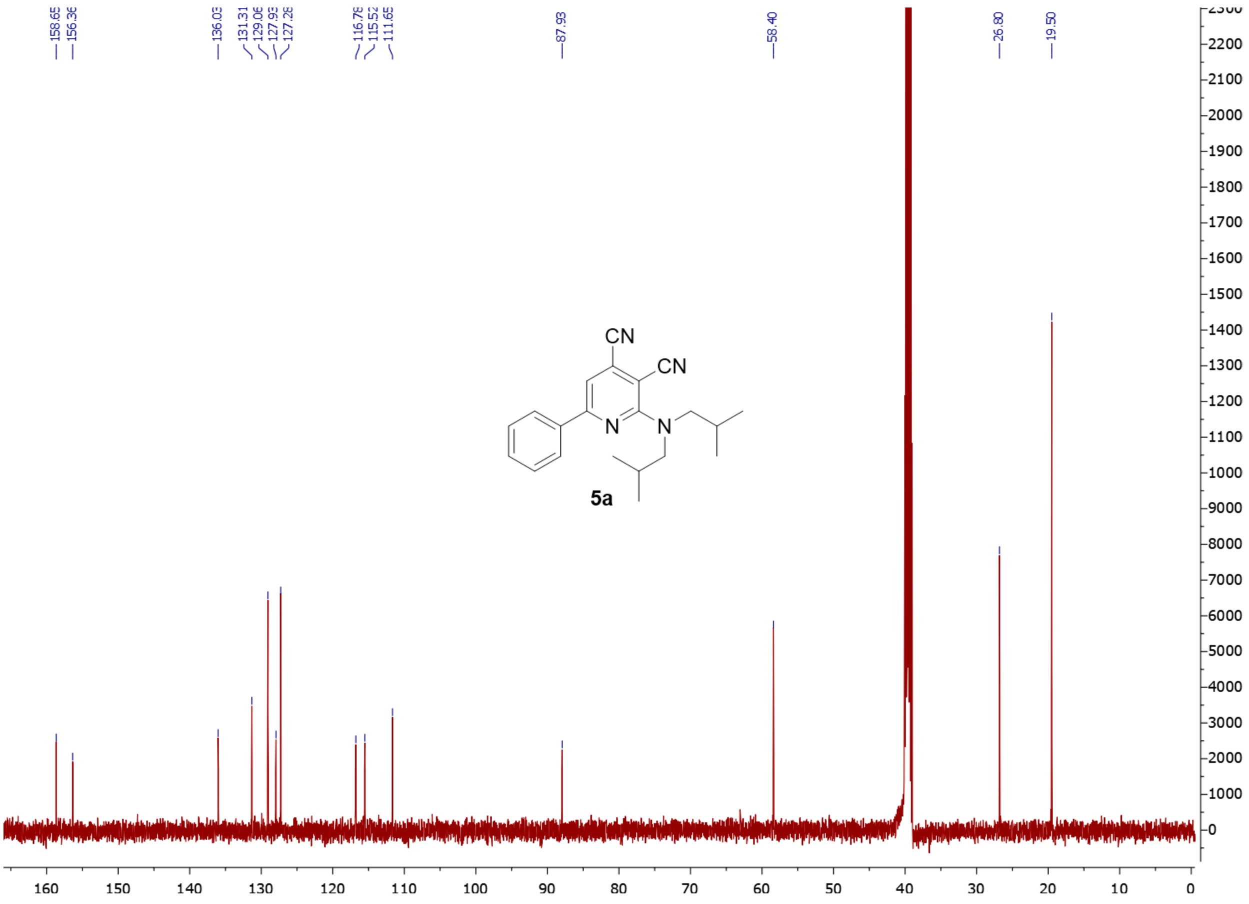

Figure S38. ^{13}C NMR-spectrum of **5a** (125.76 MHz, DMSO-d6, 299K)

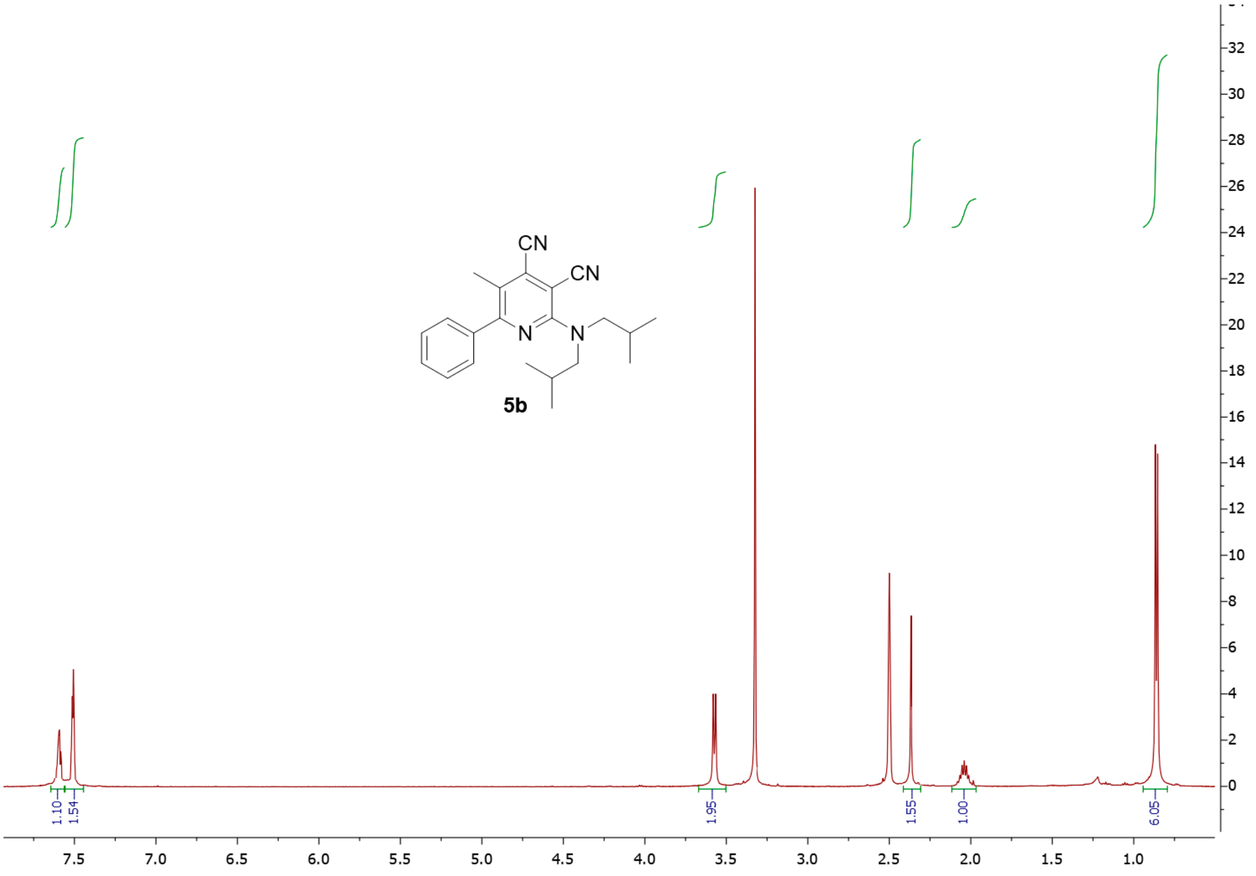

Figure S39. ^{1}H NMR-spectrum of **5b** (500.13 MHz, DMSO-d6, 298K)

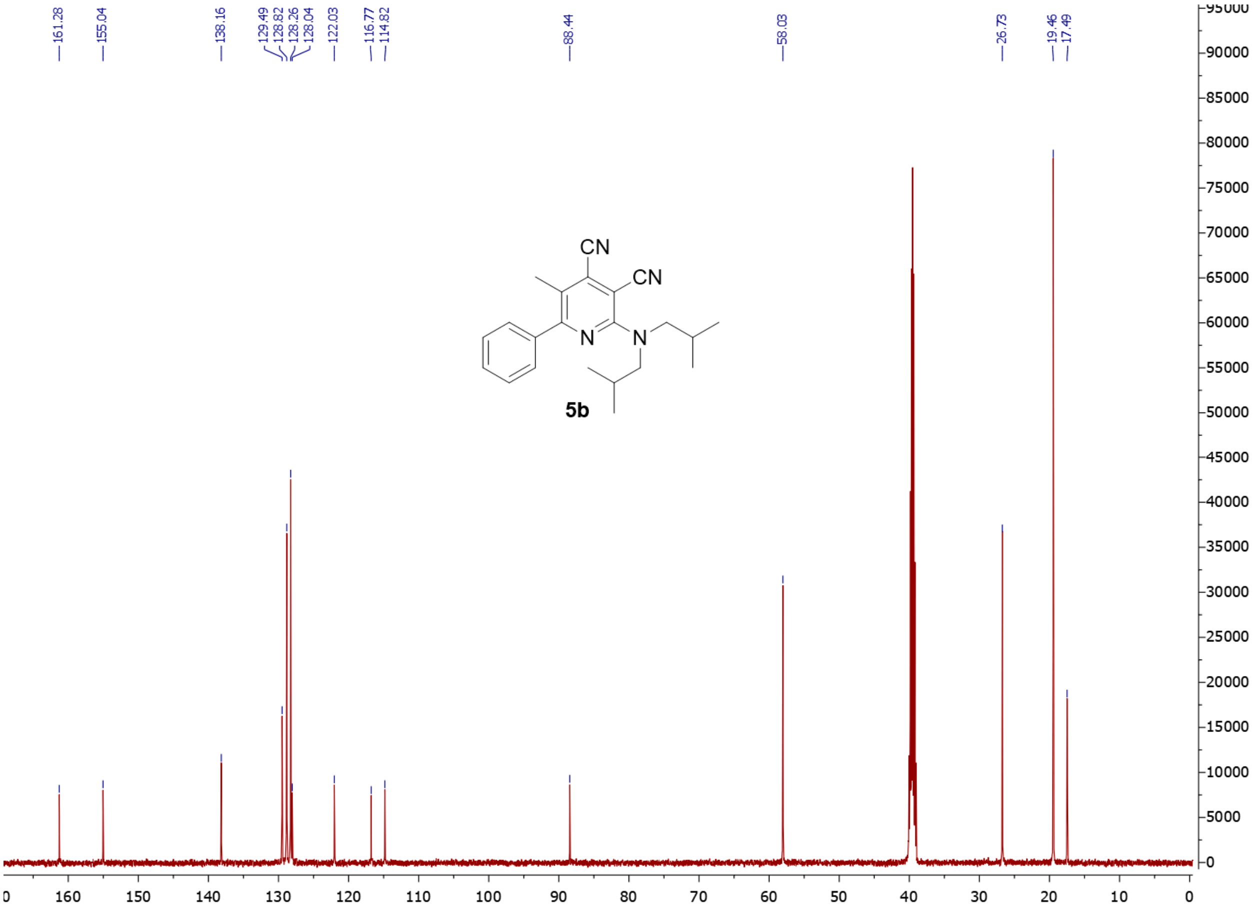

Figure S40. ^{13}C NMR-spectrum of **5b** (125.76 MHz, DMSO-d6, 299K)

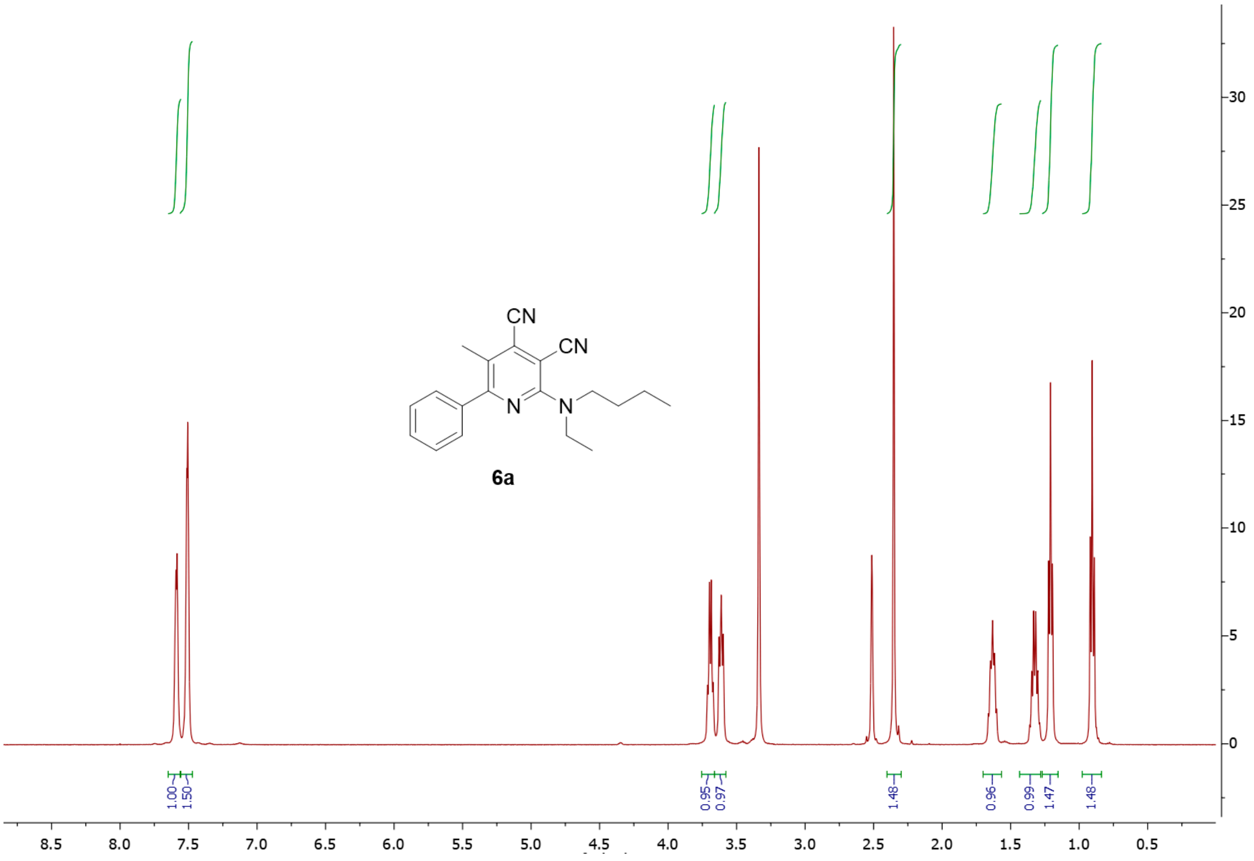

Figure S41. ^{1}H NMR-spectrum of **6a** (500.13 MHz, DMSO-d6, 298K)

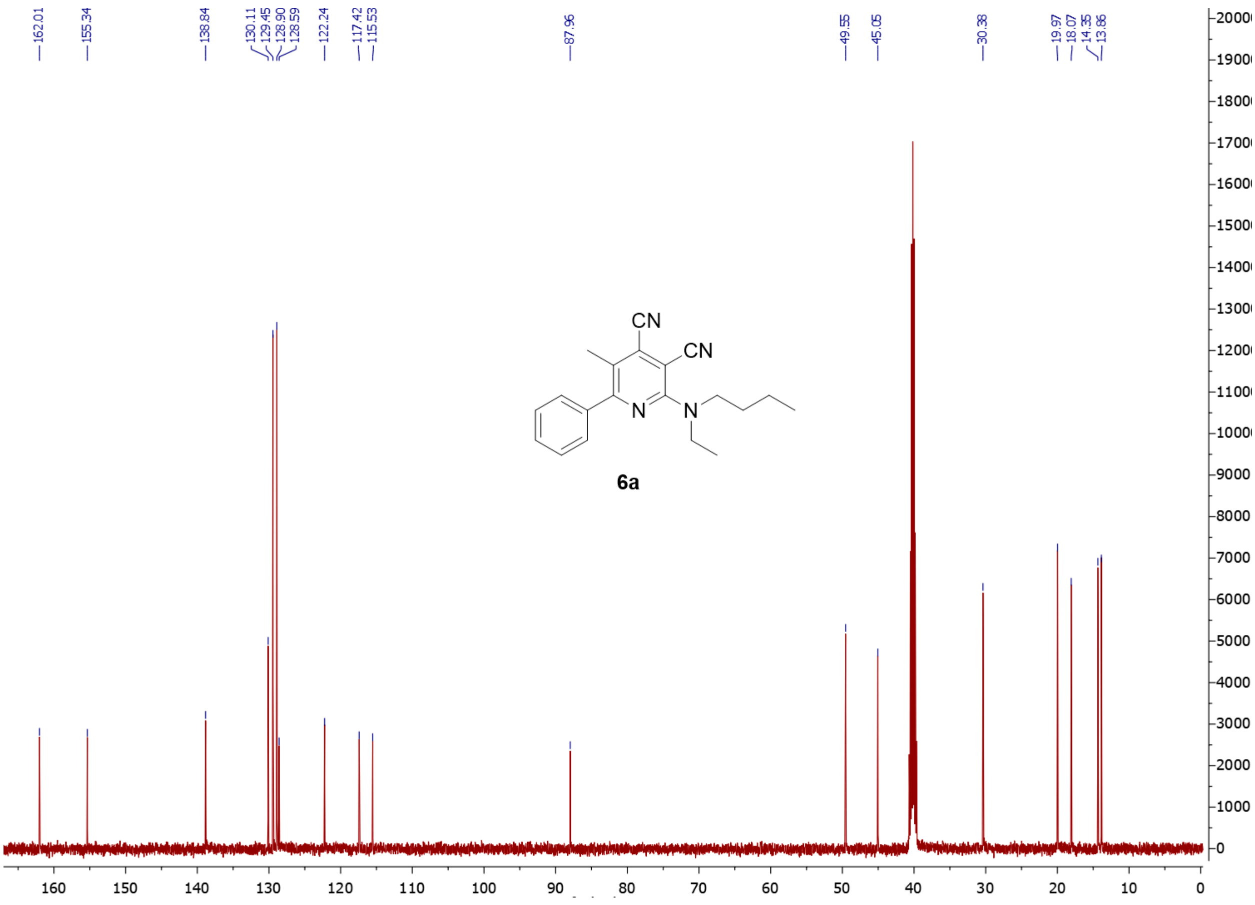

Figure S42. ^{13}C NMR-spectrum of **6a** (125.76 MHz, DMSO-d6, 299K)

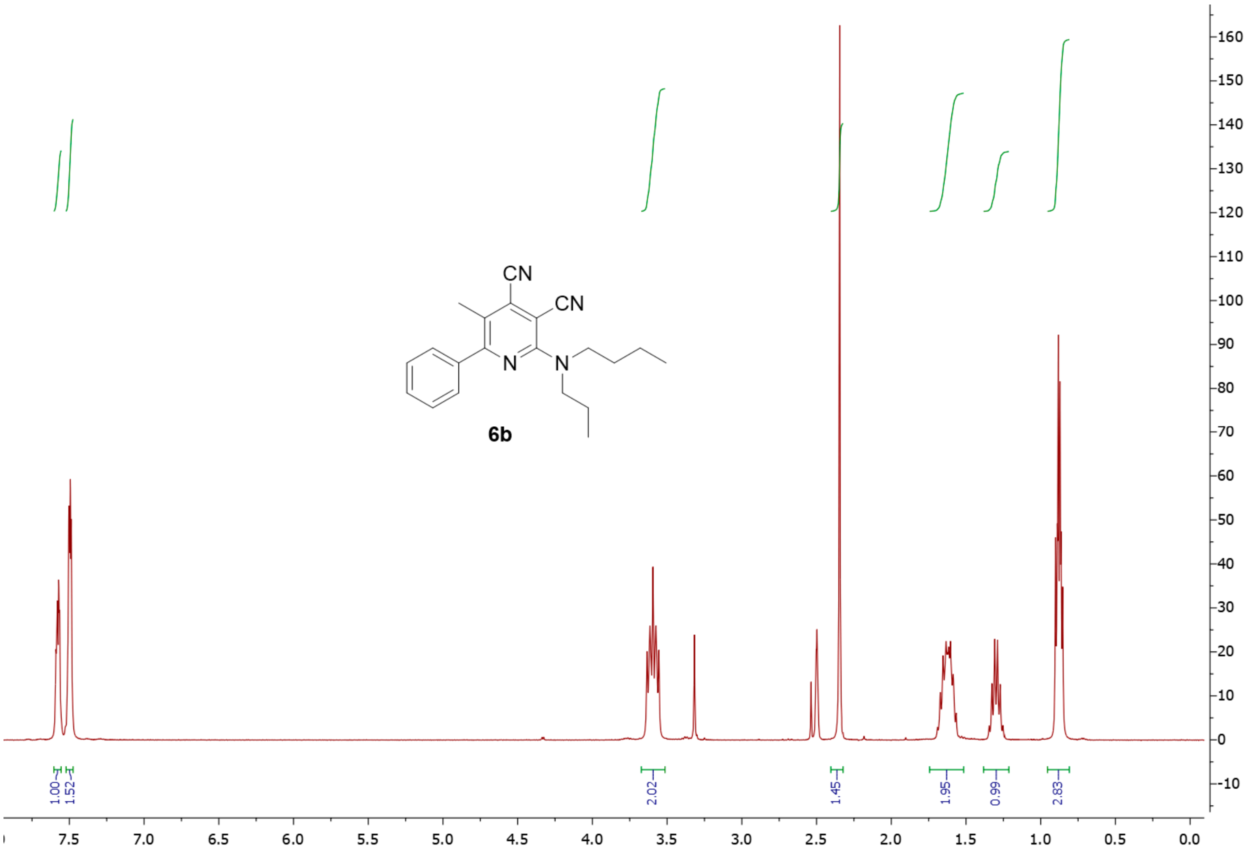

Figure S43. ^{1}H NMR-spectrum of **6b** (500.13 MHz, DMSO-d6, 298K)

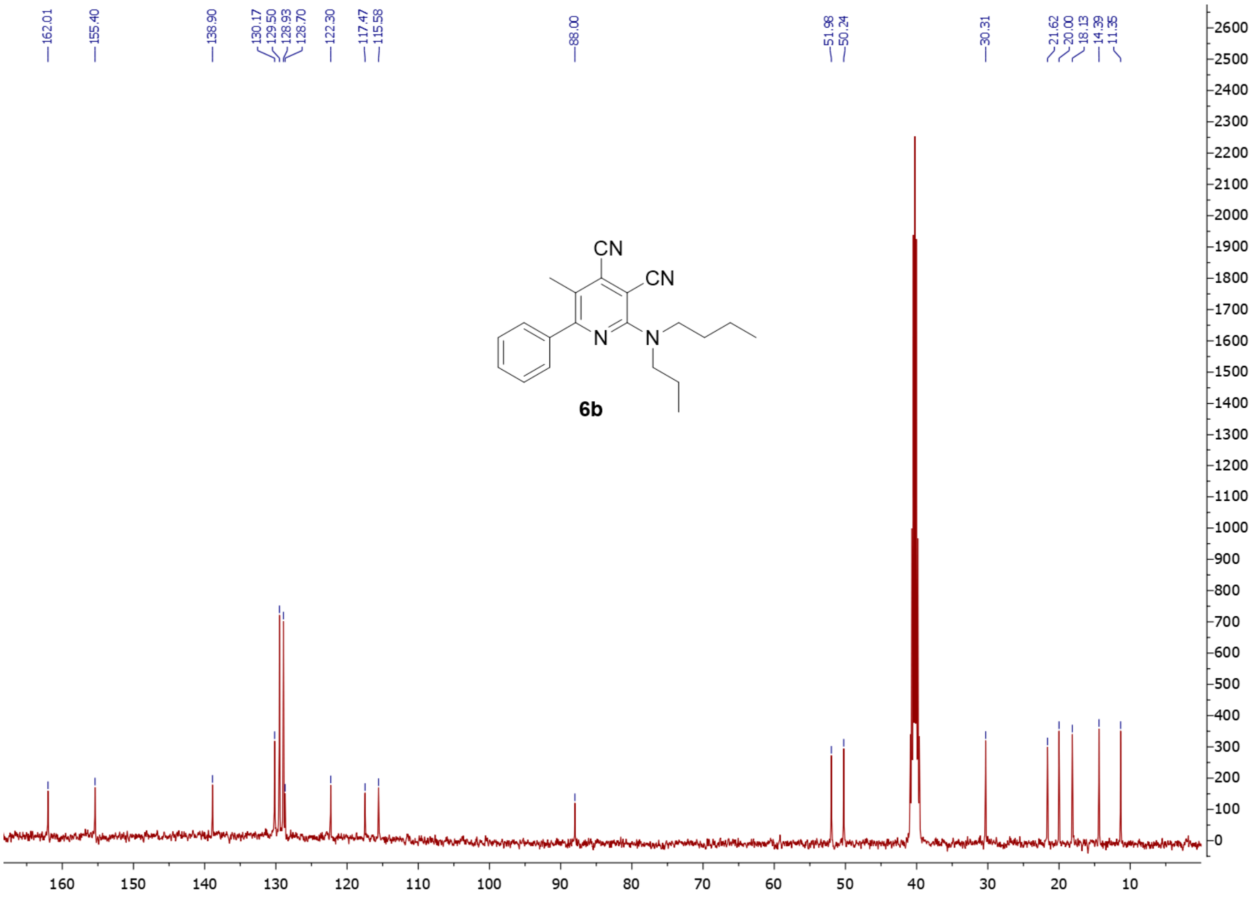

Figure S44. ^{13}C NMR-spectrum of **6b** (125.76 MHz, DMSO-d6, 299K)
